# Supplementary material for: Proteome-wide quantification of inositol pyrophosphate-protein interactions
Source: Nat Commun. 2026 Jun 4;17:4967. doi: 10.1038/s41467-026-73804-8 (PMC13237142; doi:10.1038/s41467-026-73804-8)
Supplement: Supplementary file 1 — Supplementary Information [file 41467_2026_73804_MOESM1_ESM.pdf]

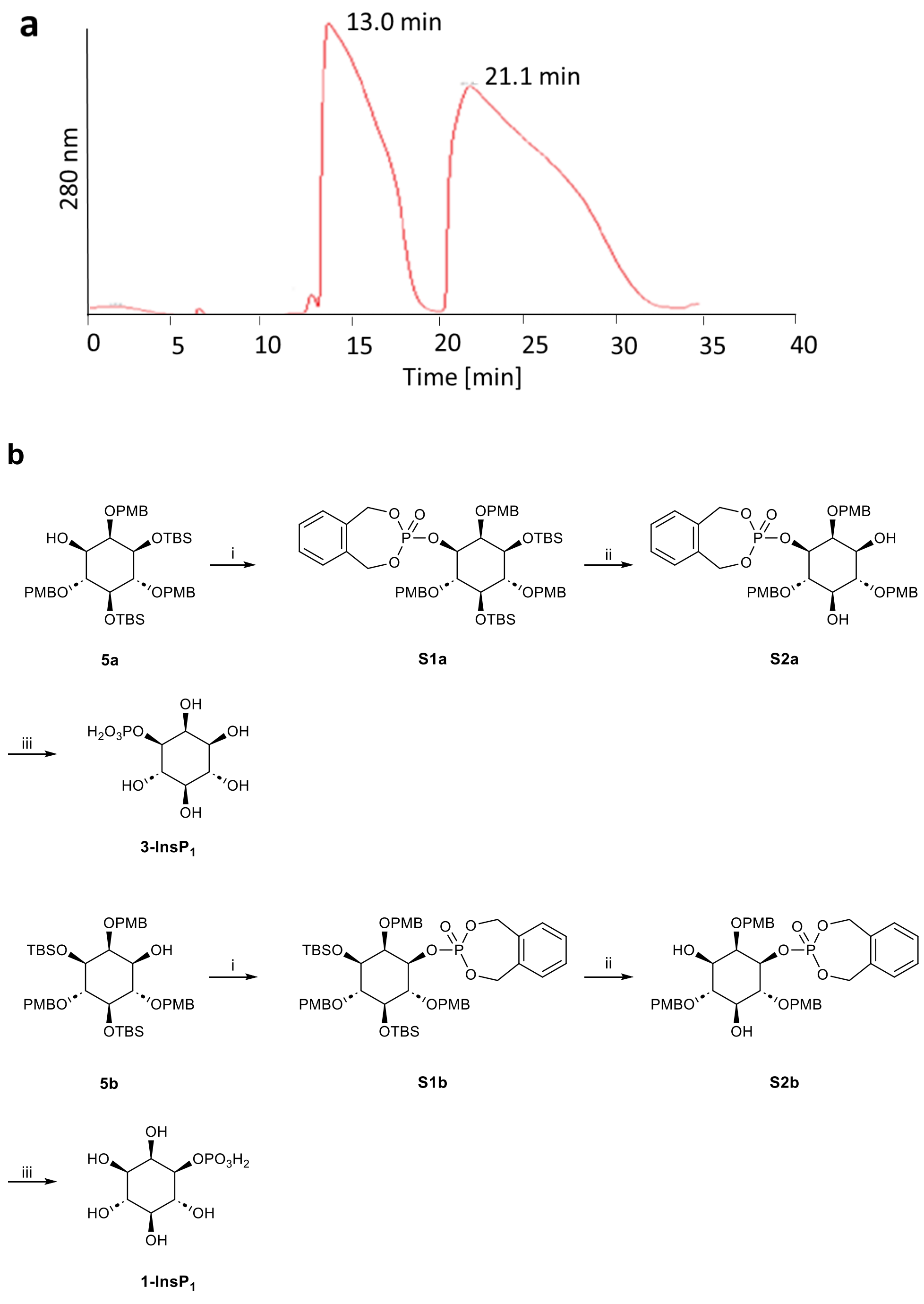

Supplementary Figure 1: Preparation and validation of enantiopure precursors.

a:Chiral separation of 200 mg enantiomer mix **5**a and **5b** on a Registech (S, S) Whelk-O 1 chiral column. **b**:Synthesis of 1/3-InsP$_1$ for assignment of **5a** and **5b**: (i) **16**, 4,5-dicyanoimidazole, ACN, then mCPBA, 89% (ii) TBAF, THF, 97% (iii) Pd/C, *t*BuOH/H$_2$O, quantitative.

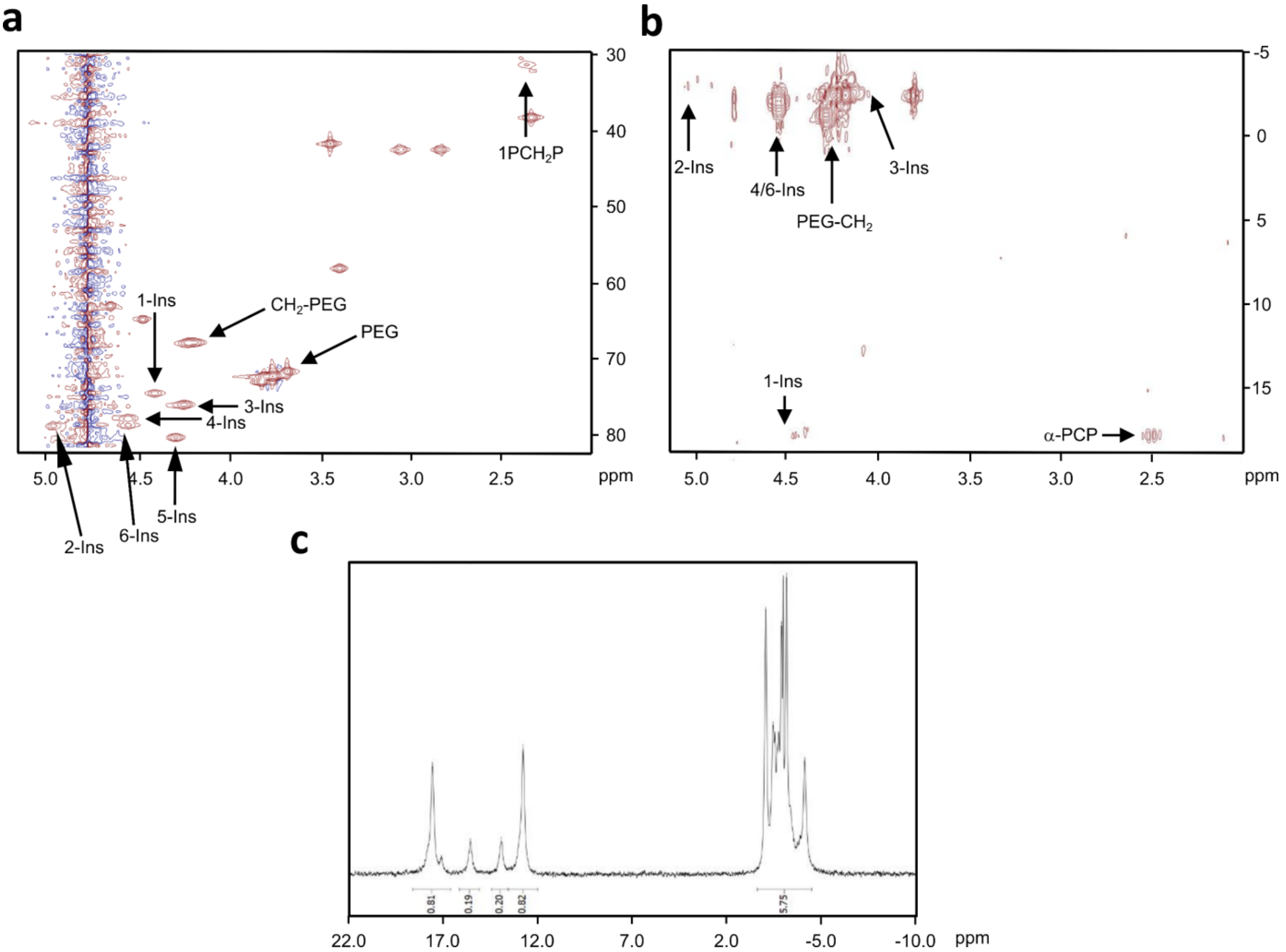

Supplementary Figure 2: 2D-NMR experiments confirming the correct attachment of linker at the 3- or 5-position and PCP group at 1-position for biotin-3/5L-1PCP-InsP$_5$ (1a and 1b).

a: ^{1}H-^{13}C-DEPT-CLIP-COSY NMR experiment. The X-axis shows the ^{1}H-dimension and the y-axis displays the ^{13}C-dimension. C-H correlation of the *myo*-inositol scaffold as well as other interesting correlations like PCH$_2$P, PEG, and CH$_2$-PEG are assigned by black arrows.

b: ^{31}P-HMBC NMR experiment. The X-axis shows the ^{1}H-dimension and the y-axis displays the ^{31}P-dimension. Correlation for the *myo*-inositol scaffold as well as CH$_2$-PEG and a-PCH$_2$P correlation are highlighted with black arrows. b-PCH$_2$P correlation is not visible due to low material availability and can only be detected in the 1D-^{31}P NMR. **C**: ^{31}P-NMR experiment. The a- and b-phosphates of the 1PCP group show a ratio of 20% 5L-compound and 80% 3L-compound. Together A, B, and C confirm the attachment of linker amidate at 3-OH or 5-OH and PCP-amidate at 1-OH.

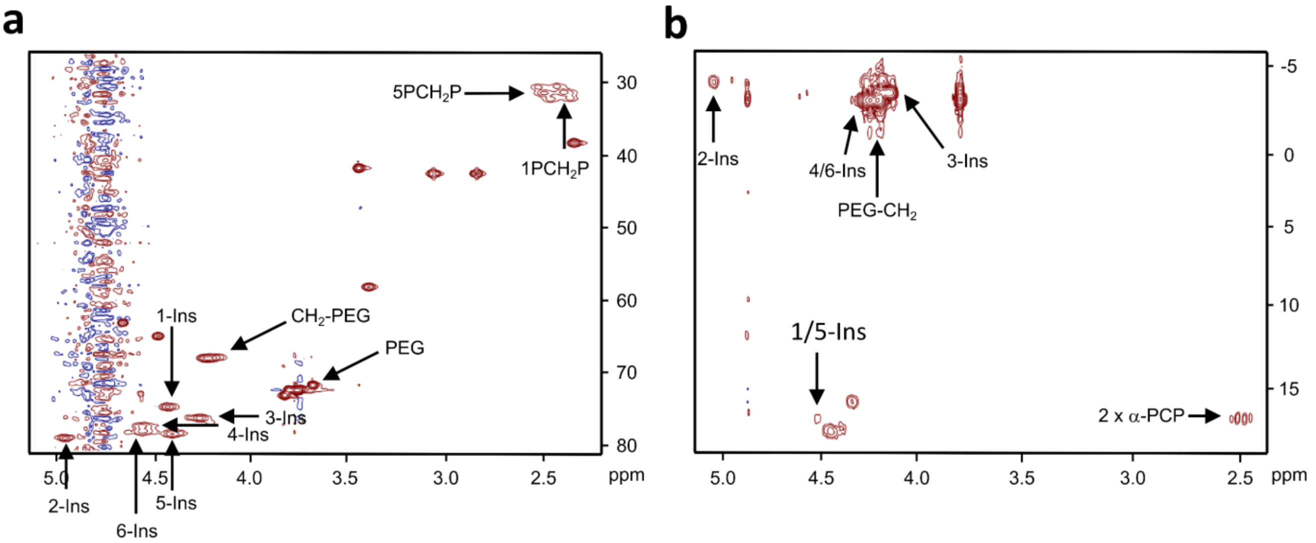

Supplementary Figure 3: 2D-NMR experiments confirming the correct attachment of linker at the 3-position and PCP groups at 1-, and 5-position for biotin-3L-1,5(PCP)$_2$-InsP$_4$ (2).

a: ^{1}H-^{13}C-DEPT-CLIP-COSY NMR experiment. The X-axis shows the ^{1}H-dimension and the y-axis displays the ^{13}C-dimension. C-H correlation of the *myo*-inositol scaffold as well as other interesting correlations like PCH$_2$P, PEG, and CH$_2$-PEG are assigned by black arrows.

b: ^{31}P-HMBC NMR experiment. The X-axis shows the ^{1}H-dimension and the y-axis displays the ^{31}P-dimension. Correlation for the myo-inositol scaffold as well as CH$_2$-PEG and a-PCH$_2$P correlation are highlighted with black arrows. b-PCH$_2$P correlation is not visible due to low material availability and can only be detected in the 1D-^{31}P NMR. Together A and B confirm the attachment of linker amidate at 3-OH and PCP-amidate at 1- and 5-OH.

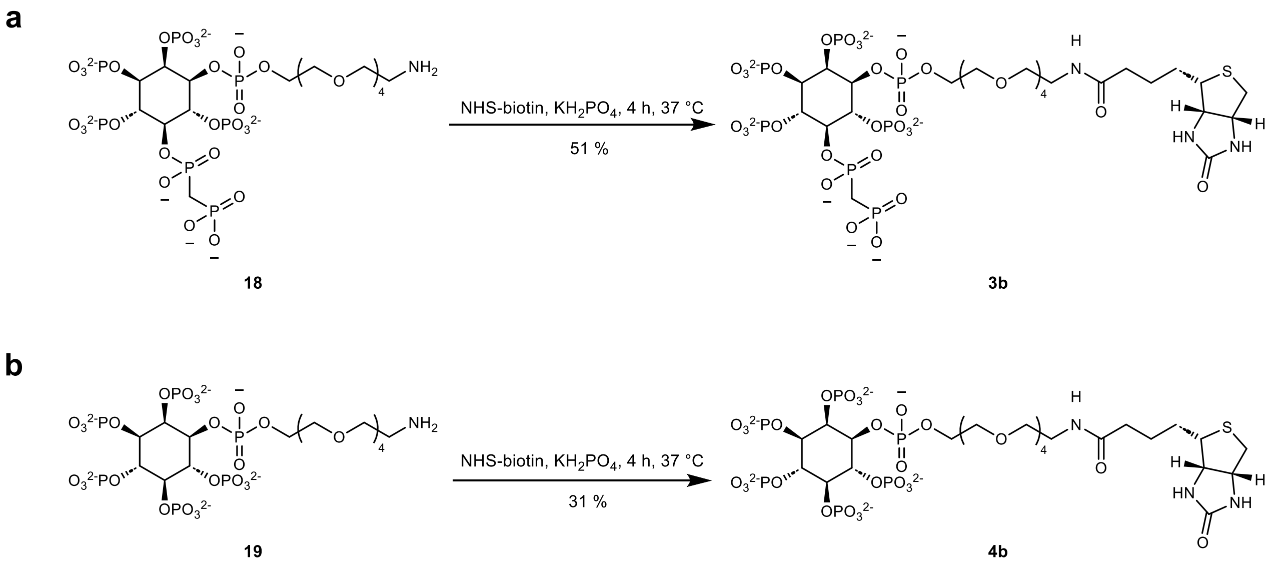

Supplementary Figure 4: Synthesis of biotin-InsP$_6$ and biotin-5PCP-InsP$_5$ with the linker attachment either at position 1 or 3.

For simplicity, only the 1-linked enantiomer is shown. **18** and **19** were obtained according to a reported procedure[1].

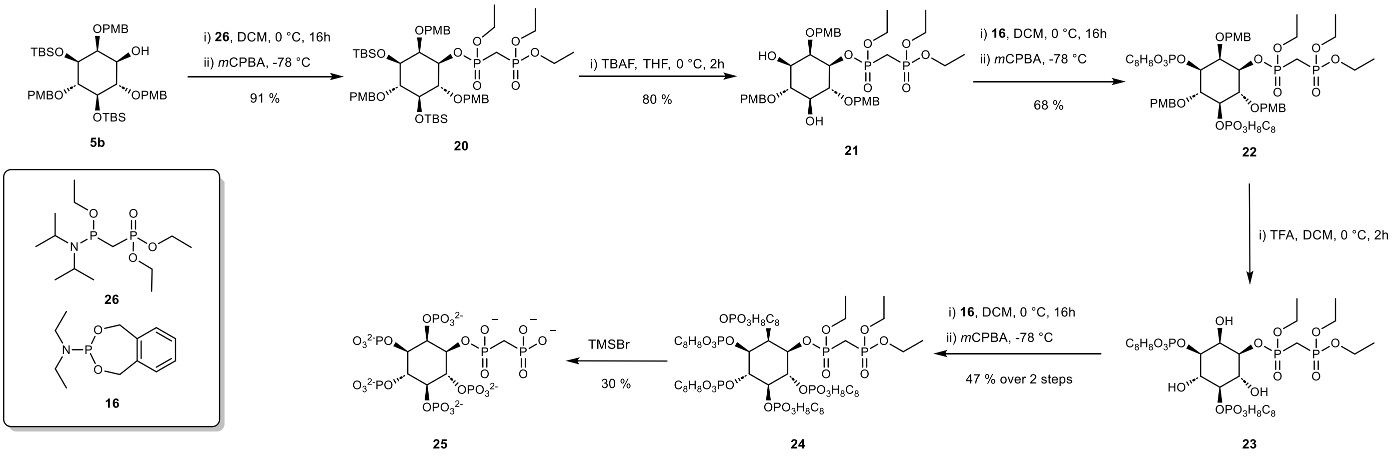

Supplementary Figure 5: Synthesis of 1PCP-InsP$_5$ starting with pure enantiomer 5a. Phosphoramidite **26** was synthesized following a published procedure[2].

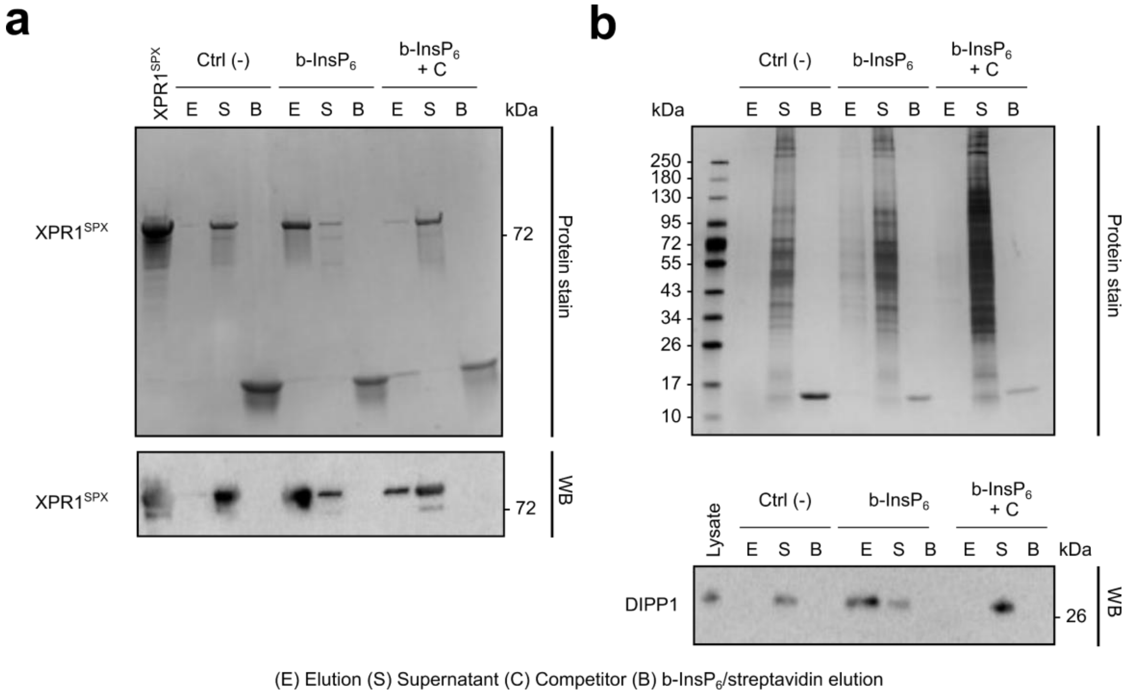

Supplementary Figure 6. Pull-down validation experiments

a: Pulldown capture and elution efficiency between XPR1^{SPX} and b-InsP$_6$.Recombinant XPR1^{SPX} (5 µM) was incubated for 1 h with 45 nmol biotin-InsP$_6$ immobilized on streptavidin–sepharose beads. For the competition assay, the protein was preincubated with 500 µM InsP$_6$ (C) prior to pulldown. After incubation, the supernatant was collected, beads were washed, and bound proteins were competitively eluted with the corresponding endogenous ligand InsP$_6$ (10 mM). Streptavidin-bound b-InsP$_6$ was released under chemical (8M urea)/thermal denaturing (10 min @ 95°C) conditions. Supernatants and eluates were separated by SDS–PAGE, visualized by protein staining, and analyzed by western blotting using an anti-His antibody. A no-reagent condition was included as a negative control (Ctrl). Data are from a single experiment (n = 1). Source data are provided as a Source Data file.

b: Pulldown capture and elution efficiency between HEK293T cell lysates and b-InsP$_6$.HEK293T lysates (1.5 mg) were incubated for 1 h with 45 nmol biotin-InsP$_6$ immobilized on streptavidin–sepharose beads. For the competition assay, the lysates were preincubated with 500 µM InsP$_6$ (C) prior to pulldown. After incubation, the supernatant was collected, beads were washed, and bound proteins were competitively eluted with the corresponding endogenous ligand (10 mM). Streptavidin-bound b-InsP$_6$ was released under chemical/thermal denaturing conditions as previously described. Supernatants and eluates were separated by SDS–PAGE, visualized by protein silver staining, and analyzed by western blotting using anti DIPP1. A no-reagent condition was included as a negative control (Ctrl (-)). Data are from a single experiment (n = 1). Source data are provided as a Source Data file.

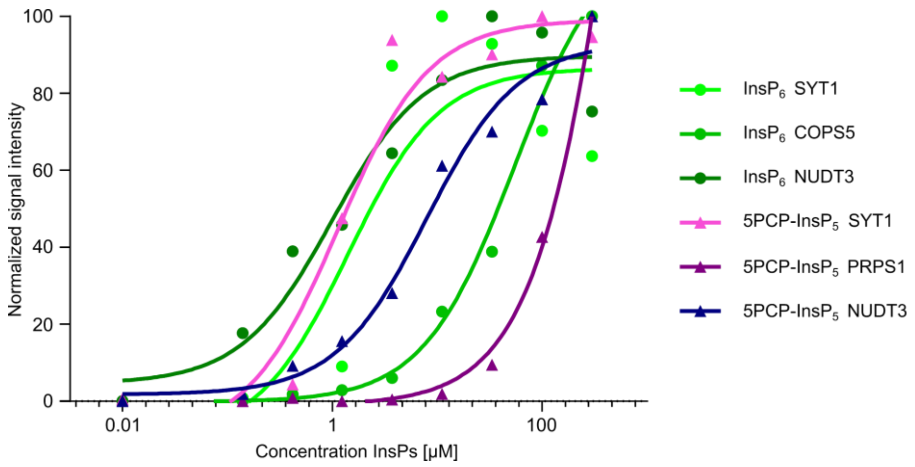

Supplementary Figure 7: Concentration-dependent affinity enrichment and western blot analysis. The obtained signals from Figure 3c were quantified and plotted against the biotin-$InsP_6$ or biotin-5PCP-$InsP_5$ concentration. Source data are provided as a Source Data file.

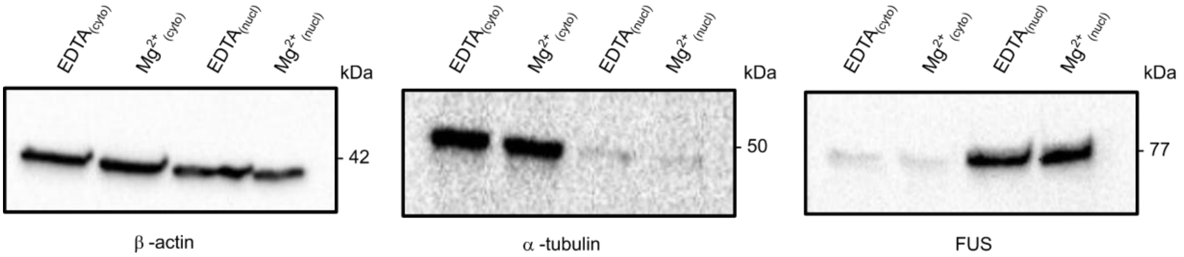

Supplementary Figure 8: Cell lysis and separation of cytosolic and nuclear fraction.
Western blot analysis using cytosolic markers b-actin and a-tubulin and nuclear markers b-actin and FUS. Data are from a single experiment (n = 1). Source data are provided as a Source Data file.

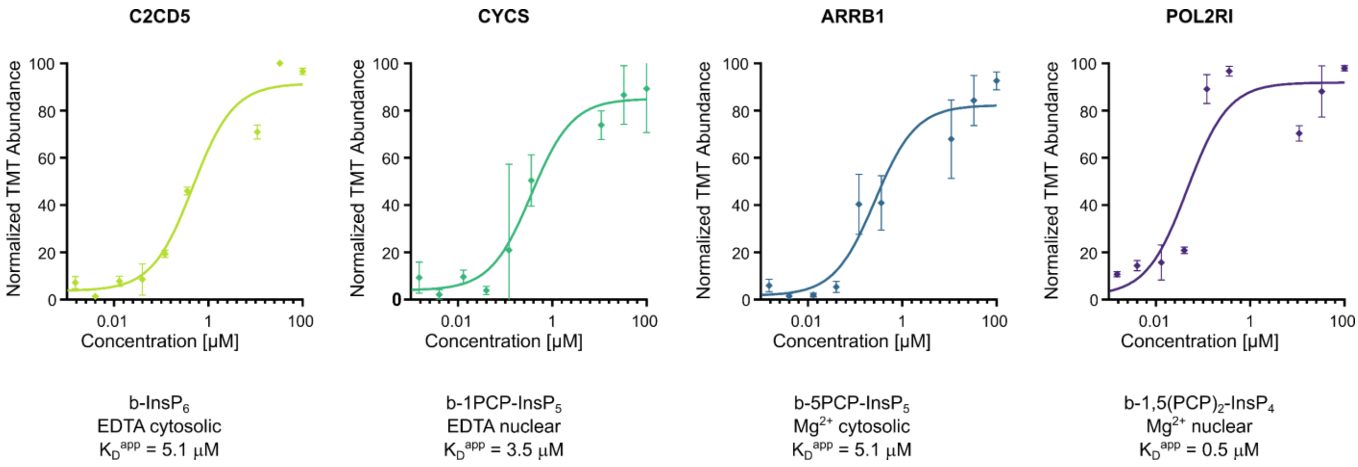

Supplementary Figure 9: K_D^{app} binding curves for the (PP)-InsP binding proteins C2CD5, CYCS, ARRB1, and POLR2I (from left to right). Every data point represents the mean of at least 2 out of 3 biological replicates and the error bars depict the SEM. Source data are provided as a Source Data file.

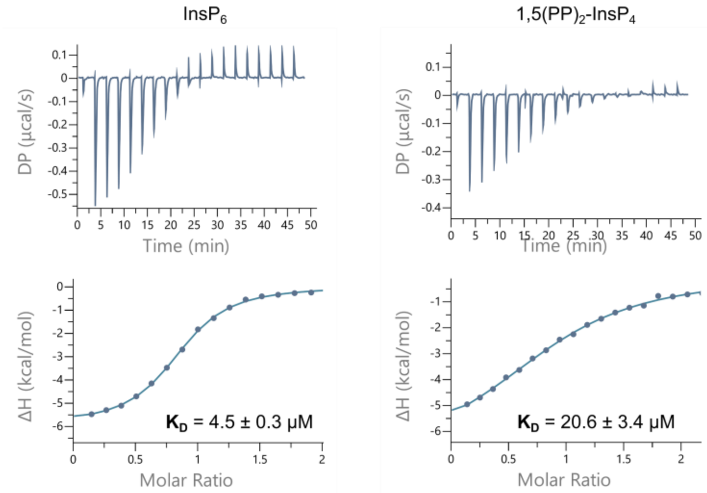

Supplementary Figure 10 - Biophysical validation of PMVK as a ligand-binding protein was performed using ITC assays against endogenous $InsP_6$. and $1,5(PP)_2$-$InsP_4$. All measurements were performed in triplicate.

Supplementary Figure 11 - Clustered heatmap relative quantification

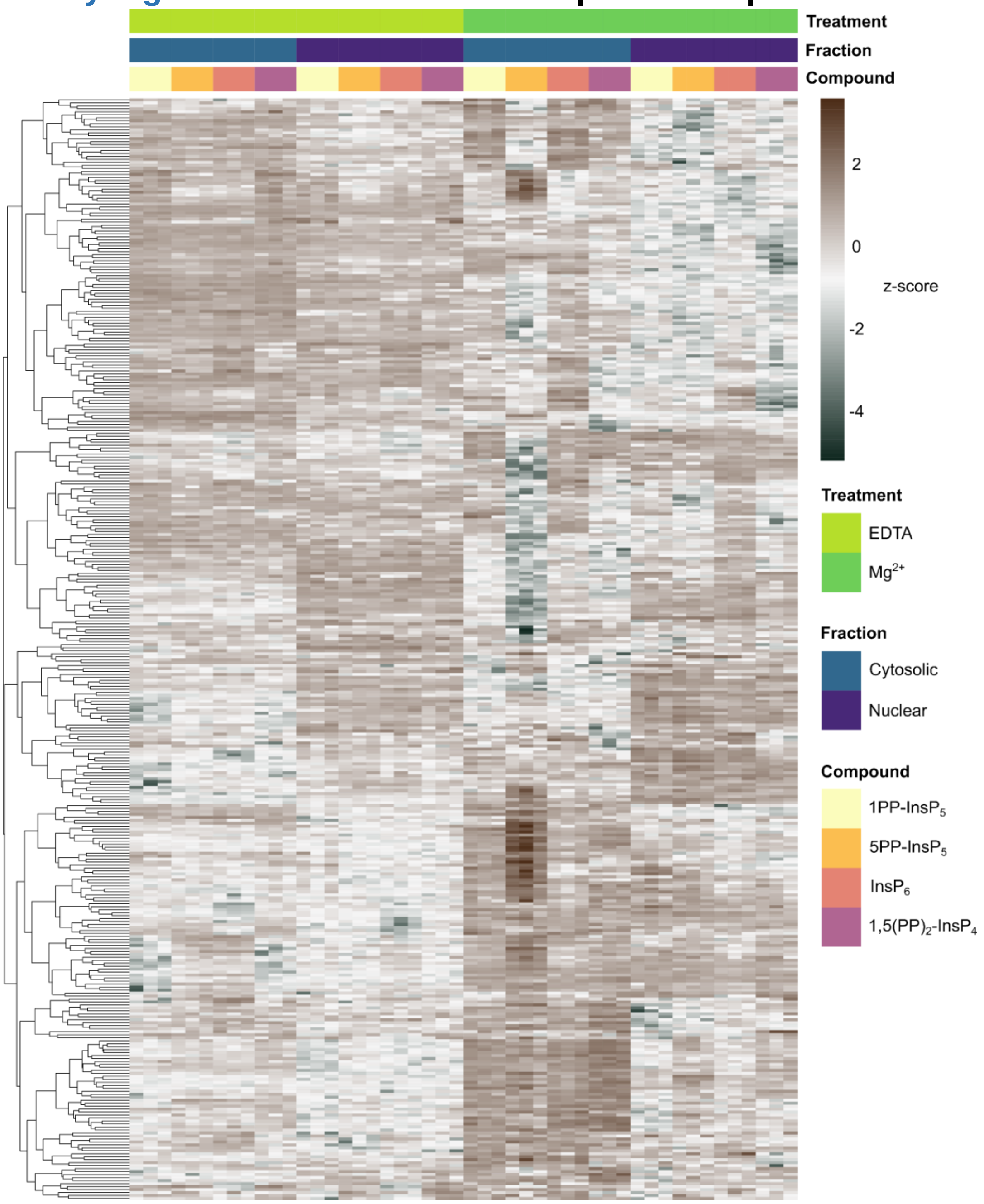

Supplementary Figure 11. Hierarchical clustering of proteins significantly enriched by the affinity reagents across all conditions (EDTA/Mg^{2+} and cytosolic/nuclear). Proteins were quantified by TMT-based multiplexed proteomics, using a single TMT channel (33.3 µM) compound that was shared across all biological replicates and experimental conditions to enable direct quantitative comparison. Only proteins quantified in at least two out of three biological replicates in at least one condition were retained for analysis. Reporter ion intensities were $\log_2$-transformed, median-normalized across samples, and missing values were imputed using a minimal probability (MinProb) approach to model signals close to the detection limit. Protein abundances were row-wise z-scored to emphasize relative changes across conditions. Statistical significance was assessed using a three-way ANOVA testing the effects of compound, cellular fraction (cytosolic vs nuclear), and treatment (Mg^{2+} vs EDTA), followed by Benjamini–Hochberg false discovery rate (FDR) correction. Proteins with a significant compound effect (FDR < 0.05) and no significant contribution from fraction or treatment were retained, yielding 370 compound-responsive proteins. Rows are clustered based on z-scored abundance profiles, while columns are displayed in a fixed experimental order.

a

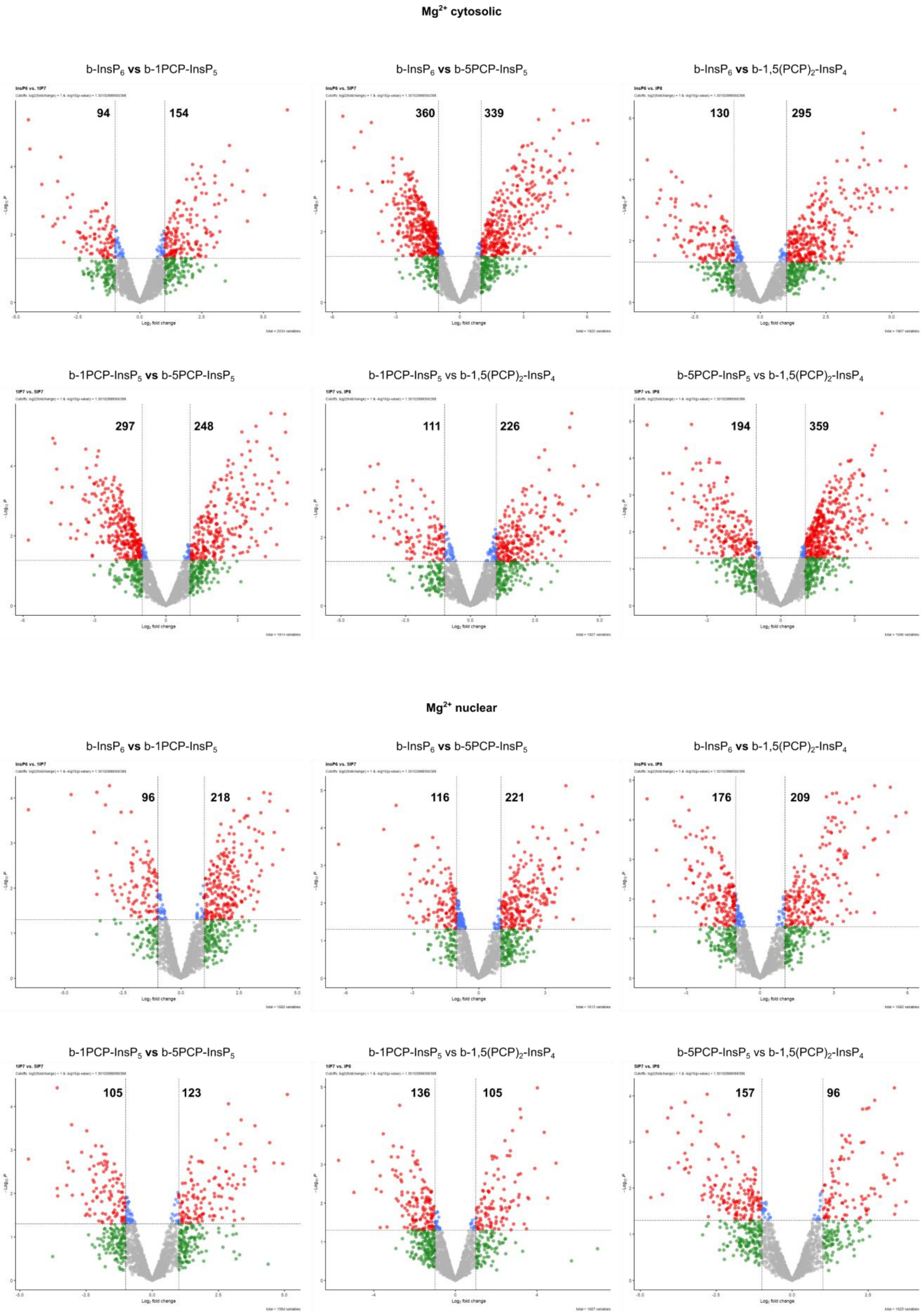
Mg2+ cytosolic
b-InsP6 vs b-1PCP-InsP5
94
154
b-InsP6 vs b-5PCP-InsP5
360
339
b-InsP6 vs b-1,5(PCP)2-InsP4
130
295
b-1PCP-InsP5 vs b-5PCP-InsP5
297
248
b-1PCP-InsP5 vs b-1,5(PCP)2-InsP4
111
226
b-5PCP-InsP5 vs b-1,5(PCP)2-InsP4
194
359
Mg2+ nuclear
b-InsP6 vs b-1PCP-InsP5
96
218
b-InsP6 vs b-5PCP-InsP5
116
221
b-InsP6 vs b-1,5(PCP)2-InsP4
176
209
b-1PCP-InsP5 vs b-5PCP-InsP5
105
123
b-1PCP-InsP5 vs b-1,5(PCP)2-InsP4
136
105
b-5PCP-InsP5 vs b-1,5(PCP)2-InsP4
157
96

b

EDTA cytosolic

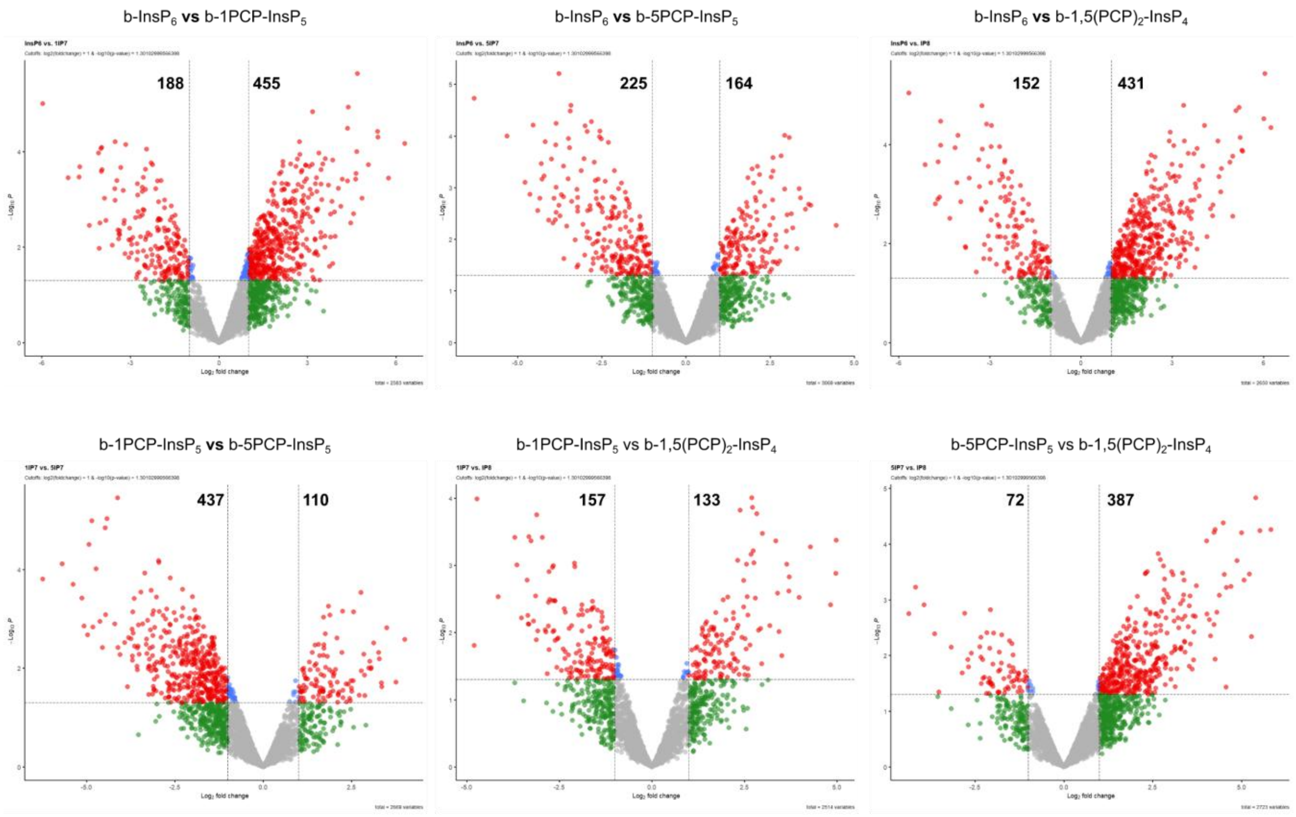

EDTA nuclear

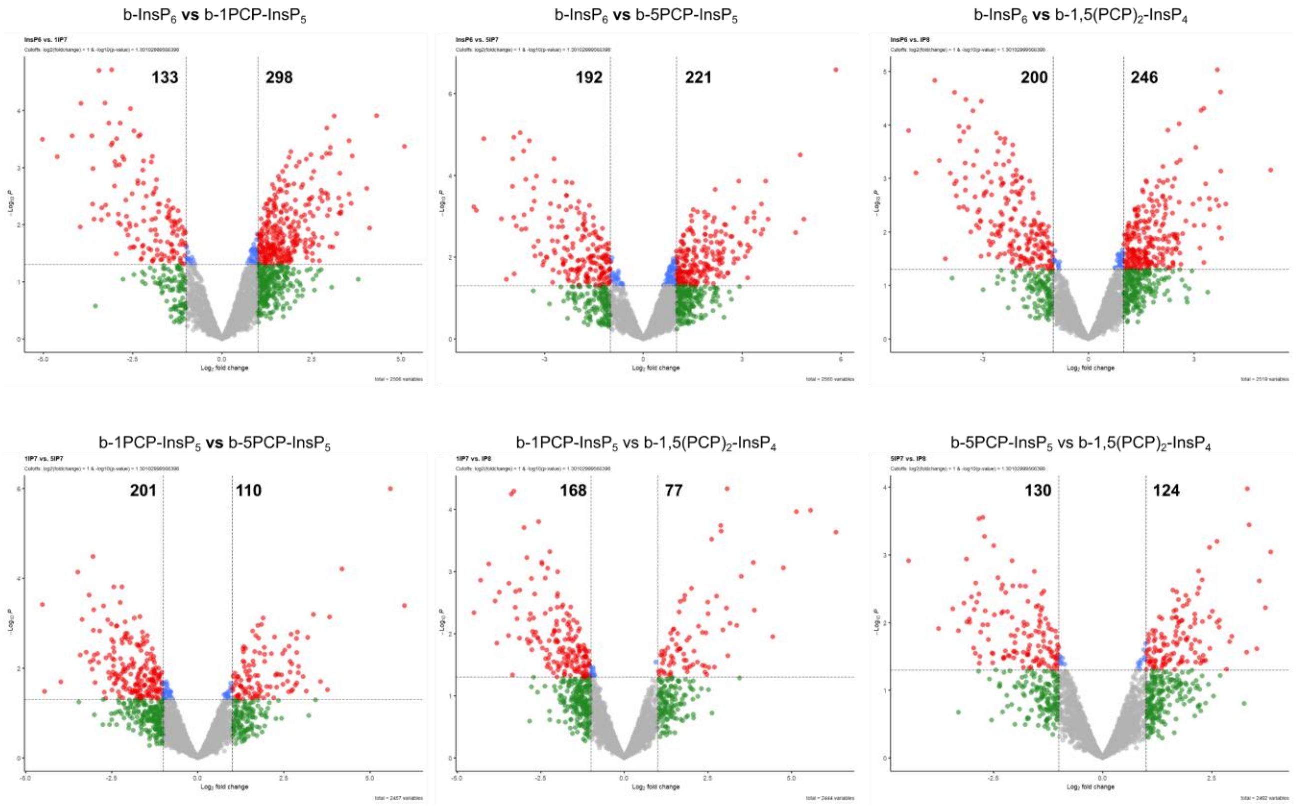

Supplementary Figure 12. Relative quantification comparison between probes. The labeling specificity of affinity reagents b-$InsP_6$, b-1PCP-$InsP_5$, b-5PCP-$InsP_5$, and b-1,5$(PCP)_2$-$InsP_4$ (33.3 µM) in HEK293T nuclear and cytosolic fractions under **a**: Mg^{2+} or **b**: EDTA conditions was assessed. A single channel from the TMT-based quantitative proteomics assays was used to compare baits against each other. Only proteins detected in at least 2 replicates were retained for analysis. Statistical significance was assessed using a cutoff: $p \leq 0.05$ (Bayes moderated t-test). Volcano plots display $\log_2$ fold enrichment on the x-axis and $-\log_{10}$ *p*-value on the y-axis, with significance thresholds set at $|\log_2(\text{fold change})| > 1$ and $-\log_{10}(p) > 1.3$. Proteins significantly enriched by the respective probes are shown as red-filled circles. Green circles indicate proteins with $|\log_2(\text{fold change})| > 1$ but $-\log_{10}(p)$ below the threshold, while blue circles represent proteins with $-\log_{10}(p) > 1.3$ but $|\log_2(\text{fold change})| < 1$. Enriched targets are listed in Supplementary Data 2.

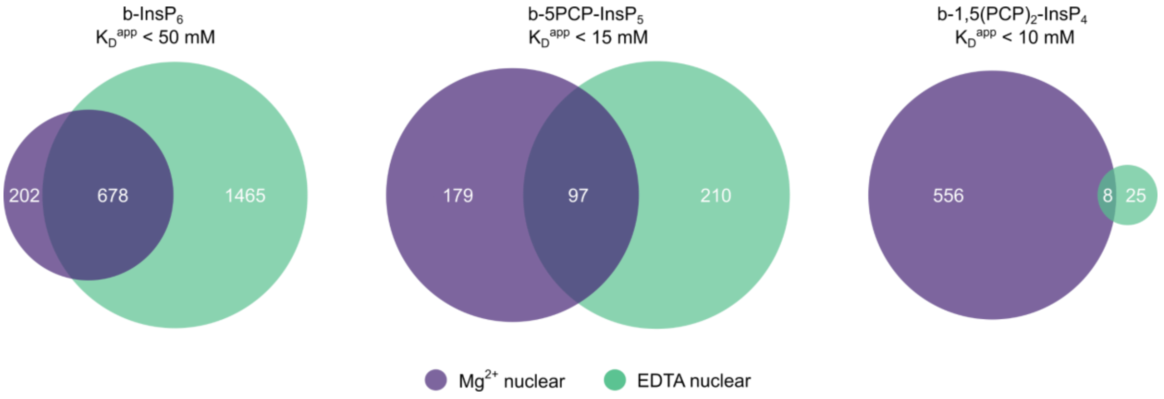

Supplementary Figure 13: Venn diagrams[3] of all proteins quantified for biotin-$InsP_6$ (K_D^{app} = 0 – 50 µM), biotin-5PCP-$InsP_5$ (K_D^{app} = 0-15 µM), and biotin-1,5$(PCP)_2$-$InsP_4$ (K_D^{app} = 0 – 10 µM) in the Mg^{2+} (purple)/EDTA (green) nuclear fractions.

Supplementary Table 1: Table of reagents and resources

Reagent or Resource	Source	Identifier
Antibodies		
INPP5K	Proteintech	Cat# 15098-1-AP
COPS5/JAB1	Proteintech	Cat# 27511-1-AP
SYT1	Proteintech	Cat# CL488-68043
PRPS1	Proteintech	Cat# 15549-1-AP
NUDT3(DIPP1)	Proteintech	Cat# 20542-1-AP
NAMPT/PBEF	Proteintech	Cat# 66385-1-IG
HRP-conjugated 6*His	Proteintech	Cat# HRP-66005
STK24(MST3)	Thermo Fisher Scientific	Cat# PA5-120097
FAK (PTK2)	Thermo Fisher Scientific	Cat# PA5-17591
PMVK	Absea	Cat# P02302PA
Polyclonal rabbit HRP	Cell Signalling Technology	Cat# 7074; RRID: AB_2099233
Polyclonal mouse HRP	Cell Signalling Technology	Cat#7076; RRID: AB_330924
FUS	Santa Cruz	Cat# sc-47711
b-actin	Santa Cruz	Cat# sc-47778
a-tubulin	Santa Cruz	Cat# sc-8035
Chemicals, Peptides and Recombinant Proteins		
Dulbecco's Modified Eagle Medium (DMEM)	Gibco	Cat# 11960-044
Penicillin/streptomycin	Gibco	Cat# 15140-122
L-Glutamine	Gibco	Cat# 25030-024
DPBS	Gibco	Cat# 14190-094
Pierce IP lysis buffer	Thermo Scientific	Cat# 87787
PhosSTOP Phosphatase inhibitor	Sigma-Aldrich	Cat# 4906845001
cOmplete, Mini, EDTA-free Protease Inhibitor Cocktail	Sigma-Aldrich	Cat# 11836170001
Affi-Gel 15 Gel	Bio-Rad	Cat# 1536051
Triethylammonium bicarbonate buffer	Sigma-Aldrich	Cat# T7408
Trypsin	Serva	Cat# 5763_37286
Lys C	Fujifilm	Cat# 121-05063
CAA	Sigma-Aldrich	C0267-100g, CAS: 79-07-2
TCEP	Sigma-Aldrich	Art. No.; 646547-1ML, CAS: 51805-45-9
biotin-$InsP_6$	This study	N/A
biotin-5PCP-$InsP_5$	This study	N/A
biotin-1PCP-$InsP_5$	This study	N/A
biotin-1,5$(PCP)_2$-$InsP_4$	This study	N/A
1/3***L***-$InsP_6$	Furkert *et al.* 2020[1]	N/A
1/3***L***-5PCP-$InsP_5$	Furkert *et al.* 2020[1]	N/A
$InsP_6$	SciChem	Cat# 6-0-123456-Na
5PCP-$InsP_5$	Wu *et al.* 2013	N/A
5PP-$InsP_5$	Puschmann *et al.* 2019[4]	N/A
1PCP-$InsP_5$	Wu *et al.* 2014[5]	N/A
1,5$(PCP)_2$-$InsP_4$	Hostachy *et al.* 2021[6]	N/A
1,5$(PP)_2$-$InsP_4$	Puschmann *et al*. 2019[4]	N/A
GST-hDipp1	Wu *et al*. 2015[7]	N/A
MBP-SPX XPR1	Li *et al*. 2020[8]	N/A
SYT1	Wangt *et al*. 2014[9]	N/A
5-phenyl-1H-tetrazole	VWR	B25664; CAS: 18039-42-4
p-Toluenesulfonic acid monohydrate	Sigma Aldrich	416665; CAS: 75-91-2

3-Chloroperbenzoic acid	Sigma Aldrich	273031; CAS: 937-14-4
o-Xylylene N,N-diethylphosphoramidite	Sigma Aldrich	393835; CAS: 82372-35-8
1H-tetrazole	Sigma Aldrich	88158; CAS: 288-94-8
4,5-Dicyanimidazol	Sigma Aldrich	324132; CAS: 1122-28-7
Tetrabutylammoniumfluorid Trihydrat	Sigma Aldrich	86872; CAS: 87749-50-6
Trimethylsilyl bromide	Sigma Aldrich	194409; CAS: 2857-97-8
Streptavidin Sepharose	GE Healthcare Life Sciences	Cat#17-5113-01
Medronic acid	Sigma Aldrich	Cat# 64255-1G-F
TNIK (1-367)	Biomol	Cat# BPS-11708
Bovine myelin basic protein	Sigma Aldrich	Cat# M1891-5MG
Critical Commercial Assays		
TMT 10plex™	Thermo Fisher Scientific	Cat# 90111
TMT11-131C label reagent	Thermo Fisher Scientific	Cat# A37724
Pierce BCA-Protein Assay	Thermo Fisher Scientific	Cat# 24612
Pierce Silver Stain kit	Thermo Fisher Scientific	Cat# 23227
TNIK kinase enzyme system	Promega	Cat# V4158
Kinase-Glo Plus	Promega	Cat# V3771
Instant coomassie	Abcam	Cat# ab119211
SuperSignal™ West Femto Maximum Sensitivity Substrate	Thermo Fisher Scientific	Cat# 34095
Deposited Data		
HEK293T raw and analyzed data	This study	
Experimental Models: Cell lines		
HEK293T	ATCC	CRL-3216
Software and Algorithms		
Proteome Discoverer 3.0	Thermo Fisher Scientific	
BioRender	N/A	https://www.biorender.com/
MestReNova v.10.0.2	N/A	https://mestrelab.com/
RStudio 2025.09.2	Posit Software, PBC	https://www.r-studio.com/de/
TOPSPIN v3.5	N/A	https://www.bruker.com/en/products-and-solutions/mr/nmr-software/topspin
Scansite4.0		https://scansite4.mit.edu/#home
IUPred3		https://iupred3.elte.hu/
DeepVenn		https://www.deepvenn.com/
Prism 5.04		https://www.graphpad.com
Image Lab 6.1.0	Bio-Rad	https://www.bio-rad.com/de-de/product/image-lab-software
ChemDraw 22.0.3300	Perkin Elmer	https://www.additive-net.de/de/software/produkte/perkinelmer/chemdraw/neu#version-18
Inkscape 1.4.2		https://inkscape.org/de/
Orbitrap Fusion Lumos Tribrid Mass Spectrometer	Thermo Fisher Scientific	
FAIMS Pro Interface	Thermo Fisher Scientific	Cat# FMS02-10001
Orbitrap Fusion Tribrid Mass spectrometer	Thermo Fisher Scientific	
Loading column 0.075 x 70 mm	Thermo Fisher Scientific	Cat# 164946

C18 column: Poroshell 120-EC-C18, 2.7 µm (inhouse packed)	Agilent	
Dionex Ultimate 3000 HPLC	Thermo Fisher Scientific	
PlatePrep 96-well vacuum distributor	Sigma Aldrich	Cat# 57192-U
Multiscreen® 96 well Plate, hydrophilic PVDF membrane	Sigma Aldrich	Cat# MSBV1210

Materials availability

Requests for unique/stable reagents should be directed to Dorothea Fiedler (fiedler@fmp-berlin.de). Availability may be limited due to multistep synthesis.

Supplementary Methods

CHEMICAL SYNTHESIS

General information

All chemicals were purchased from the commercial suppliers VWR, Sigma Aldrich, Carl Roth, TCI, Thermo Scientific, and Roche and used without further purification. Solvents were purchased from Fisher Chemicals and dried over a 3 Å molecular sieve or dried in an MBraun-SPS-5 solvent purification system.

Silica-based flash chromatography was performed using the Combiflash Rf+™ Teledyne Isco and Redi Sep Rf disposable columns. The crude material was applied on Telos NM support from Kinesis Scientific Expert. Detection was performed at either 254 nm and 280 nm for all hexane/ethyl acetate (EtOAc)-based separation of 220 nm and 254 nm for DCM/MeOH-based separation. Silica-based purification for phosphoramidites was done using high-purity grade silica (Davisil Grade 633, pore size 60 Å, 200-425 mesh particle size) from Sigma Aldrich. Thin layer chromatography was performed using silica gel F254 plates and visualized at 254 nm or stained by potassium permanganate and heating to approximately 200 °C.

Preparative HPLC was performed using a 1260 Agilent Infinity II detector, pump, and autosampler, and a 1290 Agilent Infinity II fraction collector.
HPLC Method 1: YMC Actus Triart C18 (15 x 200 mm) column, solvent: MilliQ + 0.1 % TFA (A), acetonitrile + 0.1 % TFA (B), 35 mL/min. Gradient: 62 % B for 1 minute, followed by a gradient to 68 % B for 6 minutes and a wash at 95 % B for 2 minutes. UV detection was carried out at 220 nm.
HPLC Method 2: YMC Actus Triart C18 (15 x 200 mm) column, solvent: MilliQ + 0.1 % TFA (A), acetonitrile + 0.1 % TFA (B), 35 mL/min. Gradient: 10 % B for 3 minutes, followed by a gradient to 30 % B for 3 minutes and a wash at 95 % B for 2 minutes.
Reactions were monitored on an Agilent Infinity 1260 LC system connected to an Agilent 6130 Quadrupole. A ZOBRAX Rapid Resolution HT Narrow Bore SB-C18 column (2.1 x 50 mm) was used at 30 °C. Water with 0.1 % FA in water (A) and acetonitrile with 0.1 % FA in water (B) were used as mobile phase at a 0.7 mL/min flow rate. Gradients were chosen dependent on the polarity of the molecules either at 10 %-60 % B, 10 %-90 % B, or 40-90 % B. High resolution (HR)-MS was measured on a Thermo Fisher Q-Exactive with direct injection in pESI-FullMS positive or nESI-FullMS negative ion mode.

NMR spectra of ^{1}H, ^{13}C, and ^{31}P were recorded on a Bruker AV-600 (600 MHz) instrument at 277 K using deuterated solvents (D_2O, $CDCl_3$; CD_3CN) from Deutero. $CDCl_3$ was neutralized and stored over K_2CO_3 before use. Chemical shifts are depicted in ppm. For NMR-based quantification purposes, tetramethylphosphonium bromide solution in D_2O of a known concentration was added and quantitative ^{31}P-NMR was recorded (NMR Method 1). For proton quantification, 3-(trimethylsilyl)-propionic acid-d_4 was dissolved in D_2O and added to the compound solution and ^{1}H-NMR was recorded (NMR method 1). MestreNova 10.0.2. was used for NMR data analysis.

Synthetic precursors

The following precursors were prepared following published procedures. **16** is commercially available. **15** was prepared according to Hostachy *et al*[2], **17** was decribed by Capolicchio *et al*.[10], **S3** was prepared following the procedure by Hager *et al*.[11], **18** was first described by Furkert *et al*[1]., and **4** was prepared using a procedure from Couto *et al*[12].

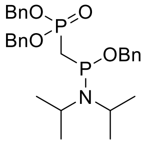

15

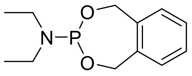

16

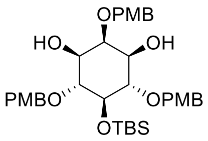

S3

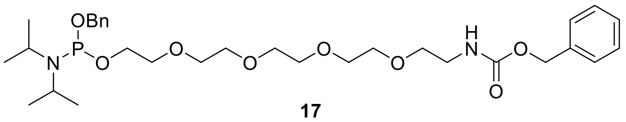

17

$R_1 = PO_3^{2-}$, R_2 = L-NH$_2$
or
R_1 = L-NH$_2$, $R_2 = PO_3^{2-}$

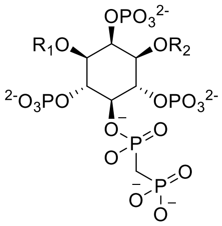

18

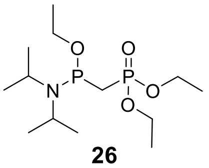

26

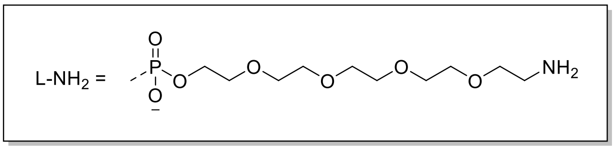

$R_1 = PO_3^{2-}$, R_2 = L-biotin
or
R_1 = L-biotin, $R_2 = PO_3^{2-}$

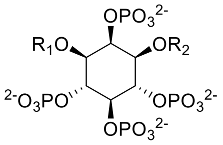

4

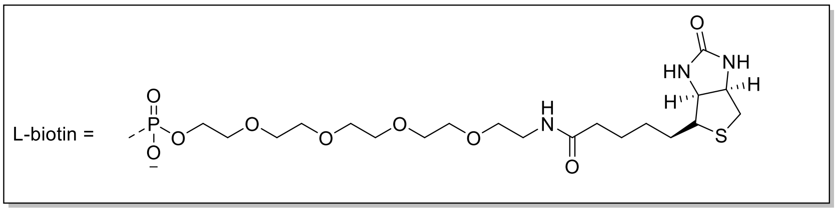

Chemical synthesis

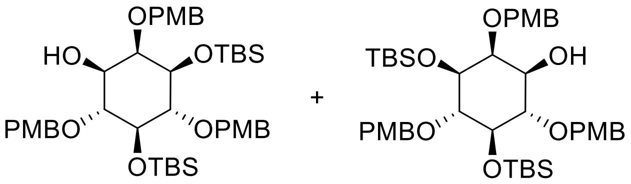

5a **5b**

tert-Butyldimethylsilyl chloride (137 mg, 915 µmol, 1.2 eq.) was added to a solution of inositol **S3** (500 mg, 763 µmol, 1 eq.), and imidazole (124 mg, 1.83 mmol, 2.4 eq.) in DMF (30 mL) and stirred at 60 °C overnight. Progress was followed via LC-MS. The solution was allowed to reach room temperature before it was diluted with EtOAc (300 mL) and washed twice with LiCl (10%, 200 mL) and brine (200 mL). The organic layer was filtered through a water-repellant filter and concentrated under reduced pressure and purified by preparative HPLC (Luna 5u C8 (21.2 x 250 mm) column, solvent: MilliQ+ 0.1 % TFA (A), acetonitrile +0.1 % TFA (B), 35 mL/min. Solvent: 100% B for 5 minutes.) followed by separation of enantiomers on a (S,S) WHELK-O 1 10/100 Kromasil column (25 cm x 21.1 mm) (2.5% *i*-PrOH in *n*-hexane) to give the title compound (242 mg, 41%, 79% with reisolation of starting material (170 mg)) as a viscous oil.

t_R (Enantiomer 1(**5a**)): 18.2 min

t_R (Enantiomer 2(**5b**)): 26.9 min

^{1}H NMR (600 MHz, $CDCl_3$) [ppm] δ = 7.28 (m, 4H), 7.22 (m, 2H), 7, 6.94 – 6.89 (m, 2H), 6.90 – 6.84 (m, 2H), 6.85 – 6.82 (m, 2H), 4.88 (dd, *J* = 16.3, 11.3 Hz, 2H), 4.73 (t, *J* = 11.9 Hz, 2H), 4.65 (d, *J* = 11.2 Hz, 1H), 4.57 (d, *J* = 10.9 Hz, 1H). 4.12 (q, *J* = 7.2 Hz, 2H),
3.83 (s, 3H), 3.81 (s, 3H), 3.80 (s, 3H) 3.80 (m, 1H), 3.68 – 3.61 (m, 2H), 3.61 – 3.57 (m, 1H), 3.53 – 3.46 (m, 2H), 0.85 (s, 18H), 0.10 (s,3H), 0.05 (s,3H), -0.01 (s,3H), -0.09 (s,3H).
^{13}C NMR (151 MHz, $CDCl_3$) [ppm] δ = 171.11, 159.20, 159.16, 158.25, 131.42, 131.27, 131.05, 129.68, 129.23, 127.57, 113.81, 113.77, 113.15, 82.31, 81.85, 80.86, 75.24, 74.84, 74.81, 74.48, 72.33, 60.38, 55.29, 55.24, 55.16, 26.08, 25.95, 18.05, 18.00, 14.20, -3.93, -4.00, -4.25, -4.76.
Calculated $[M+Na]^+$: 791.3982; Measured: 791.3964.

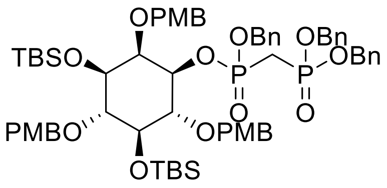

6

Enantiomer **5b** (68 mg, 88.4 µmol, 1 eq.) and phosphoramidite **15** (90.8 mg, 176 µmol, 2.0 eq.) were coevaporated twice with dry ACN and dried under high vacuum for 2 h. Dry CH_2Cl_2 (2 mL) was added and the reaction mixture was cooled in an ice bath. 5-phenyl-1H-tetrazole (90.8 mg, 176 µmol, 2.0 eq.) was added as a solid. After 5-phenyl-1*H*-tetrazole was dissolved, the ice bath was removed and the mixture was additionally stirred for 16 h. The solution was cooled in a $CO_{2(s)}$/acetone bath and *m*CPBA (77%, 48.8 mg, 217 µmol, 2.5 eq.) was added carefully: the mixture was stirred for 30 min in a $CO_{2(s)}$/acetone bath and 2 h at room temperature. EtOAc (120 mL) was added and the organic phase was washed with aq. $Na_2S_2O_3$ (100 mL), sat. aq. $NaHCO_3$ (100 mL) and brine (100 mL). The organic layer was filtered through a water-repellant filter, concentrated under reduced pressure, and purified by flash chromatography (0% to 4% MeOH in CH_2Cl_2) to give the title compound (51 mg, 42.6 µmol, 49%) as a colorless oil and a mixture of two diastereomers.

^{1}H NMR (600 MHz, CD_3CN) [ppm] δ = 7.44 – 7.14 (m, 21H), 6.86 – 6.65 (m, 6H), 5.07 – 4.49 (m, 12H), 3.73 – 3.69 (m, 6H), 3.62 (d, J = 18.2 Hz, 3H), 2.55 – 2.27 (m, 2H), 0.81 (d, J = 5.9 Hz, 6H), 0.78 (s, 3H), 0.76 (d, J = 4.2 Hz, 9H), 0.13 – 0.08 (m, 2H), 0.02 (s, 1H), -0.01 (d, J = 6.2 Hz, 2H), -0.04 (s, 1H), -0.12 (d, J = 13.4 Hz, 3H), -0.17 (d, J = 6.6 Hz, 3H).

^{31}P NMR (243 MHz, CD_3CN) [ppm] δ = 20.36, 19.86, 19.84, 19.73.

^{13}C NMR (151 MHz, CD_3CN) [ppm] δ = 160.18, 159.80, 159.44, 132.31, 131.95, 131.90, 130.23, 130.11, 129.90, 129.77, 129.66, 129.53, 129.48, 129.47, 129.41, 129.29, 129.20, 128.96, 128.90, 128.90, 128.66, 118.26, 114.56, 114.38, 114.23, 114.08, 82.65, 82.60, 81.64, 75.98, 75.94, 75.71, 75.68, 75.44, 75.29, 68.64, 68.58, 68.54, 55.86, 55.77, 55.74, 26.49 (3C), 26.34 (3C), 23.07, 18.54 (2C), -3.47, -3.87, -3.92, -4.53.

Calculated $[M+2H]^{2+}$: 599.2588; Measured: 599.2573.

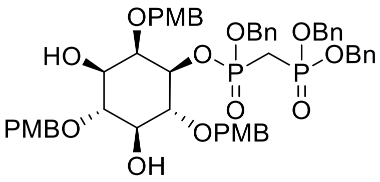

S1

Compound **6** (50 mg, 51.6 µmol, 1 eq.) was dissolved in dry THF (2 mL) and cooled under an ice bath. TBAF in THF (1 M, 53.9 mg, 206 µmol, 0.5 mL, 4 eq.) was added dropwise. The reaction mixture was allowed to reach room temperature and was stirred for 2 h. The reaction mixture was diluted with EtOAc (50 mL) and the organic layer was washed twice with saturated $CaCl_2$ solution (50 mL) and brine (50 mL). The organic layer was filtered through a water-repellant filter, concentrated under reduced pressure, and purified by flash chromatography (0% to 5% MeOH in CH_2Cl_2) to give the title compound (30 mg, 30.9 µmol, 75%) as a colorless oil and a mixture of two diastereomers.

^{1}H NMR (600 MHz, CD_3CN) [ppm] δ = 30 – 7.09 (m, 21H), 6.84 – 6.63 (m, 6H), 5.02 – 4.81 (m, 6H), 4.71 – 4.46 (m, 6H), 3.69 – 3.63 (m, 6H), 3.60 – 3.55 (m, 3H), 2.57 – 2.33 (m, 2H).
^{31}P NMR (243 MHz, CD_3CN) [ppm] δ = 20.55, 20.03, 19.32, 18.63.
^{13}C NMR (151 MHz, CD_3CN) [ppm] δ = 159.23, 159.17, 159.03, 136.48, 136.46, 136.36, 131.35, 131.12, 131.09, 130.96, 130.94, 130.20, 130.17, 130.16, 129.93, 129.90, 129.88, 129.74, 129.15, 129.07, 128.88, 128.85, 128.83, 128.72, 128.46, 128.00, 127.96, 127.78, 127.43, 127.40, 114.12, 114.09, 114.06, 113.06, 113.03, 113.00, 81.65, 80.68, 80.34, 79.35, 74.07, 74.04, 72.12, 71.15, 68.67, 67.62, 66.70, 56.32, 55.36, 54.41, 53.45, 25.32.
Calculated $[M+H]^+$: 969.3375; Measured: 969.3378.

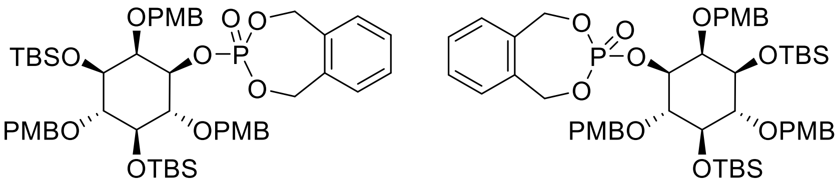

S1a **S1b**

Protected inositol **5a/b** (95 mg, 0.124 mmol, 1 eq.) and phosphoramidite **16** (73 mg, 0.284 mmol, 2.3 eq.) were co-evaporated twice with CH_3CN (2 mL) and then dried under vacuum for 1 h, dissolved in dry CH_2Cl_2 (2 mL) and cooled in an ice bath, before 4,5-dicyanoimidazole (44 mg, 0.371 mmol, 3.0 eq.) was added as a solid. After 4,5-dicyanoimidazole was dissolved, the ice bath was removed and the mixture was additionally stirred for 1 h. The solution was cooled in an ice bath and *m*CPBA (100 mg, 77%; 0.433 mmol, 3.5 eq.) was added carefully: The mixture was stirred for 2.5 h at room temperature. EtOAc (20 mL) was added and the organic phase was washed with aq. $Na_2S_2O_3$ (15 mL), sat. aq. $NaHCO_3$ (10 mL) and sat. aq. NaCl (10 mL). The organic layer was dried over Na_2SO_4, concentrated under reduced pressure and purified by silica-based flash chromatography (0% to 40% EtOAc in hexane) to give the titled compound as a colorless oil (105 mg, 89%).

^{1}H NMR (600 MHz, $CDCl_3$) [ppm] δ = 7.50 – 7.08 (m, 11H), 6.95 – 6.89 (m, 2H), 6.89 – 6.82 (m, 2H), 6.77 – 6.70 (m, 2H), 5.14 (dd, J = 16.9, 13.7 Hz, 1H), 5.05 – 4.90 (m, 5H), 4.83 – 4.68 (m, 4H), 4.42 (ddd, J = 9.8, 6.9, 2.6 Hz, 1H), 3.93 – 3.87 (m, 1H), 3.67 (dd, J = 9.6, 2.3 Hz, 1H), 3.54 (t, J = 8.9 Hz, 1H), 0.87 (s, 8H), 0.84 (s, 8H), 0.15 (s, 3H), 0.03 (s, 3H), -0.00 (s, 3H), -0.08 (s, 3H).

^{13}C NMR (151 MHz, $CDCl_3$) [ppm] δ = 159.07, 158.64, 158.21, 135.35, 135.20, 131.47, 131.39, 130.88, 129.04, 128.92, 128.85, 128.73, 128.71, 128.68, 127.49, 113.63, 113.30, 113.13, 81.49, 80.60, 80.36, 80.31, 79.25, 79.21, 75.49, 75.19, 75.09, 74.63, 73.81, 68.42, 68.38, 68.26, 68.22, 55.28, 55.15, 55.11, 26.03, 25.93, 17.98, -3.87, -4.22, -4.83.

^{31}P NMR (243 MHz, $CDCl_3$) [ppm] δ = -0.43.

Calculated $[M+Na]^+$: 973.4114; Measured: 973.4092.

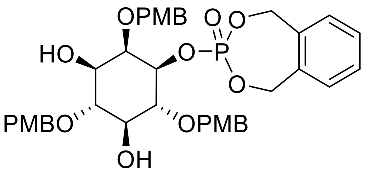

S2a

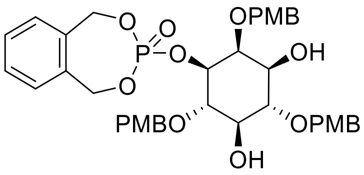

S2b

Compound **S1a/b** (102 mg, 0.107 mmol, 1 eq.) was dissolved in THF (8 mL) and cooled under an ice bath. TBAF 1 M in THF (0.4 mL, 4 eq.) was added dropwise. The reaction mixture was allowed to reach room temperature and was stirred for 2 h. The reaction mixture was diluted with EtOAc (50 mL) organic layer was washed aq. $CaCl_2$ and brine. The organic layer was dried over Na_2SO_4, concentrated under reduced pressure and purified by silica-based flash chromatography (0% to 4% MeOH in CH_2Cl_2) to give the title compound (70 mg, 90%) as a colorless oil.

^{1}H NMR (600 MHz, $CDCl_3$) [ppm] δ = 7.45 – 7.10 (m, 10H), 6.93 – 6.88 (m, 4H), 6.78 – 6.68 (m, 2H), 5.26 – 4.70 (m, 10H), 4.41 – 4.31 (m, 2H), 3.94 (t, J = 9.3 Hz, 1H), 3.83 (s, 3H), 3.81 (s, 3H), 3.72 (s, 3H), 3.66 (t, J = 9.5 Hz, 1H), 3.52 (t, J = 9.2 Hz, 1H).

^{13}C NMR (151 MHz, $CDCl_3$) [ppm] δ = 159.38, 159.29, 159.23, 135.46, 135.23, 130.79, 130.70, 130.49, 129.87, 129.77, 129.75, 129.49, 129.13, 129.04, 128.96, 128.93, 113.99, 113.84, 113.75, 81.14, 79.79, 79.74, 78.81, 78.58, 78.54, 75.29, 75.14, 74.83, 74.61, 71.73, 68.73, 68.69, 68.59, 68.54, 60.40, 55.27, 55.16.

^{31}P NMR (243 MHz,) δ -1.29.

Calculated $[M+Na]^+$: 745.2385; Measured: 745.2373.

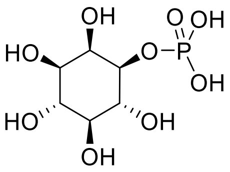

1-InsP$_1$

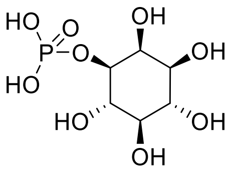

3-InsP$_1$

Protected inositol **S2a/b** (45 mg, 0.062 mmol, 1 eq.) was dissolved in *t*BuOH/H_2O (4:1, 6 mL) and 10% Pd/C (150 mg) were added under N_2 atmosphere before purging the reaction vessel with H_2. The mixture was stirred overnight and filtered through a pad of Celite®. The residue on Celite® was additionally washed with water (4 x 4 mL). The water was filtered through a 0.2 µm nylon syringe filter and washed with Et_2O once. The aqueous layer was lyophilized to yield the title product as a white solid (16 mg, quantitative). For NMR analysis cyclohexylamine (12 mg, 0.125 mmol, 2 eq.) was added.

NMR spectra are in agreement with literature[10].

Calculated $[M+H]^+$: 261.0370 Measured: 261.0370.

Ent-1 (c, 1.4, H_2O): $[\alpha]_D^{20}$ = -9,2 (free acid; Lit: -9.8), $[\alpha]_D^{20}$ = +3.3 (pH 10; Lit: +4.4)

Ent-2 (c, 1.6, H_2O): $[\alpha]_D^{20}$ = +8,1 (free acid; Lit: +9.8), $[\alpha]_D^{20}$ = -4.0 (pH 10; Lit: -4.4)

R_1 = P', R_2 = L-Cbz
or
R_1 = L-Cbz, R_2 = P'

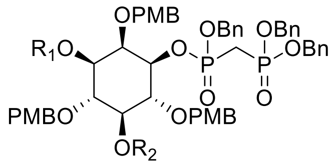

8

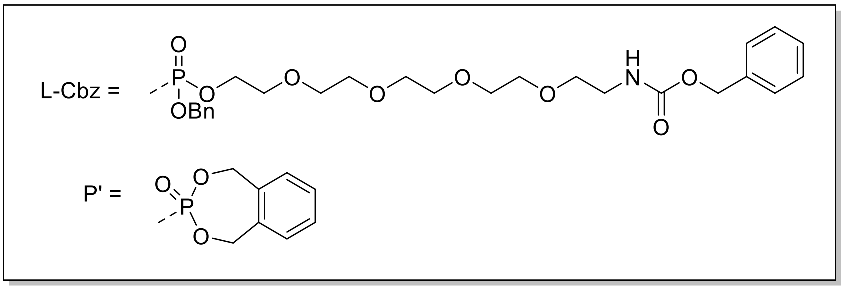

Compound **S1** (70 mg, 72.2 µmol, 1 eq.) and phosphoramidite **17** (57 mg, 93.9 µmol, 1.3 eq.) were coevaporated twice with dry ACN and dried under high vacuum for 2 h. Dry CH_2Cl_2 (2 mL) was added and the reaction mixture was cooled in an ice bath. 5-phenyl-1*H*-tetrazole (17 mg, 116 µmol, 1.6 eq.) was added as a solid. After 5-phenyl-1*H*-tetrazole was dissolved, the ice bath was removed and the mixture was additionally stirred for 16 h. The solution was cooled in a $CO_{2(s)}$/acetone bath and *m*CPBA (77%, 32 mg, 145 µmol, 2 eq.) was added carefully: the mixture was stirred for 30 min in a $CO_{2(s)}$/acetone bath and 2 h at room temperature. EtOAc (60 mL) was added and the organic phase was washed with aq. $Na_2S_2O_3$ (60 mL), sat. aq. $NaHCO_3$ (60 mL) and brine (60 mL). The organic layer was filtered through a water-repellant filter, concentrated under reduced pressure, and dried under high vacuum for 16 h. Phosphoramidite **16** (54 mg, 227 µmol, 5 eq.) was added and coevaporated twice with dry ACN (2 mL) and dried under high vacuum for 2 h. Dry ACN (2 mL) was added and the reaction mixture was cooled in an ice bath. 1*H*-Tetrazole (0.45 M in ACN, 17.6 mg, 250 µm, 0.56 mL, 5.5 eq.) was added, and the reaction mixture was allowed to reach room temperature and stirred overnight. The solution was cooled in a $CO_{2(s)}$/acetone bath and *m*CPBA (77%, 56 mg, 250 µmol, 5.5 eq.) was added carefully: the mixture was stirred for 30 min in a $CO_{2(s)}$/acetone bath and 2 h at room temperature. EtOAc (60 mL) was added and the organic phase was washed with aq. $Na_2S_2O_3$ (60 mL), sat. aq. $NaHCO_3$ (60 mL) and brine (60 mL). The organic layer was filtered through a water-repellant filter, concentrated under reduced pressure, and purified by flash chromatography (0% to 5% MeOH in CH_2Cl_2) to give the title compound (68 mg, 54.6 µmol, 56% over four steps) as a colorless oil and a mixture of multiple diastereomers and regioisomers.

^{1}H NMR (600 MHz, $CDCl_3$) [ppm] δ = 7.51 – 6.59 (m, 41H), 5.48 – 3.25 (m, 55H), 2.51 – 2.20 (m, 2H)
^{31}P NMR (243 MHz, $CDCl_3$) [ppm] δ = 20.80 – 18.60 (m, 2P), 1.61 - -2.51 (m, 2P).
^{13}C-NMR was not informative due to the formation of six diastereomers.
Calculated $[M+H]^+$: 1674.5479; Measured: 1674.5494.

R_1 = P', R_2 = L-Cbz
or
R_1 = L-Cbz, R_2 = P'

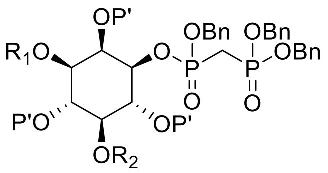

9

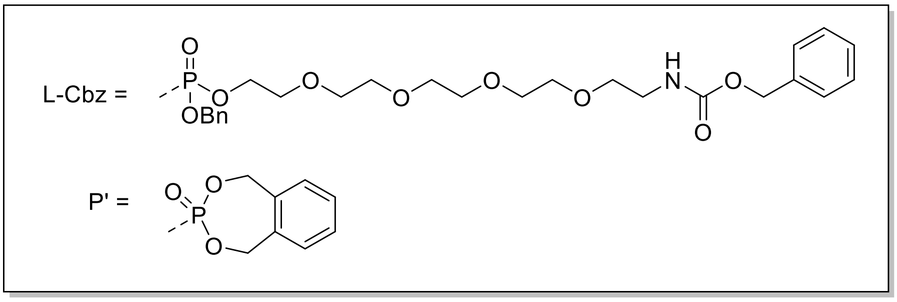

Protected inositol **8** (68 mg, 40.0 µmol, 1 eq.) was dissolved in DCM (2 mL) and cooled in an ice bath. A 20% TFA solution in CH_2Cl_2 (2 mL) was slowly added with a syringe. The deprotection progress was monitored *via* LC-MS. After completion, the reaction was diluted with EtOAc (100 mL) and washed twice with Na_2PO_4 buffer (1M, pH 7.4, 100 mL). The organic layer was filtered through a water-repellant filter, removed under reduced pressure, and used without further purification. The crude was dried under high vacuum overnight. Phosphoramidite **16** (94 mg, 395 µmol, 10 eq.) was added and the reaction mixture was coevaporated twice with dry ACN (3 mL). Dry ACN (2 mL) was added and the reaction mixture was cooled in an ice bath. 1*H*-Tetrazole (0.45 M, 30.5 mg, 435 µm, 0.97 mL, 11 eq.) was added, and the reaction mixture was allowed to reach room temperature and stirred overnight. The solution was cooled in a $CO_{2(s)}$/acetone bath and *m*CPBA (77%, 98 mg, 435 µmol, 11 eq.) was added carefully: the mixture was stirred for 30 min in a $CO_{2(s)}$/acetone bath and 2 h at room temperature. EtOAc (50 mL) was added and the organic phase was washed with aq. $Na_2S_2O_3$ (30 mL), sat. aq. $NaHCO_3$ (30 mL) and brine (30 mL). The organic layer was filtered through a water-repellant filter, concentrated under reduced pressure, and purified by flash chromatography (0% to 9% MeOH in CH_2Cl_2) followed by preparative HPLC (HPLC Method 1) to give the title compound (14 mg, 7.5 µM, 17% yield over two steps) as a white solid containing a mixture of the different diastereomers and regioisomers.

^{1}H NMR (600 MHz, $CDCl_3$) [ppm] δ = 7.64 – 6.99 (m, 41H), 5.58 – 4.89 (m, 26H), 3.66 – 3.30 (m, 16H), 2.17 – 1.85 (m, 2H).
^{31}P NMR (243 MHz, $CDCl_3$) [ppm] δ = 23.18 – 17.12 (m, 2P), -0.60 – -5.27 (m, 5P).
^{13}C-NMR was not informative due to the formation of multiple diastereomers.
Calculated $[M+2H]^{2+}$: 930.7112; Measured: 930.7110.

$R_1 = PO_3^{2-}$, R_2 = L-NH_2
or
R_1 = L-NH_2, $R_2 = PO_3^{2-}$

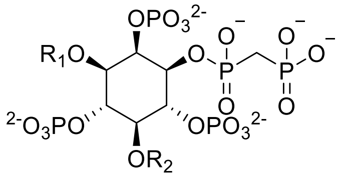

10

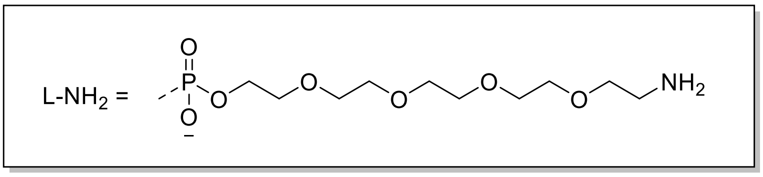

Fully protected **9** (14 mg, 7.5 µmol, 1 eq.) was dissolved in *t*BuOH/water (5 mL, 4:1), $NaHCO_3$ (5.1 mg, 60 µmol, 8 eq.) and Pd/C (10%, 40 mg, 37 µmol, 5 eq.) were added under a N_2 atmosphere before purging with H_2 gas. The mixture was stirred overnight, filtered through a Whatman® filter (0.45 µm), and lyophilized to yield the final compound as a white solid (6.1 mg, 6.3 µmol, 83%). The product was dissolved in D_2O and the pH was adjusted to 6.0. The yield was determined *via* ^{31}P-NMR spectroscopy using an internal standard (NMR method 1).

^{1}H NMR (600 MHz, D_2O pH=6.1) [ppm] δ = 5.03 (d, J = 57.3, 9.3 Hz, 1H), 4.64 – 4.45 (m, 2H), 4.45 – 4.09 (m, 5H), 3.88 – 3.74 (m, 14H), 3.30 – 3.25 (m, 2H), 2.42 (dt, J = 78.6, 18.8 Hz, 2H).
^{31}P NMR (243 MHz, D_2O pH=6.1) [ppm] δ = 20.77 – 17.35 (m, 1H), 16.79 – 13.82 (m, 1H), 2.81 – -2.48 (m, 5H).
^{13}C-NMR was not informative due to the low amount of material obtained.
Calculated $[M-2H]^{2-}$: 477.4905; Measured: 477.4909.

1a = $R_1 = PO_3^{2-}$, R_2 = L-biotin
or
1b = R_1 = L-biotin, $R_2 = PO_3^{2-}$

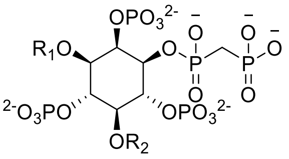

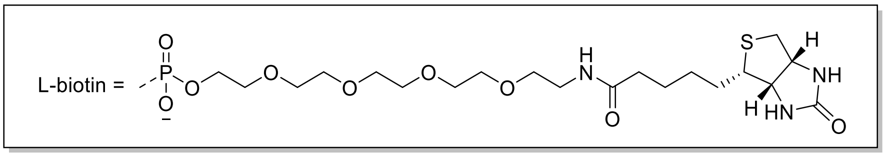

Amino-3/5***L***-1PCP-InsP$_5$ **10** (6 mg, 6.30 µmol, 1 eq.) was dissolved in 10 mL phosphate buffer (KH_2PO_4, 100 mM, pH 8.2). NHS-biotin (10.7 mg, 31.3 µmol, 5 eq.) was added and the reaction was stirred at 37 °C for 4 h. The precipitate was filtered off using a Whatman® filter (0.45 µm) and the aqueous phase was extracted with Et_2O (2 x 30 mL) and concentrated by lyophilization. The crude was dissolved in 5 mL and pH was adjusted to 3.0. The crude was purified by preparative HPLC (HPLC method 2). Fractions containing the product were identified using an MDD assay[13], combined, and the solvent removed by lyophilization which affords the product as a white solid (6.0 mg, 5.0 µM, 81%). The product was dissolved in D_2O and the pH was adjusted to 6.0. The yield was determined *via* ^{1}H-NMR spectroscopy using an internal standard (NMR method 2).

^{1}H-NMR (600 MHz, D_2O, pH=6.1): [ppm] δ = 5.10 (d, *J* = 9.2 Hz, 1H), 4.66 (dq, *J* = 15.5, 9.3, 7.2 Hz, 4H), 4.58 – 4.37 (m, 3H), 4.32 – 4.10 (m, 2H), 3.94 – 3.58 (m, 16H), 3.43 (dt, *J* = 29.1, 4.9 Hz, 3H), 3.06 (dd, *J* = 13.1, 5.0 Hz, 1H), 2.90 – 2.79 (m, 1H), 2.57 (s, 2H), 2.34 (t, *J* = 7.3 Hz, 2H), 1.88 – 1.57 (m, 4H), 1.48 (p, *J* = 7.4 Hz, 2H).

^{31}P-NMR (243 MHz, D_2O, pH=6.1): [ppm] δ = 17.08 (3***L***, major, 80%), 14.94 (5***L***, minor, 20%), 13.19 (5***L***, minor, 20%), 11.96 (3***L***, major, 80%), -1.74, -2.16, -2.27, -2.53, -2.63, -2.73, -2.92, -3.90, -3.95.

^{13}C-NMR was not informative due to the low amount of material obtained.

HRMS Calculated [M+2Na]$^{2+}$: 590.5293; Measured: 590.5262.

^{1}H-^{31}P-HMBC, ^{1}H-^{13}C-BIRD-HMQC-CLIP-COSY, and ^{1}H-^{13}C-DEPT-HMBC spectra were recorded for complete characterization and analysis of the major product 3***L***-1PCP-InsP$_5$ (**Supplementary Figure S2**)

	**^{1}H in ppm**	**^{13}C in ppm**	**^{31}P in ppm**
myo-Ins 1 position	4.42	74.56	17.03 alpha
myo-Ins 2 position	4.98	78.77	-1.74
myo-Ins 3 (with linker)	4.26	76.05	-3.95
myo-Ins 4 position	4.57	77.90	-2.63
myo-Ins 5 position	4.30	80.37	-3.90
myo-Ins 6 position	4.57	78.72	-2.53
PEG (CH_2) at 3 position	4.23	67.88	-2.92
PCH_2P 1 position	2.35	31.28	17.03 alpha

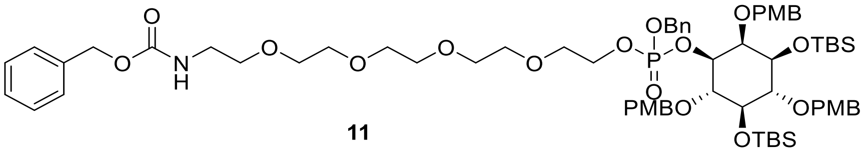

11

Inositol **5a** (150 mg, 195 µmol, 1 eq.) and phosphoramidite **17** (237 mg, 390 µmol, 2 eq.) were coevaporated twice with dry ACN and dried under high vacuum for 2 h. Dry ACN (1 mL) was added and the reaction mixture was cooled in an ice bath. 1*H*-Tetrazole (0.45 M, 31.4 mg, 448 µm, 0.96 mL, 2.3 eq.) was added, and the reaction mixture was allowed to reach room temperature and stirred overnight. The solution was cooled in a $CO_{2(s)}$/acetone bath and *m*CPBA (77%, 131 mg, 585 µmol, 3 eq.) was added carefully: the mixture was stirred for 30 min in a $CO_{2(s)}$/acetone bath and 2 h at room temperature. EtOAc (120 mL) was added and the organic phase was washed with aq. $Na_2S_2O_3$ (100 mL), sat. aq. $NaHCO_3$ (100 mL) and brine (100 mL). The organic layer was filtered through a water-repellant filter, concentrated under reduced pressure, and purified by flash chromatography (0% to 4% MeOH in CH_2Cl_2) to give the title compound (147 mg, 113 µmol, 58%) as a colorless oil and a mixture of two diastereomers.

^{1}H NMR (600 MHz, $CDCN_3$) [ppm] δ = 7.42 – 7.24 (m, 16H), 7.00 – 6.77 (m, 6H), 5.11 – 4.98 (m, 4H), 4.95 – 4.81 (m, 2H), 4.79 – 4.59 (m, 4H), 4.41 (ddd, *J* = 9.8, 7.2, 2.6 Hz, 1H), 4.35 (ddd, *J* = 9.7, 7.2, 2.6 Hz, 1H), 4.18 (dt, J = 19.3, 2.5 Hz, 1H), 4.15 – 3.98 (m, 2H), 3.87 – 3.73 (m, 9H), 3.71 – 3.46 (m, 18H), 3.27 (td, J = 5.6, 1.9 Hz, 2H), 0.88-0.84 (m, 18H), 0.17- -0.10 (m, 12H).
^{31}P NMR (243 MHz, CD_3CN) [ppm] δ = -1.63, -1.69.
^{13}C NMR (151 MHz, CD_3CN) [ppm] δ = 159.27, 158.95, 158.48, 156.38, 137.44, 136.29, 131.32, 131.27, 131.22, 130.80, 130.76, 129.26, 129.15, 129.10, 129.07, 128.59, 128.55, 128.43, 128.08, 127.91, 127.84, 127.73, 127.70, 113.66, 113.63, 113.32, 113.28, 113.12, 81.64, 80.42, 80.35, 80.30, 78.51, 78.47, 75.14, 75.08, 74.62, 74.36, 73.41, 70.16, 70.14, 70.09, 70.06, 69.92, 69.63, 69.59, 69.55, 69.50, 69.43, 69.23, 69.19, 68.96, 68.93, 67.11, 67.06, 66.91, 66.87, 65.82, 54.92, 54.85, 54.80, 54.32, 40.59, 29.18, 25.54, 25.40, 25.38, 17.60, -4.44, -4.85, -4.88, -5.50.
Calculated $[M+Na]^+$: 1314.5953 Measured: 1314.5961.

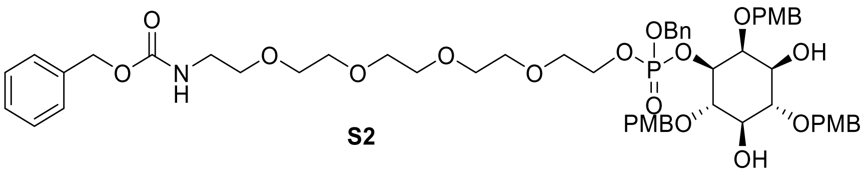

S2

Compound **11** (140 mg, 108 µmol, 1 eq.) was dissolved in THF (5 mL) and cooled under an ice bath. TBAF in THF (1 M, 113 mg, 433 µmol, 0.5 mL, 4 eq.) was added dropwise. The reaction mixture was allowed to reach room temperature and was stirred for 2 h. The reaction mixture was diluted with EtOAc (100 mL) and the organic layer was washed twice with saturated $CaCl_2$ solution (100 mL) and brine (100 mL). The organic layer was filtered through a water-repellant filter, concentrated under reduced pressure, and purified by flash chromatography (0% to 4% MeOH in CH_2Cl_2) to give the title compound (110 mg, 101 µmol, 97%) as a colorless oil and a mixture of two diastereomers.

^{1}H NMR (600 MHz, $CDCl_3$) [ppm] δ = 7.36-7.25 (m, 16H), 6.91-6.83 (m, 6H), 5.47 (m, 1H), 5.12-5.07(m, 4H), 4.83, 4.82-4.65(m, 6Hj), 4.29 (ddd, J = 10.3, 8.0, 2.6 Hz, 1H), 4.22 (m, 1H), 4.17-4.12 (m, 2H), 3.88-3.84 (m, 1H), 3.83-3.79 (m, 9H), 3.65-3.47 (m,18H), 3.39 (q, J = 5.4 Hz, 2H).

^{31}P NMR (243 MHz, $CDCl_3$) [ppm] δ = -1.49, -1.75.

^{13}C NMR (151 MHz, $CDCl_3$) [ppm] δ = 159.35, 159.25, 156.50, 136.66, 135.85, 130.75, 130.62, 129.73, 129.63, 129.33, 128.58, 128.48, 128.12, 128.04, 127.93, 113.94, 113.84, 113.79, 113.77, 81.00, 79.69, 78.72, 75.11, 74.83, 74.60, 71.76, 70.51, 70.46, 70.23, 70.04, 69.91, 69.34, 66.98, 66.60, 60.38, 55.27, 53.41, 40.89.

Calculated $[M+Na]^+$: 1086.4223 Measured: 1086.4214.

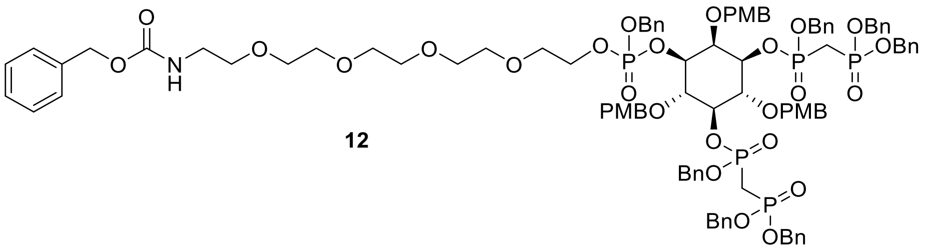

12

Diol **S2** (100 mg, 95.4 µmol, 1 eq.) and phosphoramidite **15** (293 mg, 572 µmol, 6.0 eq.) were coevaporated twice with dry ACN and dried under high vacuum for 2 h. Dry ACN (1 mL) was added and the reaction mixture was cooled in an ice bath. Tetrazole (0.45 M, 60.2 mg, 858 µm, 1.90 mL, 9 eq.) was added, and the reaction mixture was allowed to reach room temperature and stirred overnight. The solution was cooled in a $CO_{2(s)}$/acetone bath and *m*CPBA (77%, 171 mg, 763 µmol,8 eq.) was added carefully: the mixture was stirred for 30 min in a $CO_{2(s)}$/acetone bath and 2 h at room temperature. EtOAc (120 mL) was added and the organic phase was washed with aq. $Na_2S_2O_3$ (100 mL), sat. aq. $NaHCO_3$ (100 mL) and brine (100 mL). The organic layer was filtered through a water-repellant filter, concentrated under reduced pressure, and purified by flash chromatography (0% to 5% MeOH in CH_2Cl_2) to give the title compound (104 mg, 54.6 µmol, 57%) as a colorless oil and a mixture of two diastereomers.

^{1}H NMR (600 MHz, CD_3CN) [ppm] δ = 7.54 – 7.11 (m, 46H), 6.93 – 6.63 (m, 6H), 5.19 – 4.84 (m, 17H), 4.82 – 4.39 (m, 9H), 4.18 – 4.00 (m, 2H), 3.99 – 3.90 (m, 2H), 3.77 (dd, J = 5.9, 2.5 Hz, 2H), 3.72-3.56 (,7H), 3.56-3.45 (m, 15H), 3.25 (q, J = 5.6 Hz, 1H), 2.74 – 2.38 (m, 2H).
^{31}P NMR (243 MHz, CD_3CN) [ppm] δ = 21.50 – 18.28 (m, 4H), -1.54 – -2.43 (m, 1H).
^{13}C NMR (151 MHz, CD_3CN) [ppm] δ = 159.30, 159.06, 156.41, 137.44, 136.44, 136.39, 130.84, 130.06, 129.61, 129.49, 129.42, 129.33, 129.26, 129.20, 128.62, 128.58, 128.52, 128.49, 128.43, 128.33, 128.28, 128.25, 128.17, 128.08, 128.02, 127.98, 127.94, 127.87, 127.84, 127.73, 127.69, 113.68, 113.65, 113.59, 113.54, 113.51, 113.48, 113.44, 113.41, 78.26, 77.93, 77.65, 77.25, 77.14, 76.92, 75.30, 73.33, 70.13, 70.12, 70.05, 69.90, 69.45, 69.19, 68.70, 67.69, 67.55, 67.31, 65.83, 54.91, 54.84, 54.80, 54.77, 40.59, 25.63.
Calculated $[M+Na]^+$: 1942.6108 Measured: 1942.6110

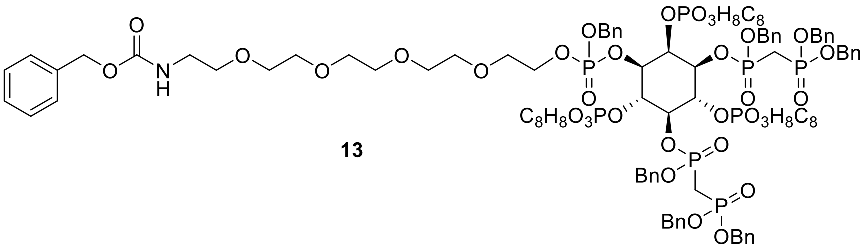

13

Protected inositol **12** (50 mg, 26.0 µmol, 1 eq.) was dissolved in CH_2Cl_2 (1 mL) and cooled in an ice bath. A 15% TFA solution in CH_2Cl_2 (1 mL) was added with a syringe. The deprotection progress was monitored *via* LC-MS. After completion, the reaction was diluted with EtOAc (100 mL) and washed twice with Na_2PO_4 buffer (1 M, pH 7.4, 100 mL). The organic layer was filtered through a water-repellant filter, removed under reduced pressure, and used without further purification. The crude was dried under a high vacuum overnight. Phosphoramidite **16** (115 mg, 480 µmol, 15 eq.) was added and the reaction mixture was coevaporated twice with dry ACN (3 mL). Dry ACN (2 mL) was added and the reaction mixture was cooled in an ice bath. Tetrazole (0.45 M, 38.0 mg, 544 µm, 1.21 mL, 17 eq.) was added, and the reaction mixture was allowed to reach room temperature and stirred overnight. The solution was cooled in a $CO_{2(s)}$/acetone bath and *m*CPBA (77%, 136 mg, 608 µmol, 19 eq.) was added carefully: the mixture was stirred for 30 min in a $CO_{2(s)}$/acetone bath and 2 h at room temperature. EtOAc (80 mL) was added and the organic phase was washed with aq. $Na_2S_2O_3$ (15 mL), sat. aq. $NaHCO_3$ (15 mL) and brine (10 mL). The organic layer was filtered through a water-repellant filter, concentrated under reduced pressure and purified by flash chromatography (0% to 9% MeOH in CH_2Cl_2) followed by preparative HPLC (YMC Actus Triart C18 (15 x 200 mm) column, solvent: MilliQ+ 0.1 % TFA (A), acetonitrile +0.1 % TFA (B), 35 mL/min. Gradient: 70% B for 1 minute, followed by a gradient to 95% B for 7 minutes and a wash at 100% B for 2 minutes) to give the title compound (38 mg, 71% yield over two steps) as a white solid containing a mixture of the different diastereomers.

^{1}H NMR (600 MHz, $CDCl_3$) [ppm] δ = 7.67 – 7.08 (m, 46H), 6.18 – 4.73 (m, 34H), 4.52 – 4.20 (m, 2H), 3.97 – 3.19 (m, 18H), 3.07 (m, 4H).

^{31}P NMR (243 MHz, $CDCN_3$) [ppm] δ = 19.26 – 15.99 (m, 4P), -0.92 – -7.57 (m, 4P).

^{13}C-NMR was not informative due to the formation of multiple diastereomers.

Calculated $[M+Na]^+$: 2128.4781 Measured: 2128.4792

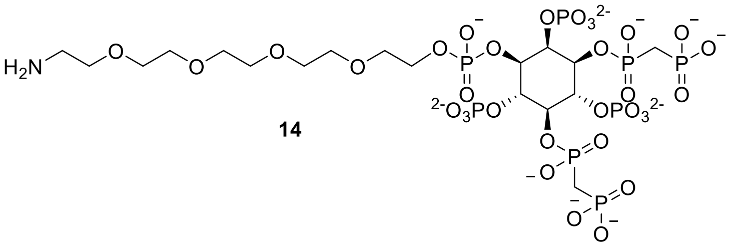

14

Fully protected **13** (38 mg, 18 µmol, 1 eq.) was dissolved in *t*BuOH/water (5 mL, 4:1), $NaHCO_3$ (12.2 mg, 144 µmol, 8 eq.) and Pd/C (10%, 191 mg, 180 µmol, 10 eq.) were added under a N_2 atmosphere before purging with H_2 gas. The mixture was stirred overnight, filtered through a Whatman® filter (0.45 µm), and lyophilized to yield the final compound as a white solid (18.2 mg, 98%). The product was dissolved in D_2O and the pH was adjusted to 6.0. The yield was determined *via* ^{31}P-NMR spectroscopy using an internal standard (NMR method 1).

^{1}H NMR (600 MHz, D_2O pH=6.1) [ppm] δ = 4.70, 4.69, 4.31, 4.29, 4.22, 4.22, 4.19, 4.03, 3.95, 3.94, 3.92, 3.91, 3.49, 3.48, 2.99, 2.98, 2.98, 2.27, 2.24, 2.24, 2.21, 2.20, 2.17, 2.16, 2.14, 2.13, 2.11, 2.10.
^{31}P NMR (243 MHz, D_2O pH=6.1) [ppm] δ = 17.70 (d, J = 11.4 Hz, 2P), 11.74 (d, J = 11.1 Hz, 1P), 11.50 (d, J = 11.5 Hz, 1P) -2.85(2P), -3.03(1P), -4.13(1P).
^{13}C NMR (151 MHz, D_2O pH=6.1) [ppm] δ = 75.75, 75.37, 75.21, 74.25, 72.88, 71.53, 69.45, 69.40, 69.34, 69.31, 69.28, 69.11, 66.25, 64.94, 64.91, 38.75, 28.61, 27.80, 27.70, 26.98, 26.91.
^{31}P NMR (243 MHz, D_2O pH=6.1) [ppm] δ = 17.70 (d, J = 11.4 Hz, 2P), 11.74 (d, J = 11.1 Hz, 1P), 11.50 (d, J = 11.5 Hz, 1P) -2.85(2P), -3.03(1P), -4.13(1P).
Calculated $[M-2H]^{2-}$: 516.4840 Measured: 516.4847

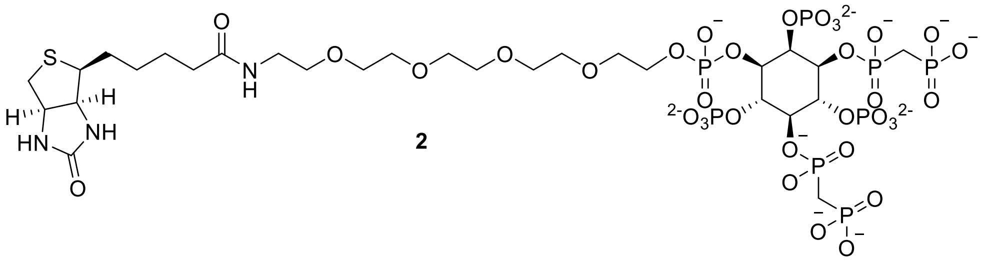

2

Amino-3***L***-1,5(PCP)$_2$-InsP$_4$ **14** (6 mg, 5.80 µmol, 1 eq.) was dissolved in 10 mL phosphate buffer (KH_2PO_4, 100 mM, pH 8.2). NHS-biotin (19.4 mg, 57.3 µmol, 10 eq.) was added and the reaction was stirred at 37 °C for 4 h. The precipitate was filtered off using a Whatman® filter (0.45 µm) and the aqueous phase was extracted with Et_2O (2 x 30 mL) and concentrated by lyophilization. The crude was dissolved in 5 mL and pH was adjusted to 3.0. The crude was purified by preparative HPLC (HPLC method 2). Fractions containing the product were identified using an MDD assay[20], combined, and the solvent was removed by lyophilization which affords the product as a white solid (4.1 mg, 58%). The product was dissolved in D_2O and the pH was adjusted to 6.0. The yield was determined *via* ^{1}H-NMR spectroscopy using an internal standard (NMR method 2).

^{1}H-NMR (600 MHz, D_2O, pH=6.1): [ppm] δ = 4.95 (d, J = 9.2 Hz, 1H), 4.65 (dd, J = 8.0, 4.8 Hz, 1H), 4.59 – 4.42 (m, 3H), 4.36 (q, J = 9.7 Hz, 2H), 4.21 (dd, J = 12.0, 6.0 Hz, 3H), 3.82 – 3.73 (m, 14H), 3.67 (t, J = 5.3 Hz, 2H), 3.43 (t, J = 5.3 Hz, 2H), 3.38 (dd, J = 9.3, 4.9 Hz, 1H), 3.04 (dd, J = 13.1, 5.0 Hz, 1H), 2.82 (d, J = 13.0 Hz, 1H), 2.45 (t, 2H), 2.41 – 2.27 (m, 4H), 1.70 (m, J = 43.9, 30.0, 14.8, 7.4 Hz, 4H), 1.45 (m, J = 7.5 Hz, 2H).

^{31}P-NMR (243 MHz, D_2O, pH=6.1): [ppm] δ = 17.12, 16.97, 10.95, 10.94, -3.52, -3.56, -3.63, -4.68.

^{13}C-NMR was not informative due to the low amount of material obtained.

HRMS Calculated $[M-2H]^{2-}$: 629.5228; Measured: 629.5202.

^{1}H-^{31}P-HMBC, ^{1}H-^{13}C-BIRD-HMQC-CLIP-COSY, and ^{1}H-^{13}C-DEPT-HMBC spectra were recorded for complete characterization and analysis (**Supplementary Figure S3**).

	**^{1}H in ppm**	**^{13}C in ppm**	**^{31}P in ppm**
myo-Ins 1 position	4.43	74.56	16.97 alpha
myo-Ins 2 position	4.91	78.70	-4.68
myo-Ins 3 (with linker)	3.27	76.17	-3.63
myo-Ins 4 position	4.56	77.32	-3.56
myo-Ins 5 position	4.40	78.23	17.12 alpha
myo-Ins 6 position	4.56	78.06	-3.52
PEG (CH_2) at 3 position	4.22	67.93	-3.63
PCH_2P 5 position	2.49	31.34	17.12 alpha
PCH_2P 1 position	2.39	31.29	16.97 alpha

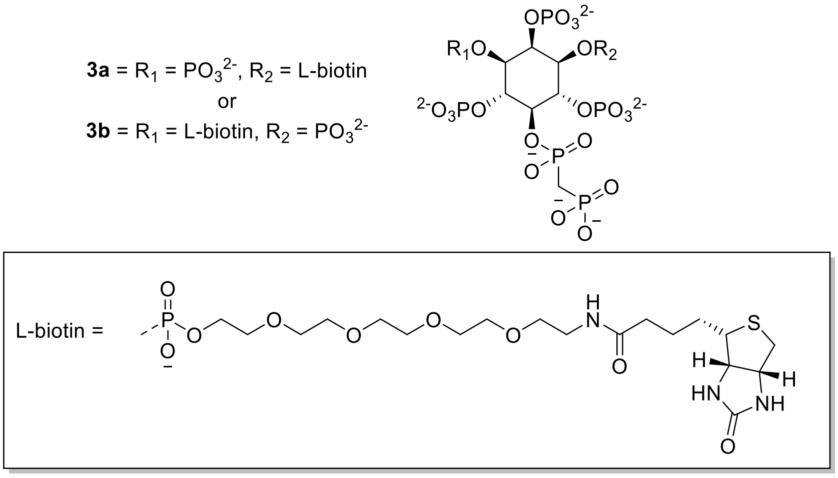

Amino-1/3***L***-5PCP-InsP_5 **18** (9.5 mg, 9.9 µmol, 1 eq.) was prepared according to a published procedure[3] and was dissolved in 10 mL phosphate buffer (KH_2PO_4, 100 mM, pH 8.2). NHS-biotin (16.9 mg, 49.6 µmol, 5 eq.) was added and the reaction was stirred at 37 °C for 4 h. The precipitate was filtered using a Whatman® filter (0.45 µm) and the aqueous phase was extracted with Et_2O (2 x 30 mL) and concentrated by lyophilization. The crude was dissolved in 5 mL and pH was adjusted to 3.0. The crude was purified by preparative HPLC (HPLC method 2). Fractions containing the product were identified using an MDD assay[20], combined, and the solvent was removed by lyophilization which afforded the product as a white solid (6.0 mg, 51 %). The product was dissolved in D_2O and the pH was adjusted to 6.1. The yield was determined *via* ^{31}P-NMR spectroscopy and ^{1}H-NMR spectroscopy using an internal standard (NMR methods 1 and 2).

^{1}H-NMR (600 MHz, D_2O, pH=6.1): [ppm] δ = 4.95 (dt, *J* = 9.6, 2.5 Hz, 1H), 4.63 – 4.52 (m, 3H), 4.46 (q, *J* = 9.4 Hz, 1H), 4.38 (td, *J* = 7.9, 3.5 Hz, 3H), 4.21 – 4.03 (m, 2H), 3.75 – 3.60 (m, 14H), 3.57 (t, *J* = 5.3 Hz, 2H), 3.33 (t, *J* = 5.3 Hz, 2H), 3.31 – 3.25 (m, 1H), 2.94 (dd, *J* = 13.1, 5.0 Hz, 1H), 2.73 (d, *J* = 13.1 Hz, 1H), 2.58 (t, *J* = 20.3 Hz, 2H), 2.22 (t, *J* = 7.3 Hz, 2H), 1.72 – 1.47 (m, 4H), 1.41 – 1.30 (m, 2H).

^{31}P-NMR (243 MHz, D_2O, pH=6.1): [ppm] δ = 19.41, 18.74, 033, −0.01, −0.21, −1.48.

^{13}C-NMR (151 MHz, D_2O, pH=6.1): [ppm] δ = 177.00, 165.35, 76.04, 73.12, 70.02, 69.65.

HRMS Calculated $[M-2H]^{2-}$: 590.5293; Measured: 590.5264.

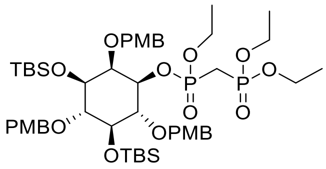

20

Enantiomer **5b** (50 mg, 65.0 µmol, 1 eq.) and phosphoramidite **26** (42.6 mg, 130 µmol, 2.0 eq.) were coevaporated twice with dry ACN and dried under high vacuum for 2 h. Dry ACN (2 mL) was added and the reaction mixture was cooled in an ice bath. 1*H*-Tetrazole (0.45 M, 18.2 mg, 260 µm, 0.57 mL, 4 eq.) was added, and the reaction mixture was allowed to reach room temperature and stirred overnight. The solution was cooled in a $CO_{2(s)}$/acetone bath and *m*CPBA (77%, 33.5 mg, 149.5 µmol, 2.3 eq.) was added carefully: the mixture was stirred for 30 min in a $CO_{2(s)}$/acetone bath, and 2 h at room temperature. EtOAc (120 mL) was added and the organic phase was washed with aq. $Na_2S_2O_3$ (100 mL), sat. aq. $NaHCO_3$ (100 mL) and brine (100 mL). The organic layer was filtered through a water-repellant filter, concentrated under reduced pressure, and purified by flash chromatography (0% to 3% MeOH in CH_2Cl_2) to give the title compound (40 mg, 39.5 µmol, 61%) as a colorless oil and a mixture of two diastereomers.

^{1}H NMR (600 MHz, $CDCl_3$) [ppm] δ = 7.42 – 7.32 (m, 3H), 7.32 – 7.26 (m, 2H), 7.23 (d, *J* = 8.3 Hz, 1H), 6.95 – 6.91 (m, 2H), 6.89 – 6.82 (m, 4H), 4.98 – 4.88 (m, 2H), 4.83 – 4.62 (m, 4H), 4.34 – 3.99 (m, 8H), 3.89 – 3.79 (m, 10H), 3.76 – 3.62 (m, 2H), 3.54 (s, 1H), 2.45 – 2.04 (m, 2H), 1.36 – 1.26 (m, 9H), 0.86 (d, *J* = 2.7 Hz, 9H), 0.82 (d, *J* = 1.9 Hz, 9H), 0.15 (d, *J* = 5.7 Hz, 3H), 0.04 (s, 3H), 0.02 (d, *J* = 4.6 Hz, 3H), -0.09 (s, 3H).

^{31}P NMR (243 MHz, $CDCl_3$) [ppm] δ = 20.75, 19.31, 19.27, 18.93.

^{13}C NMR (151 MHz, $CDCl_3$) [ppm] δ = 159.00, 158.62, 158.10, 131.32, 131.00, 130.95, 128.95, 128.80, 128.56, 128.43, 128.24, 127.84, 127.38, 113.58, 113.55, 113.46, 113.29, 113.04, 81.48, 81.16, 80.77, 80.39, 80.24, 75.13, 74.72, 74.53, 73.86, 63.43, 62.88, 62.65, 62.52, 62.34, 62.24, 55.20, 55.07, 46.58, 25.92 (3C), 25.84 (3C), 17.89, 16.23, 16.19, -3.95, -3.99, -4.29, -4.90.

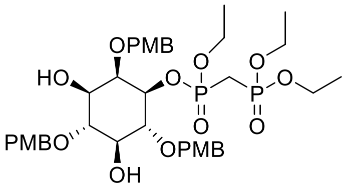

21

Compound **20** (327 mg, 323 µmol, 1 eq.) was dissolved in dry THF (18 mL) and cooled under an ice bath. TBAF in THF (1 M, 338 mg, 1.29 mmol, 1.29 mL, 4 eq.) was added dropwise. The reaction mixture was allowed to reach room temperature and was stirred for 2 h. The deprotection progress was monitored *via* LC-MS. The reaction mixture was diluted with EtOAc (150 mL) and the organic layer was washed twice with saturated $CaCl_2$ solution (150 mL) and brine (150 mL). The organic layer was filtered through a water-repellant filter, concentrated under reduced pressure, and purified by flash chromatography (0% to 4% MeOH in CH_2Cl_2) to give the title compound (198 mg, 252 µmol, 78%) as a colorless oil and a mixture of two diastereomers.

^{1}H NMR (600 MHz, $CDCl_3$) [ppm] δ = 7.29 (dddd, *J* = 15.0, 8.9, 5.8, 3.4 Hz, 6H), 6.98 – 6.80 (m, 6H), 4.88 – 4.53 (m, 6H), 4.41 (dtd, *J* = 22.5, 9.6, 2.7 Hz, 1H) 4.30 – 4.00 (m, 7H), 3.86 – 3.78 (m, 10H), 3.72 – 3.41 (m, 3H), 2.49 – 2.28 (m, 2H), 1.34 – 1.25 (m, 9H).
^{31}P NMR (243 MHz $CDCl_3$) [ppm] δ = 20.76, 19.56, 19.15, 19.07.
^{13}C NMR (151 MHz, $CDCl_3$) [ppm] δ = 159.41, 159.36, 159.31, 130.63, 130.53, 130.50, 130.04, 129.77, 129.71, 129.67, 129.50, 129.47, 129.33, 114.14, 114.01, 113.96, 113.92, 113.84, 83.85, 81.17, 80.78, 79.89, 79.85, 79.74, 74.59, 74.37, 74.26, 71.64, 63.46, 63.22, 63.10, 62.76, 62.45, 55.27, 46.29, 16.33, 16.29, 16.20.

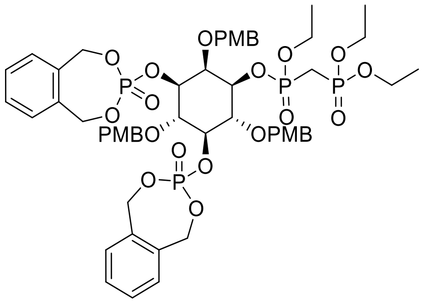

22

Protected inositol **21** (200 mg, 255 µmol, 1 eq.) and phosphoramidite **16** (244 mg, 1.02 mmol, 4 eq.) were coevaporated twice with dry ACN (3 mL). Dry ACN (8 mL) was added and the reaction mixture was cooled in an ice bath. 1*H*-Tetrazole (0.45 M, 78.8 mg, 1.12 mm, 2.50 mL, 4.4 eq.) was added, and the reaction mixture was allowed to reach room temperature and stirred overnight. The solution was cooled in a $CO_{2(s)}$/acetone bath and *m*CPBA (77%, 257 mg, 1.15 mmol, 4.5 eq.) in CH_2Cl_2 (10 mL) was added carefully: the mixture was stirred for 30 min in a $CO_{2(s)}$/acetone bath and 2 h at room temperature. EtOAc (150 mL) was added and the organic phase was washed with aq. $Na_2S_2O_3$ (130 mL), sat. aq. $NaHCO_3$ (130 mL) and brine (130 mL). The organic layer was filtered through a water-repellant filter, concentrated under reduced pressure, and purified by flash chromatography (0% to 10% MeOH in CH_2Cl_2) followed by preparative HPLC (HPLC Method 1) to give the title compound (211 mg, 183 µM, 72% yield over two steps) as a white solid containing a mixture of six diastereomers.

^{1}H NMR (600 MHz, $CDCl_3$) [ppm] δ = 7.43 – 7.26 (m, 10H), 7.13 (ddq, *J* = 8.7, 5.5, 3.4, 2.9 Hz, 4H), 6.93 – 6.81 (m, 4H), 6.71 – 6.65 (m, 2H), 5.34 – 4.47 (m, 16H), 4.47 – 4.28 (m, 2H), 4.28 – 3.91 (m, 8H), 3.83 – 3.77 (m, 6H), 3.70 (d, *J* = 4.7 Hz, 3H), 2.40 – 2.10 (m, 2H), 1.31 – 1.14 (m, 9H).

^{31}P NMR (243 MHz, $CDCl_3$) [ppm] δ = 20.57, 19.14, 18.88, 18.86, 1.38, 1.29, -1.62, -1.85.

^{13}C-NMR was not informative due to the formation of six diastereomers.

Calculated $[M+H]^+$: 1147.3171; Measured: 1147.3176.

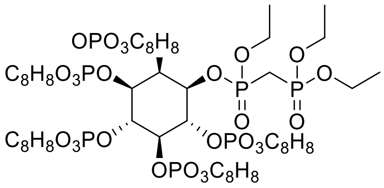

24

Protected inositol **22** (100 mg, 127 µmol, 1 eq.) was dissolved in CH_2Cl_2 (7 mL) and cooled in an ice bath. A 20% TFA solution in CH_2Cl_2 (7 mL) was added with a syringe. The deprotection progress was monitored *via* LC-MS. After completion, the reaction was diluted with EtOAc (100 mL) and washed twice with Na_2PO_4 buffer (1M, pH 7.4, 100 mL). The organic layer was filtered through a water-repellant filter, removed under reduced pressure, and used without further purification. The crude was dried under high vacuum overnight. Phosphoramidite **16** (182 mg, 762 µmol, 6 eq.) was added and the reaction mixture was coevaporated twice with dry ACN (3 mL). Dry ACN (2 mL) was added and the reaction mixture was cooled in an ice bath. 1*H*-Tetrazole (0.45 M, 57.9 mg, 862 µm, 1.84 mL, 6.5 eq.) was added, and the reaction mixture was allowed to reach room temperature and stirred overnight. The solution was cooled in a $CO_{2(s)}$/acetone bath and *m*CPBA (77%, 199 mg, 889 µmol, 7 eq.) was added carefully: the mixture was stirred for 30 min in a $CO_{2(s)}$/acetone bath and 2 h at room temperature. EtOAc (200 mL) was added and the organic phase was washed with aq. $Na_2S_2O_3$ (200 mL), sat. aq. $NaHCO_3$ (200 mL) and brine (200 mL). The organic layer was filtered through a water-repellant filter, concentrated under reduced pressure, and purified by flash chromatography (0% to 6% MeOH in CH_2Cl_2) followed by preparative HPLC (HPLC Method 1) to give the title compound (80 mg, 60.0 µM, 68% yield over two steps) as a white solid containing a mixture of the different diastereomers.

^{1}H NMR (600 MHz, $CDCl_3$) [ppm] δ = 7.45 – 7.26 (m, 20H), 5.77 – 4.97 (m, 26H), 4.45 (p, *J* = 7.2 Hz, 2H), 4.25 (ddd, *J* = 15.5, 13.4, 7.3 Hz, 4H), 2.79 (ddd, *J* = 22.8, 20.5, 15.5 Hz, 2H), 1.48 (t, *J* = 7.0 Hz, 3H), 1.41 (t, *J* = 7.1 Hz, 6H).

^{31}P NMR (243 MHz, $CDCl_3$) [ppm] δ = 22.60, 19.29, -2.45, -3.61, -3.69, -4.40, -4.57.

^{13}C-NMR was not informative due to the formation of multiple diastereomers.

Calculated [M+Na]$^+$: 1333.1844; Measured: 1333.1838.

NMR spectra

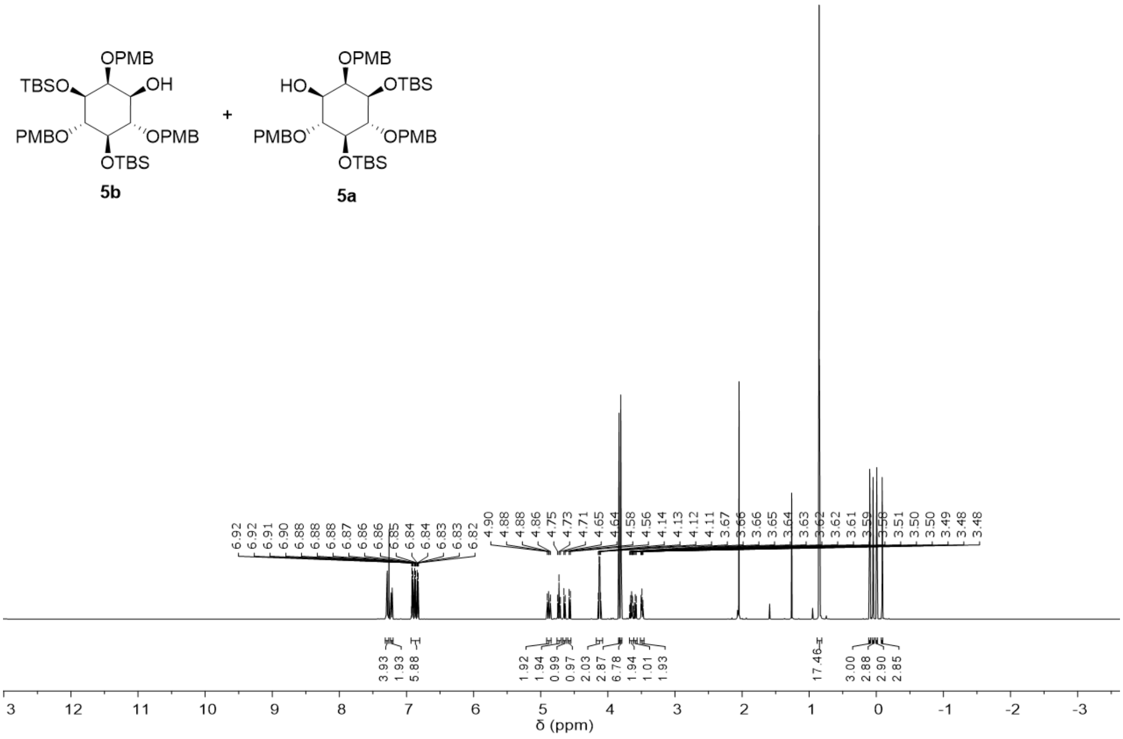

^{1}H spectrum of compound **5**.

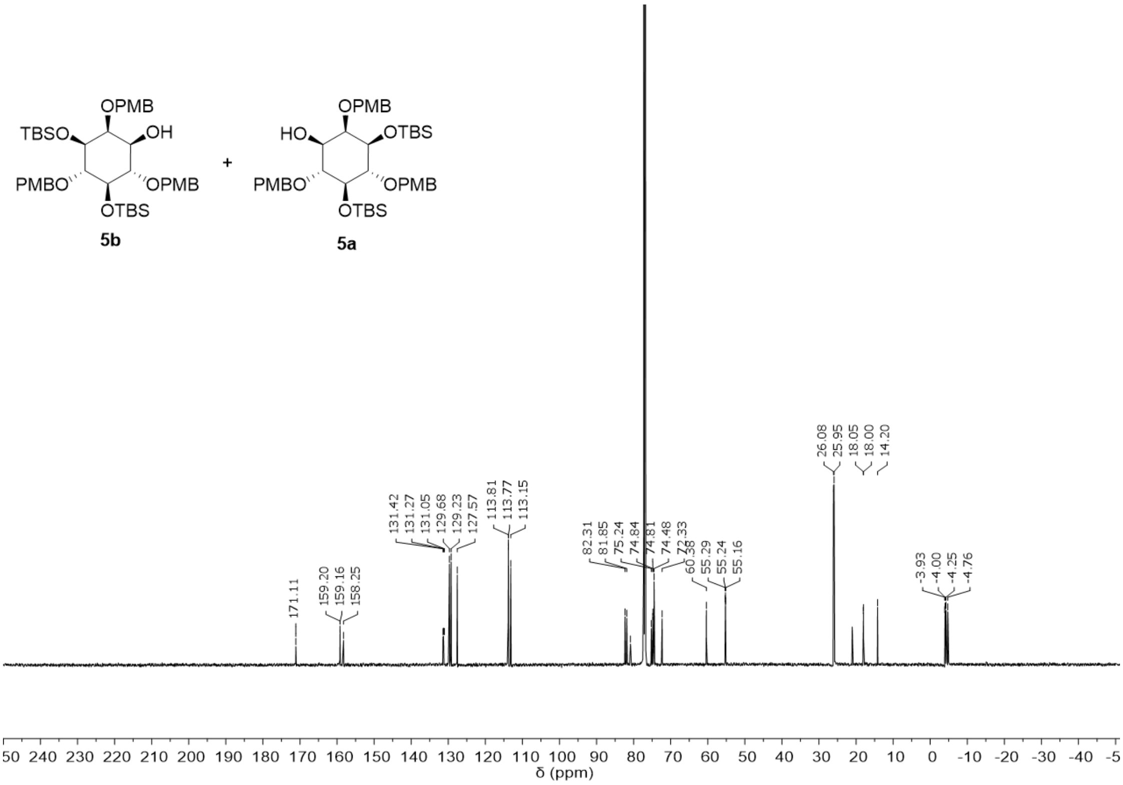

^{13}C spectrum of compound **5**.

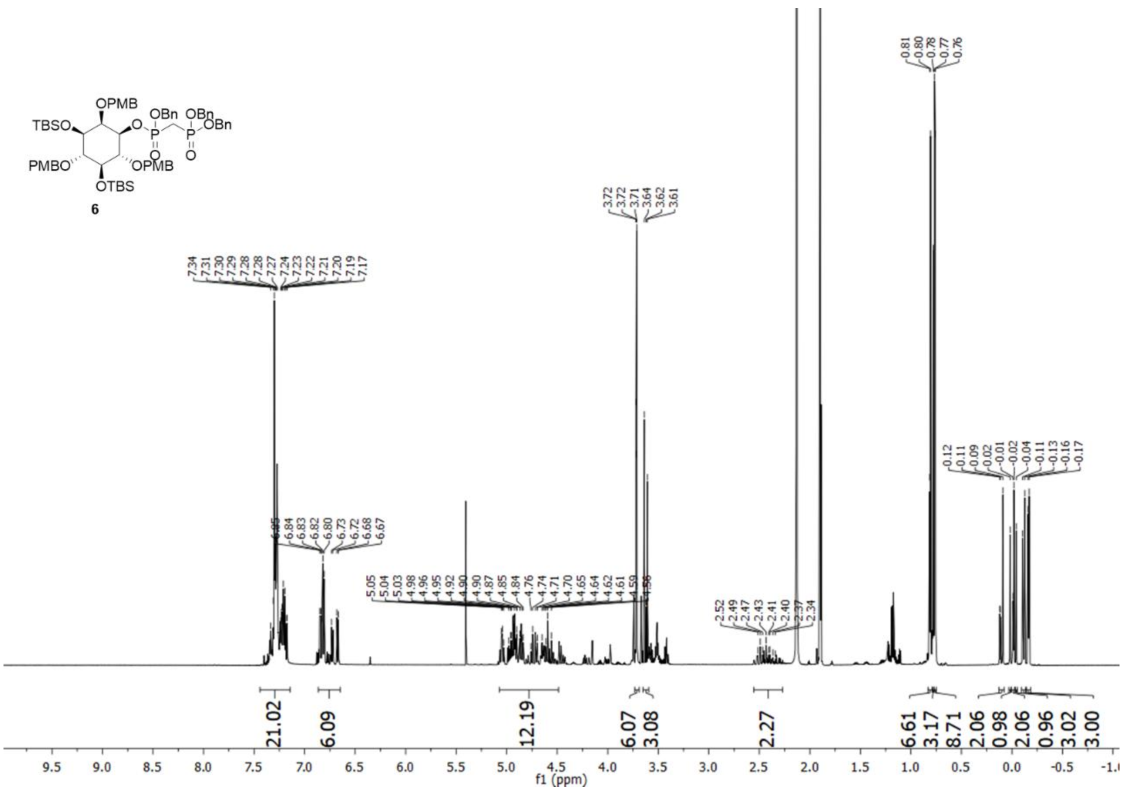

^{1}H spectrum of compound **6**.

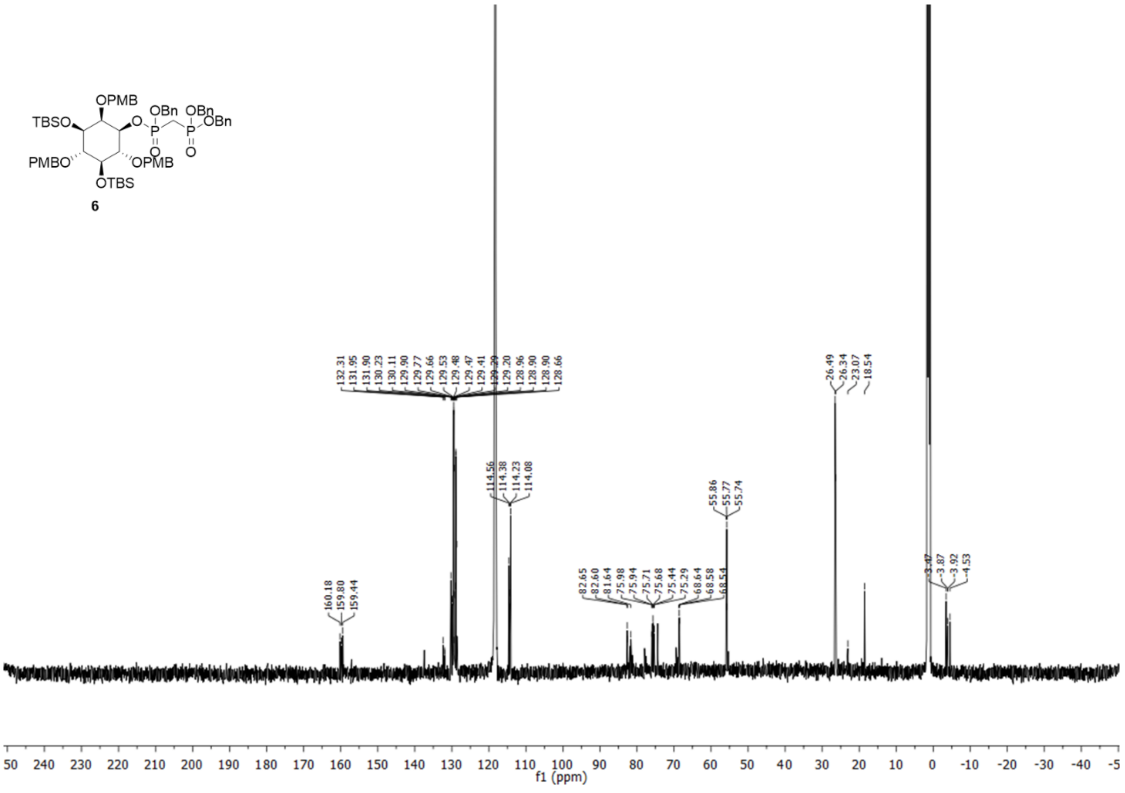

^{13}C spectrum of compound **6**.

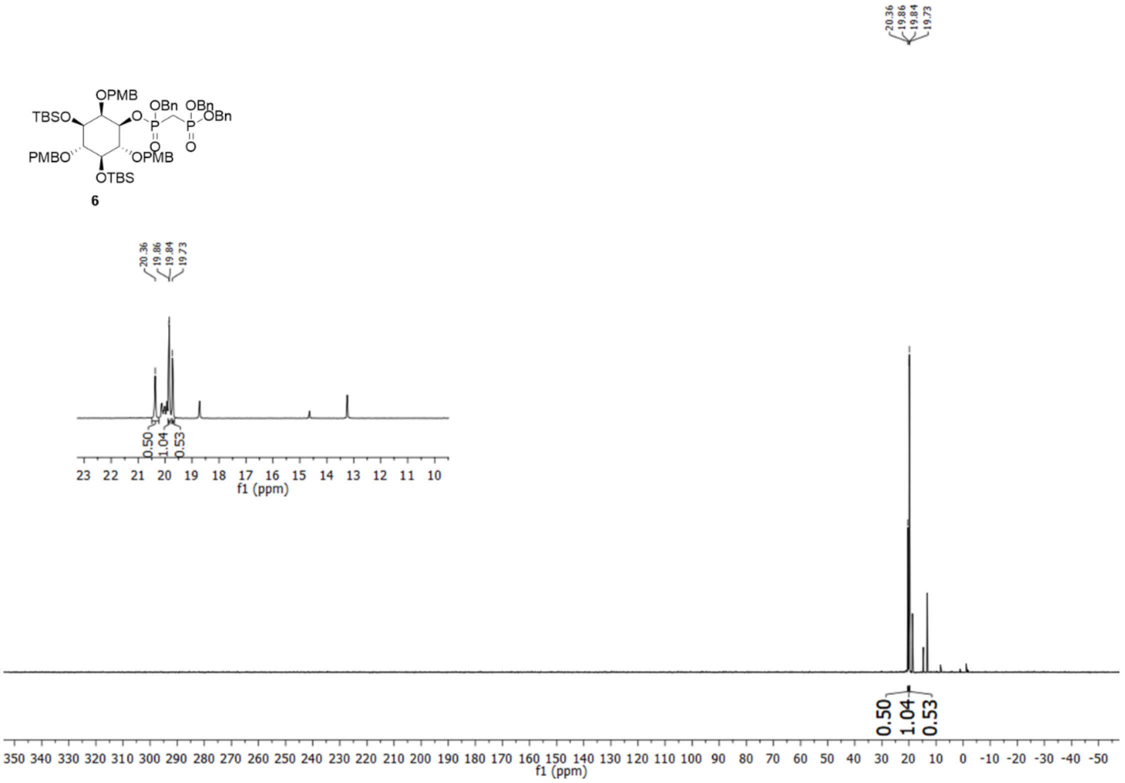

^{31}P spectrum of compound **6**.

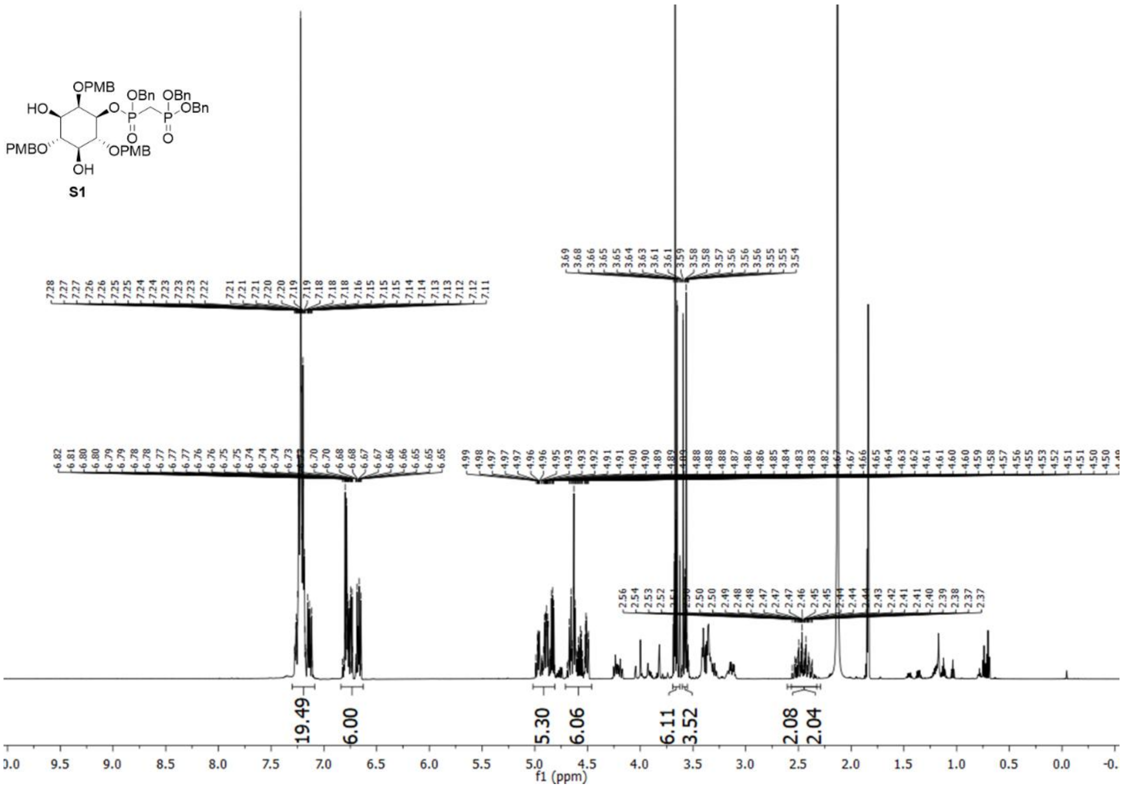

^{1}H spectrum of compound **S1**.

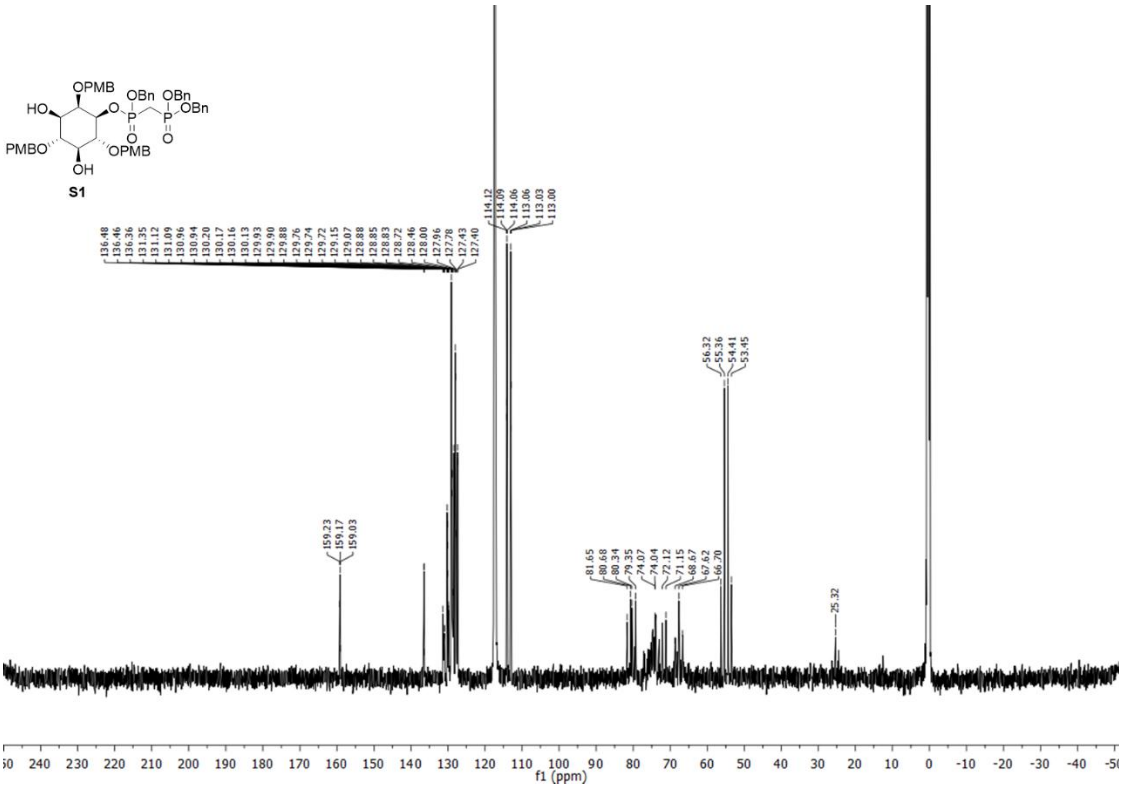

^{13}C spectrum of compound **S1**.

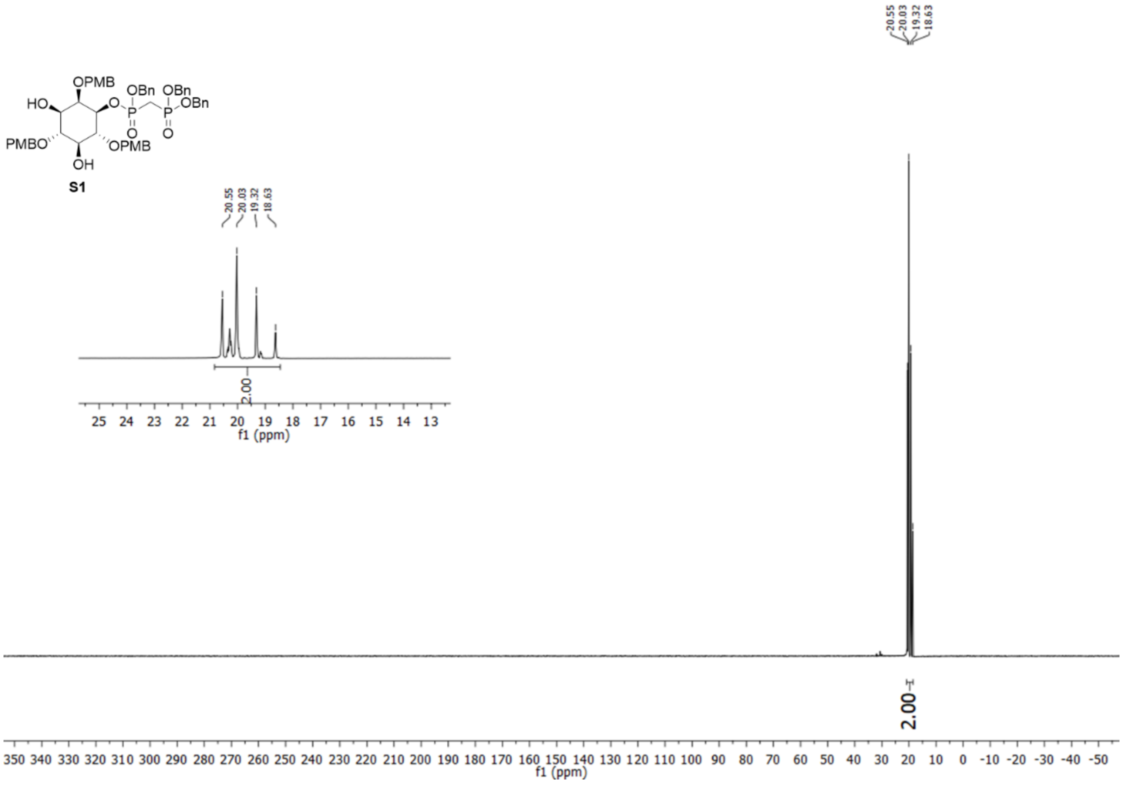

^{31}P spectrum of compound **S1**.

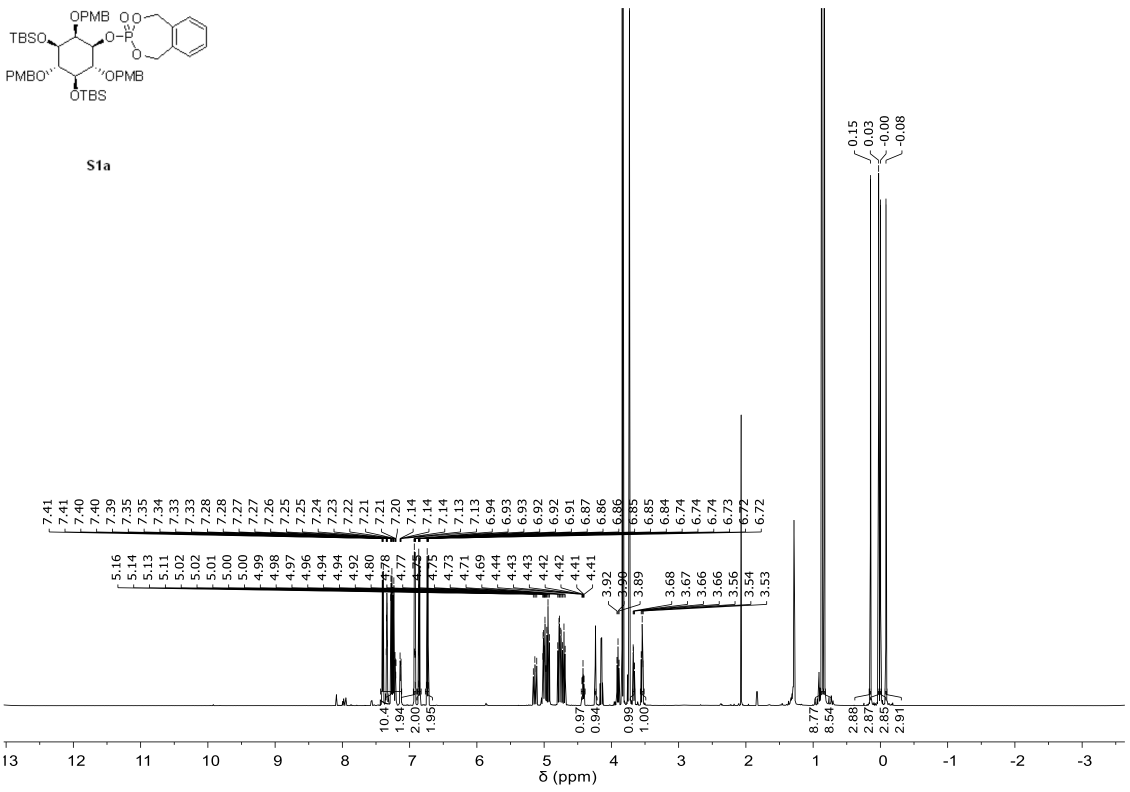

^{1}H spectrum of compound **S1a**.

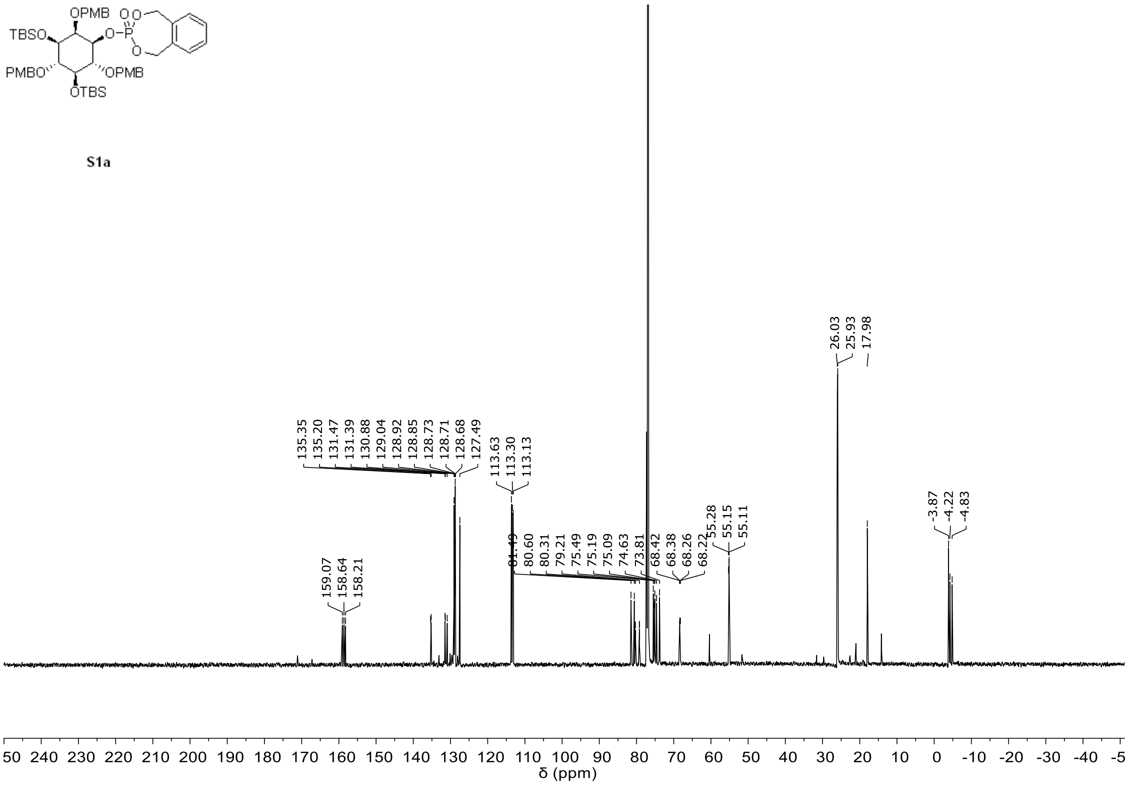

^{13}C spectrum of compound **S1a**.

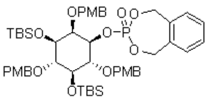

S1a

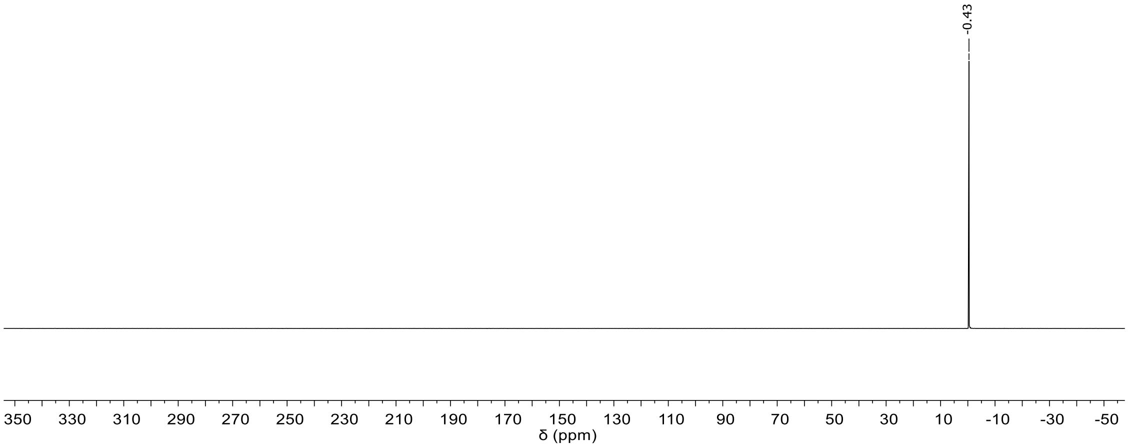

^{31}P spectrum of compound **S1a**.

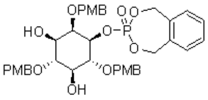

S2a

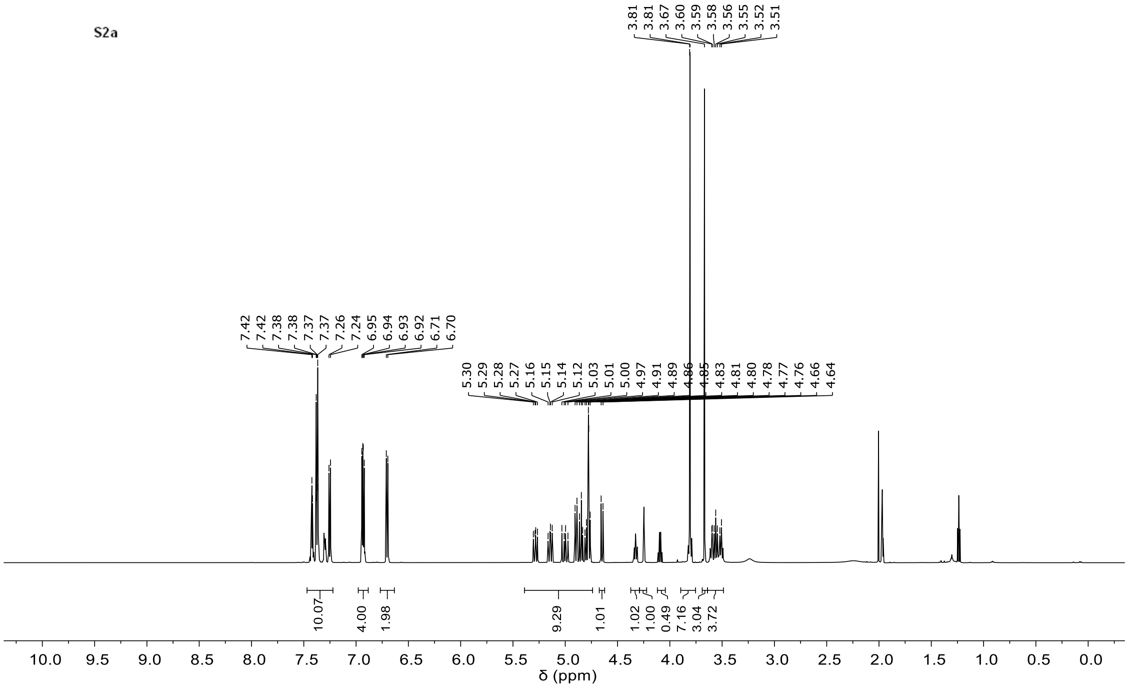

^{1}H spectrum of compound **S2a**.

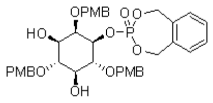

S2a

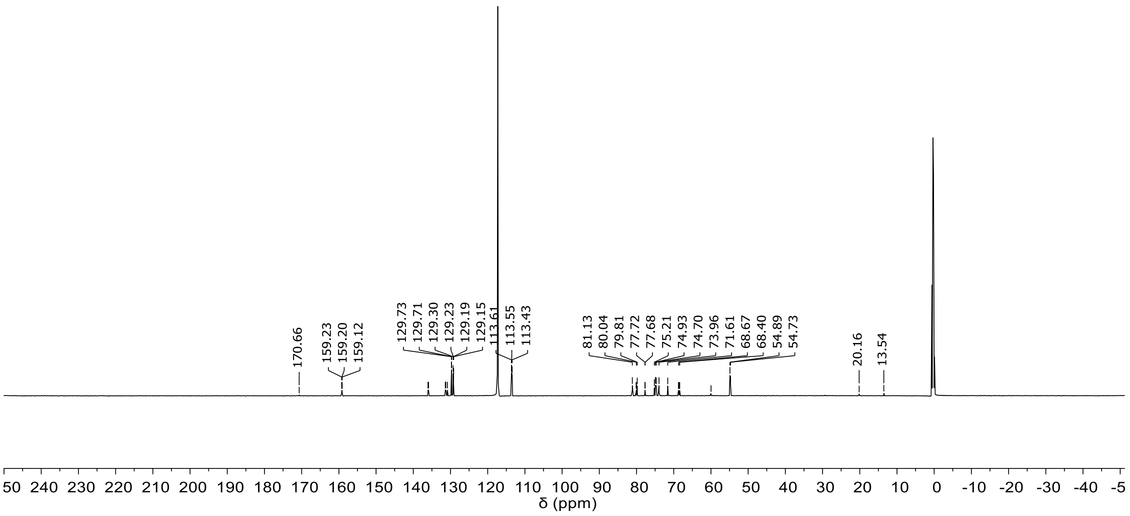

^{13}C spectrum of compound **S2a**.

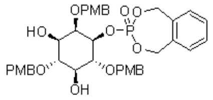

S2a

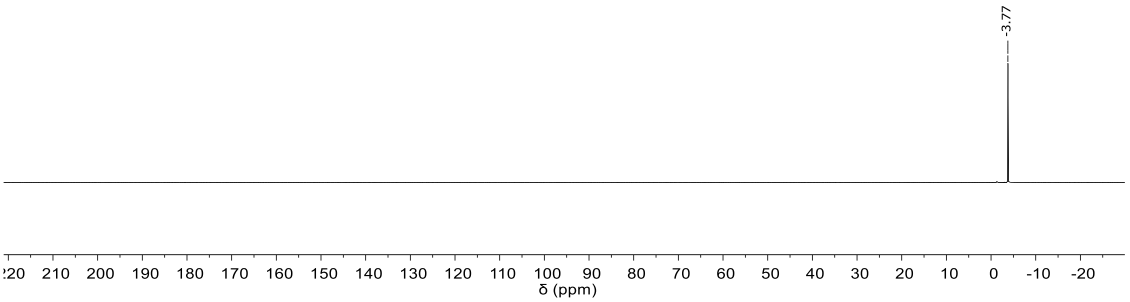

^{31}P spectrum of compound **S2a**.

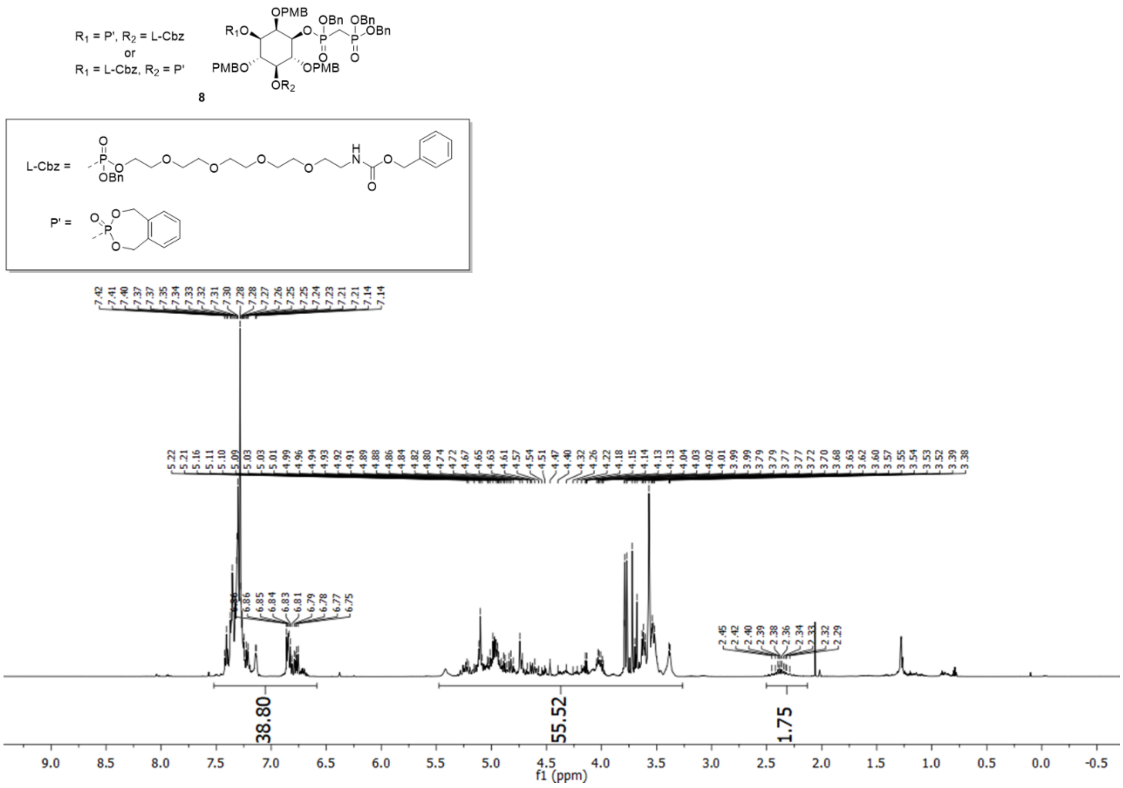

^{1}H spectrum of compound **8**.

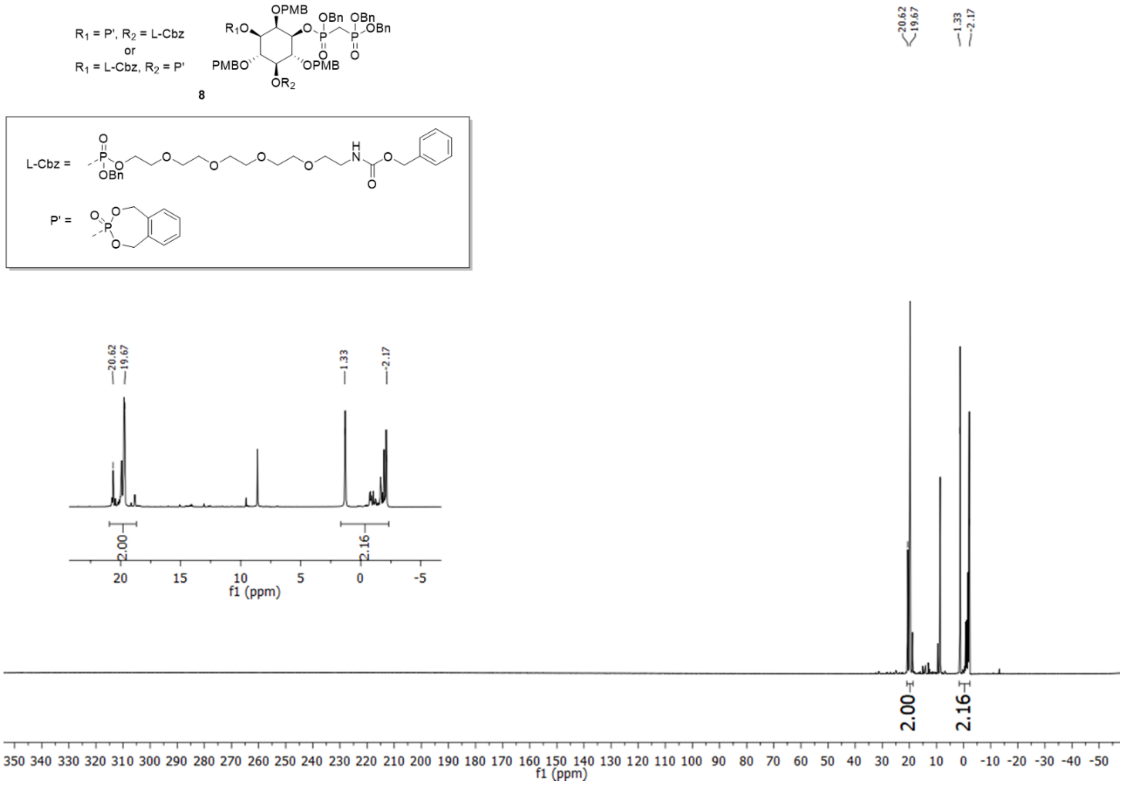

^{31}P spectrum of compound **8**.

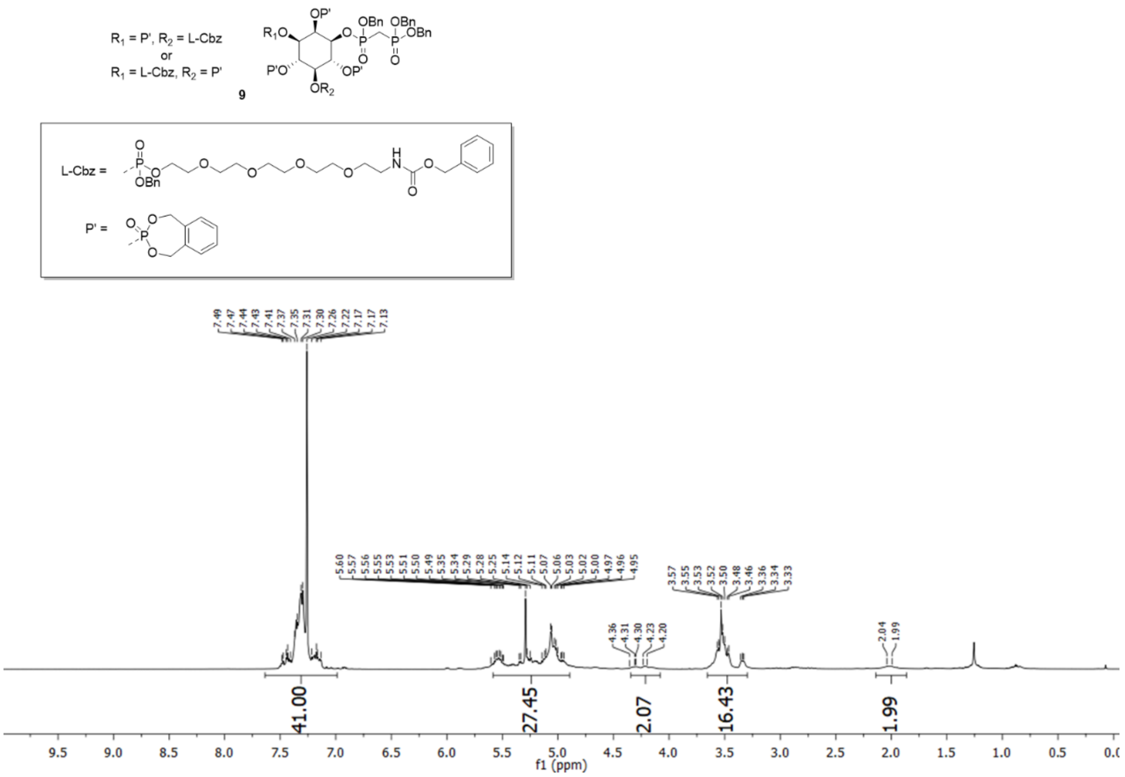

[1] H spectrum of compound **9**.

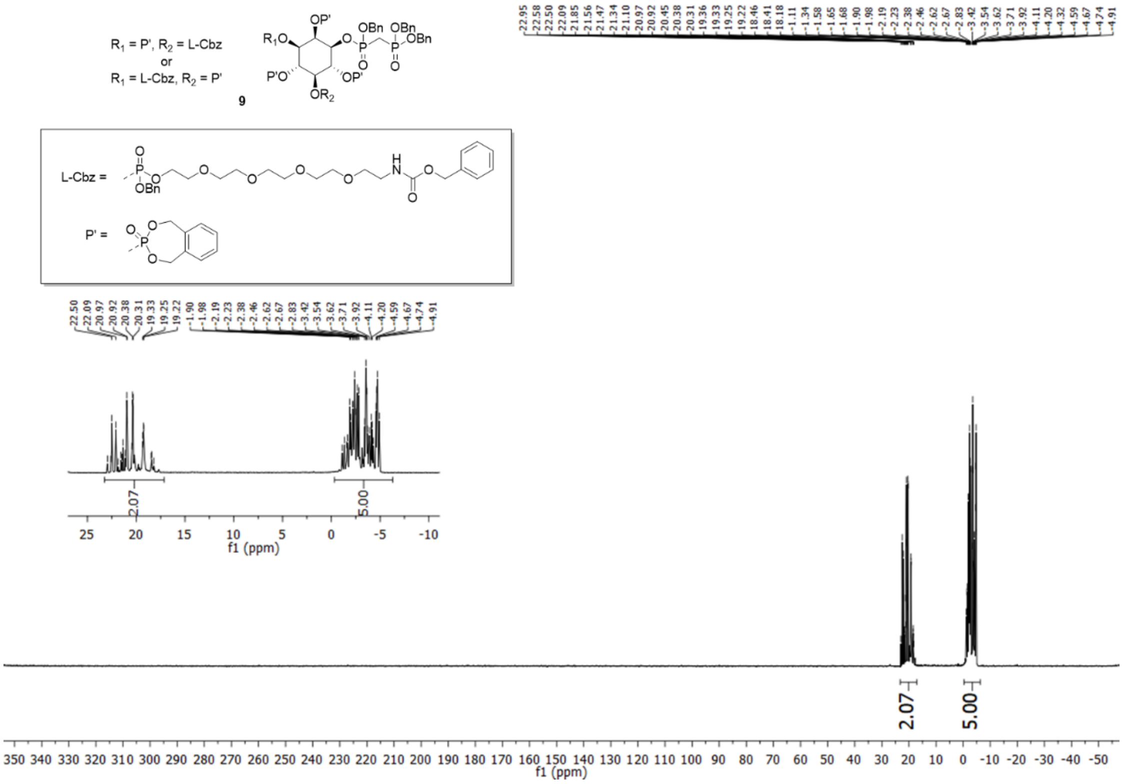

[31] P spectrum of compound **9**.

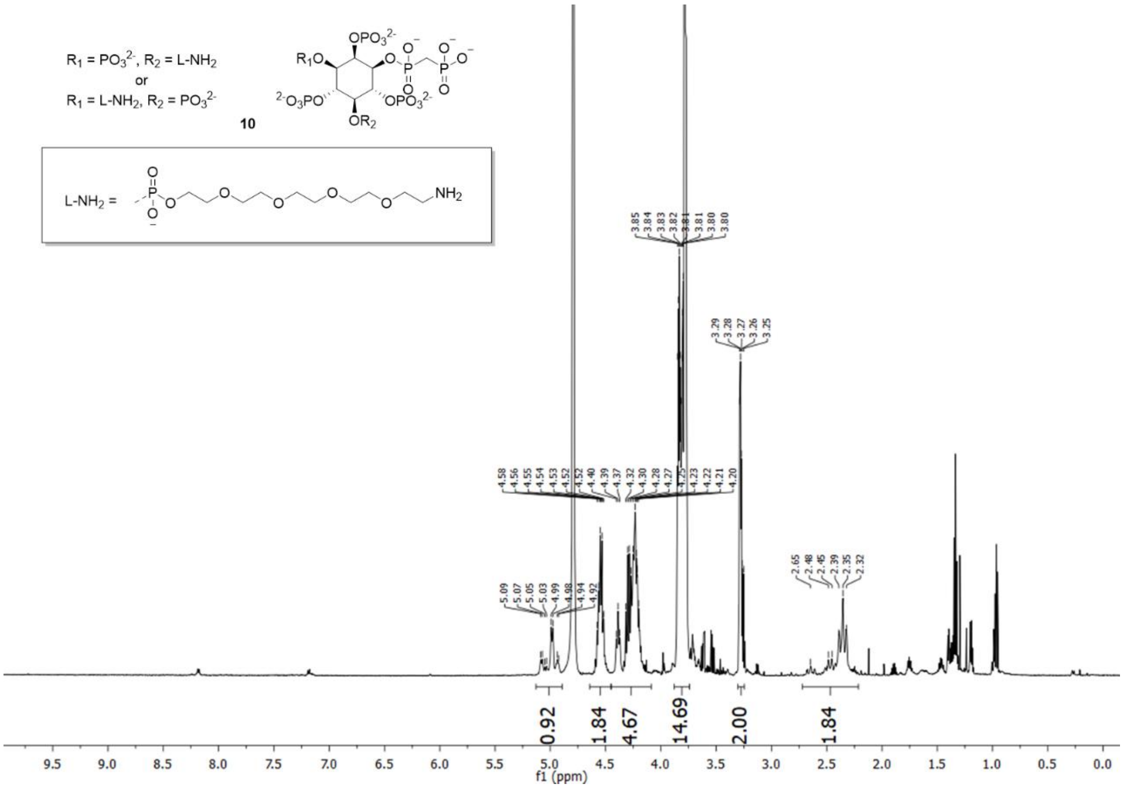

^{1}H spectrum of compound **10**.

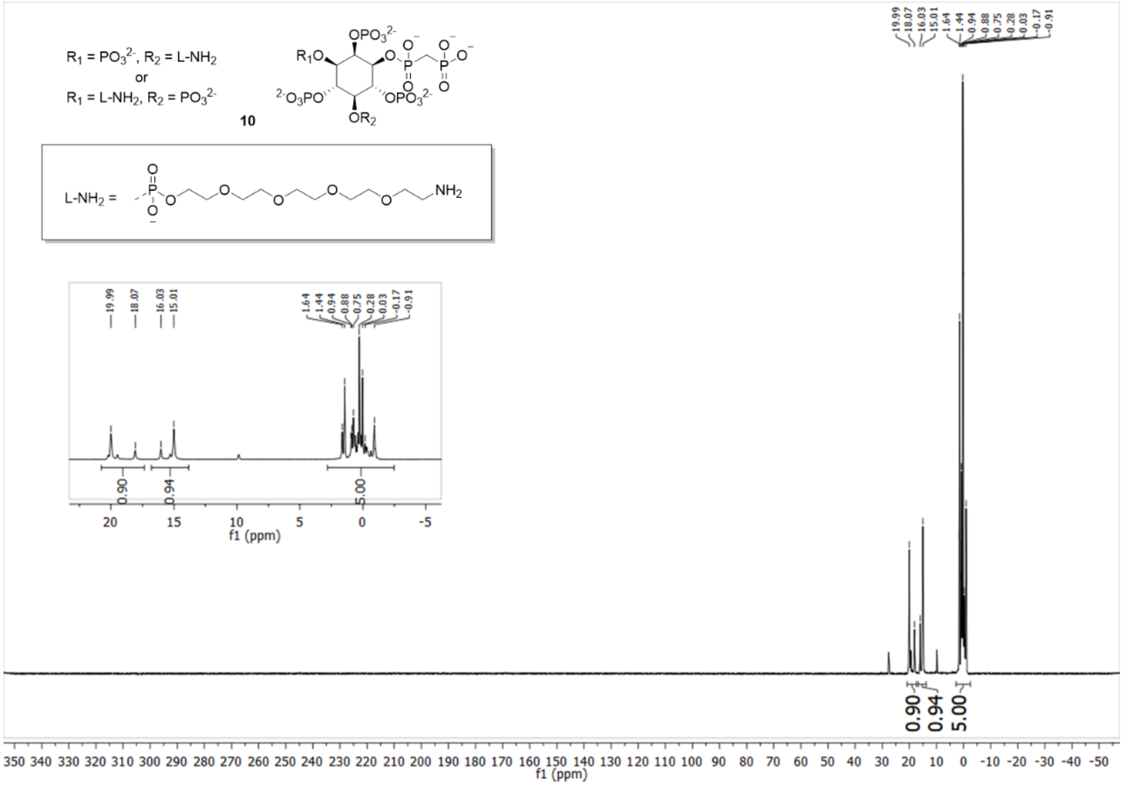

^{31}P spectrum of compound **10**.

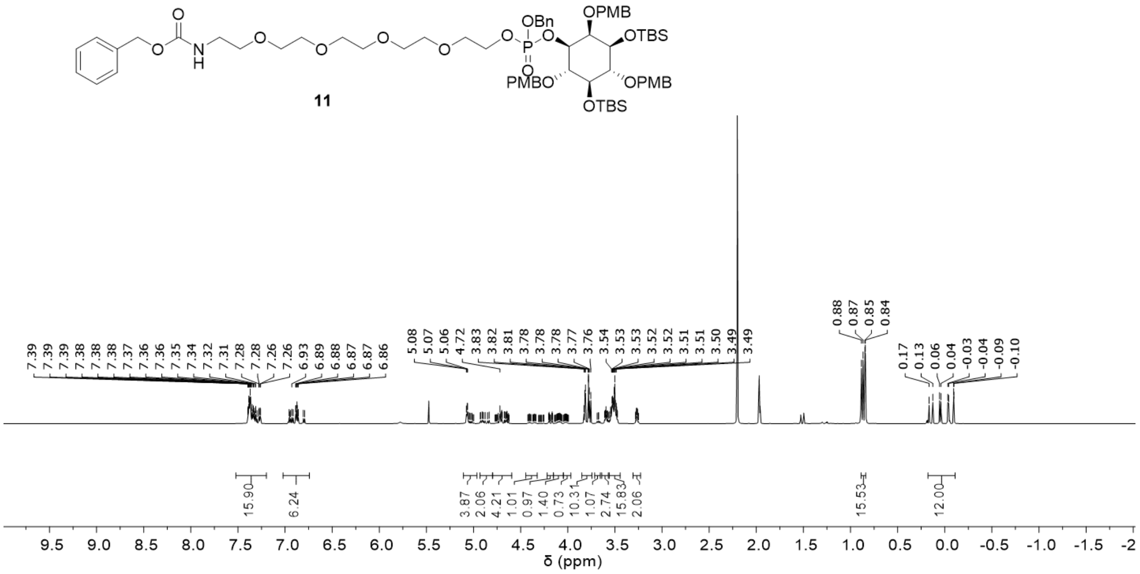

^{1}H spectrum of compound **11**.

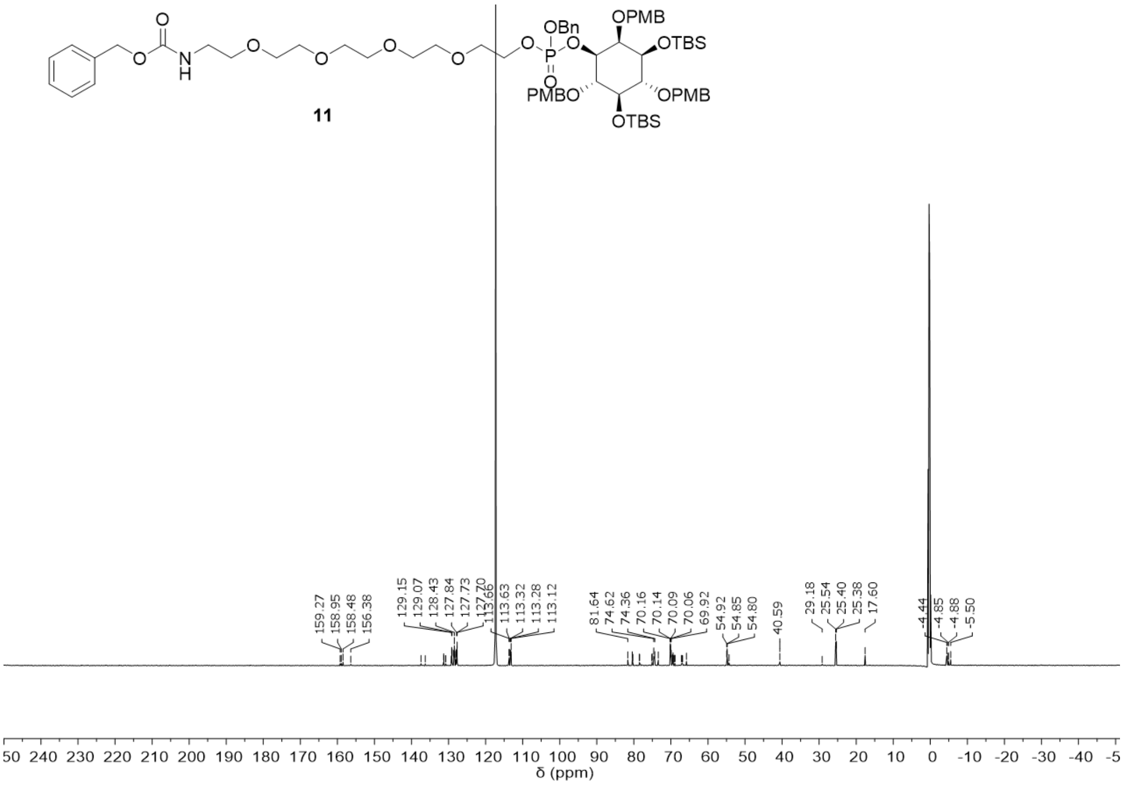

^{13}C spectrum of compound **11**.

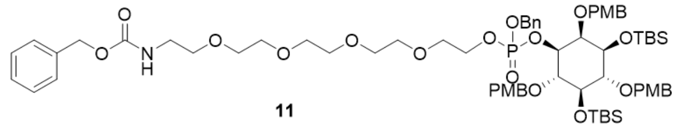

11

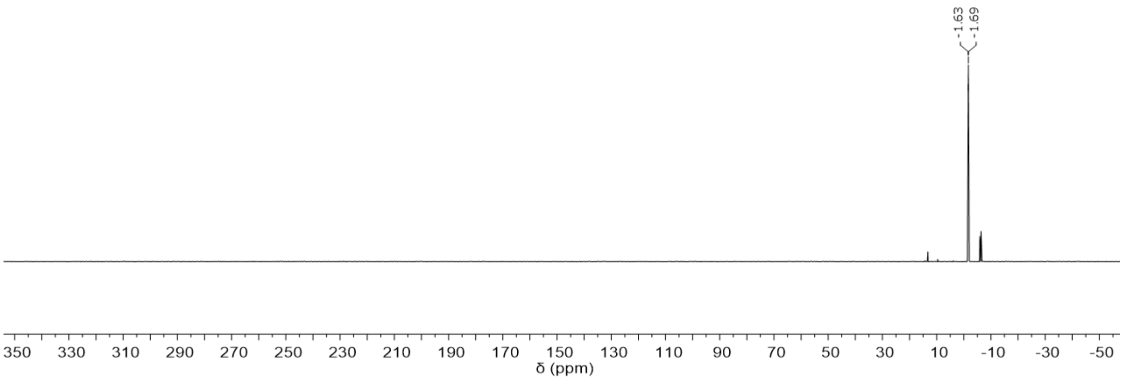

^{31}P spectrum of compound **11**.

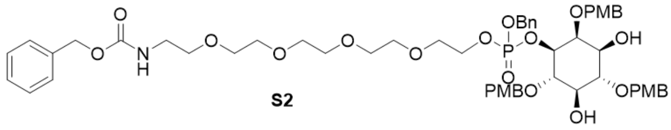

S2

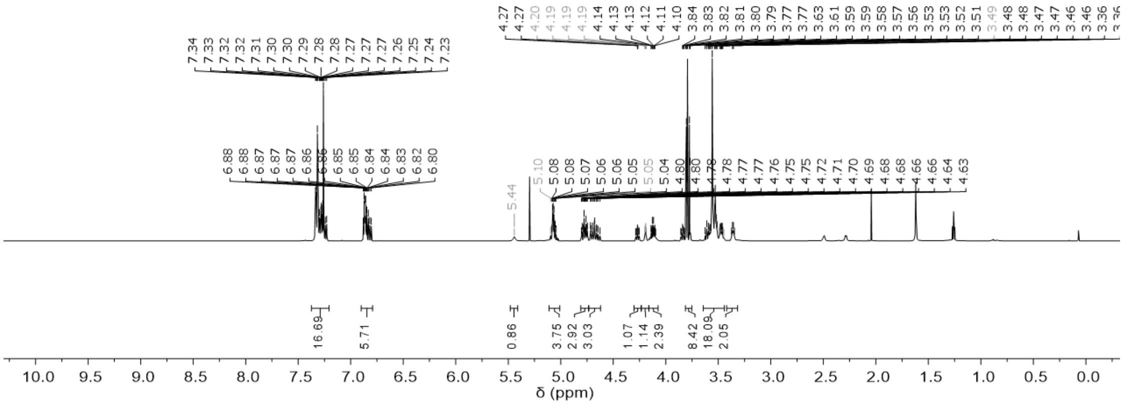

^{1}H spectrum of compound **S2**.

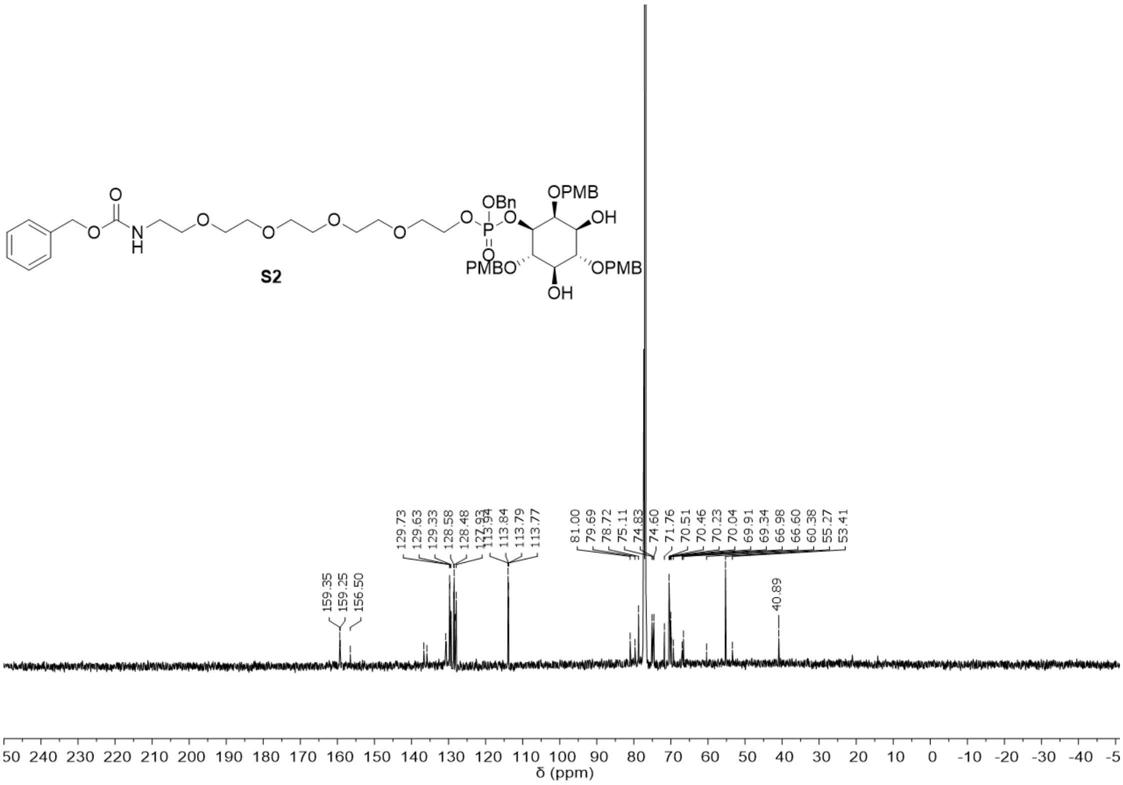

^{13}C spectrum of compound **S2**.

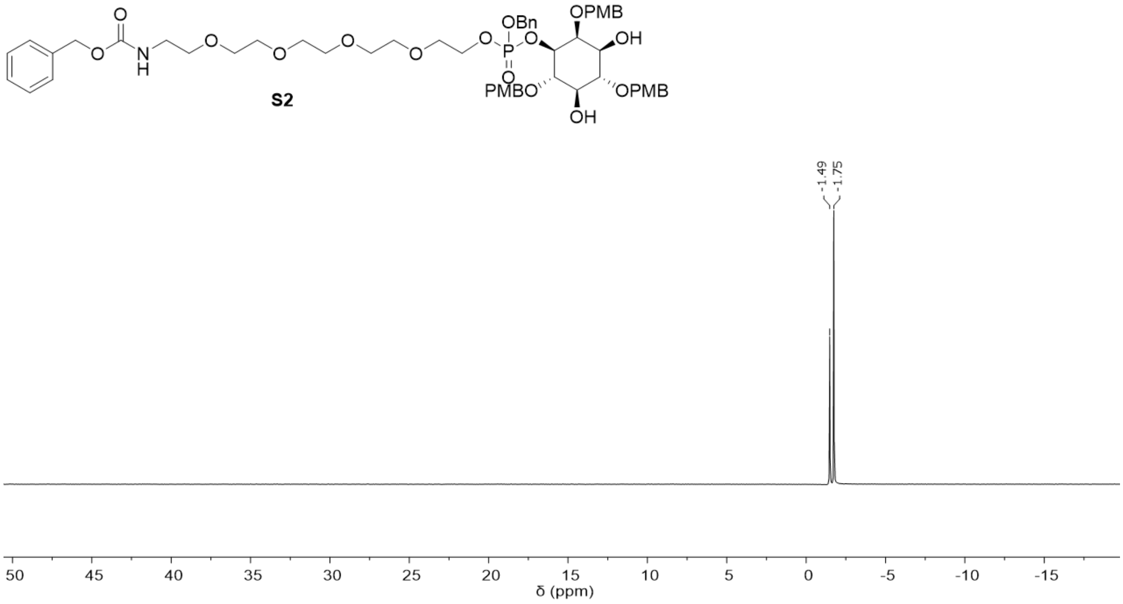

^{31}P spectrum of compound **S2**.

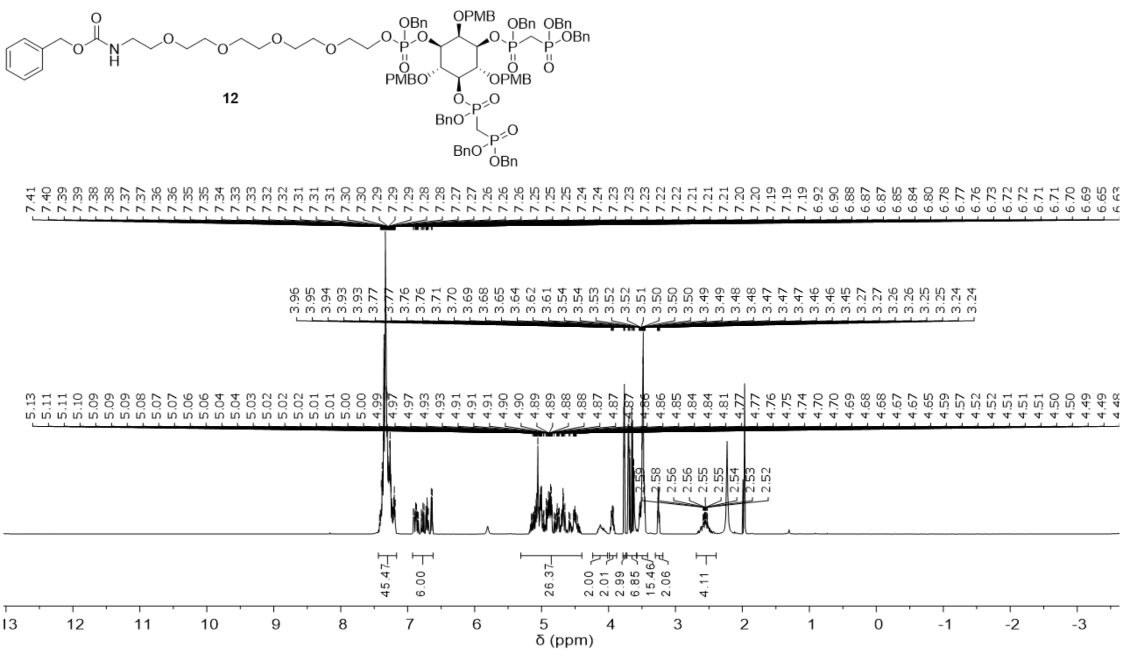

^{1}H spectrum of compound **12**.

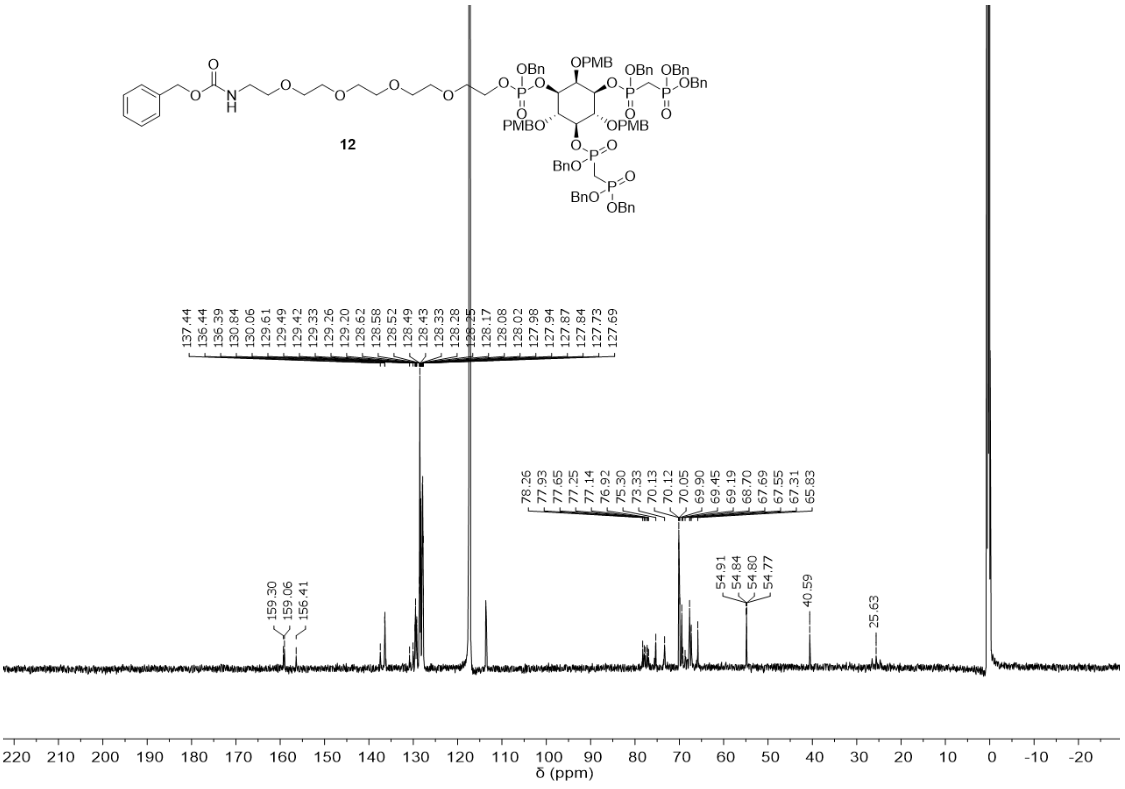

^{13}C spectrum of compound **12**.

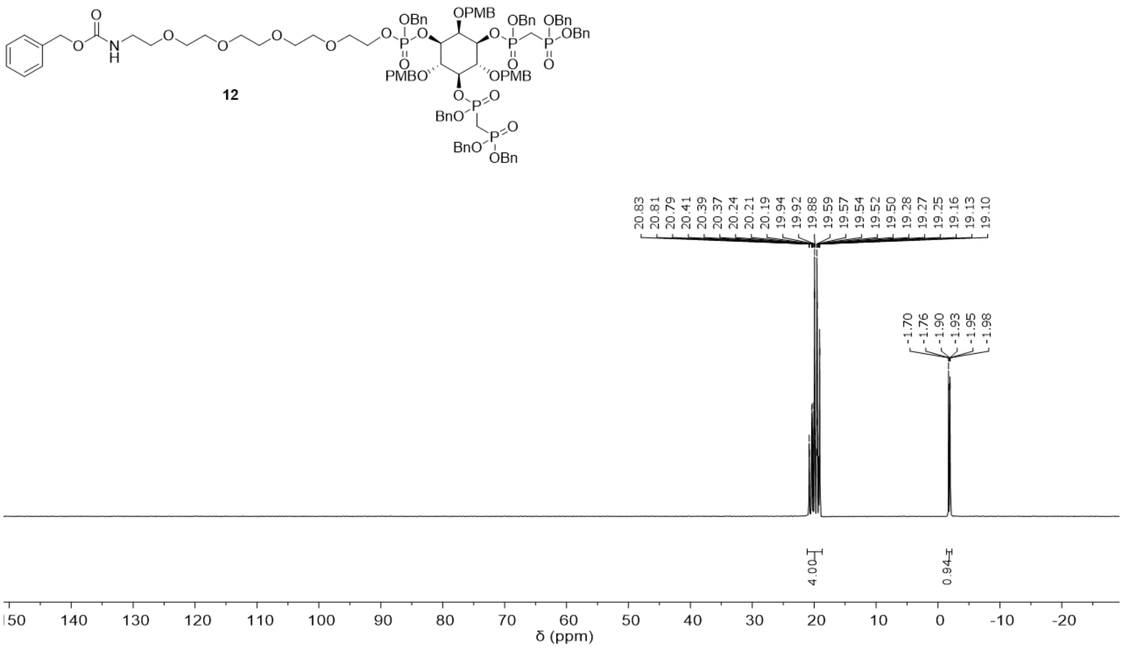

^{31}P spectrum of compound **12**.

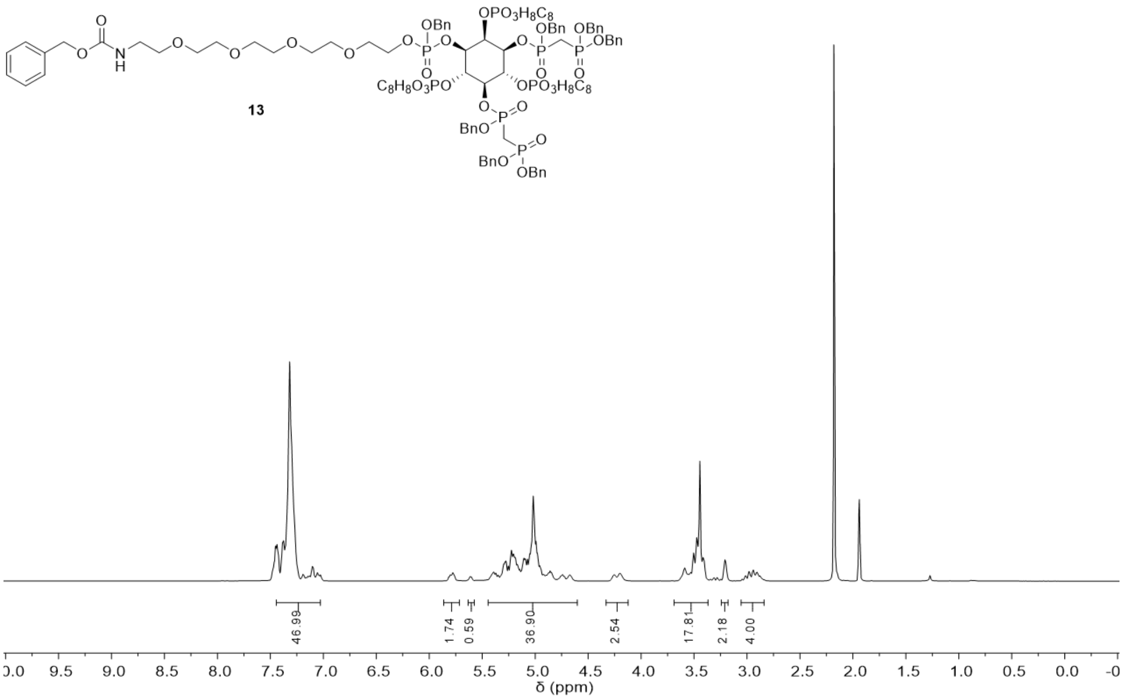

^{1}H spectrum of compound **13**.

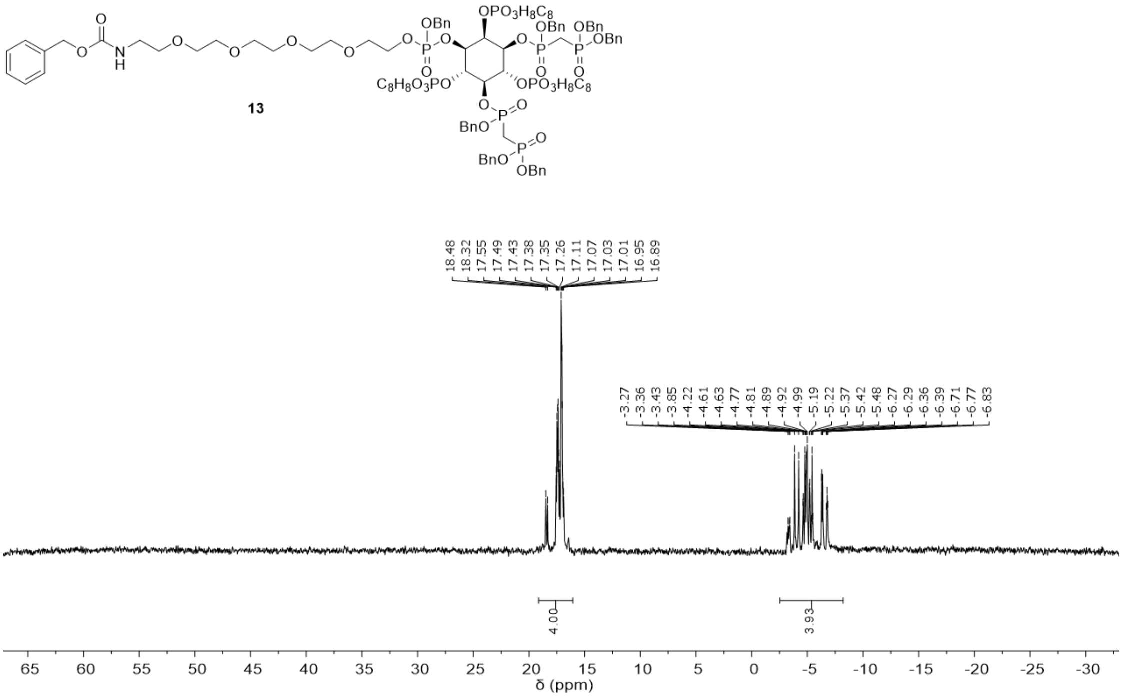

^{31}P spectrum of compound **13**.

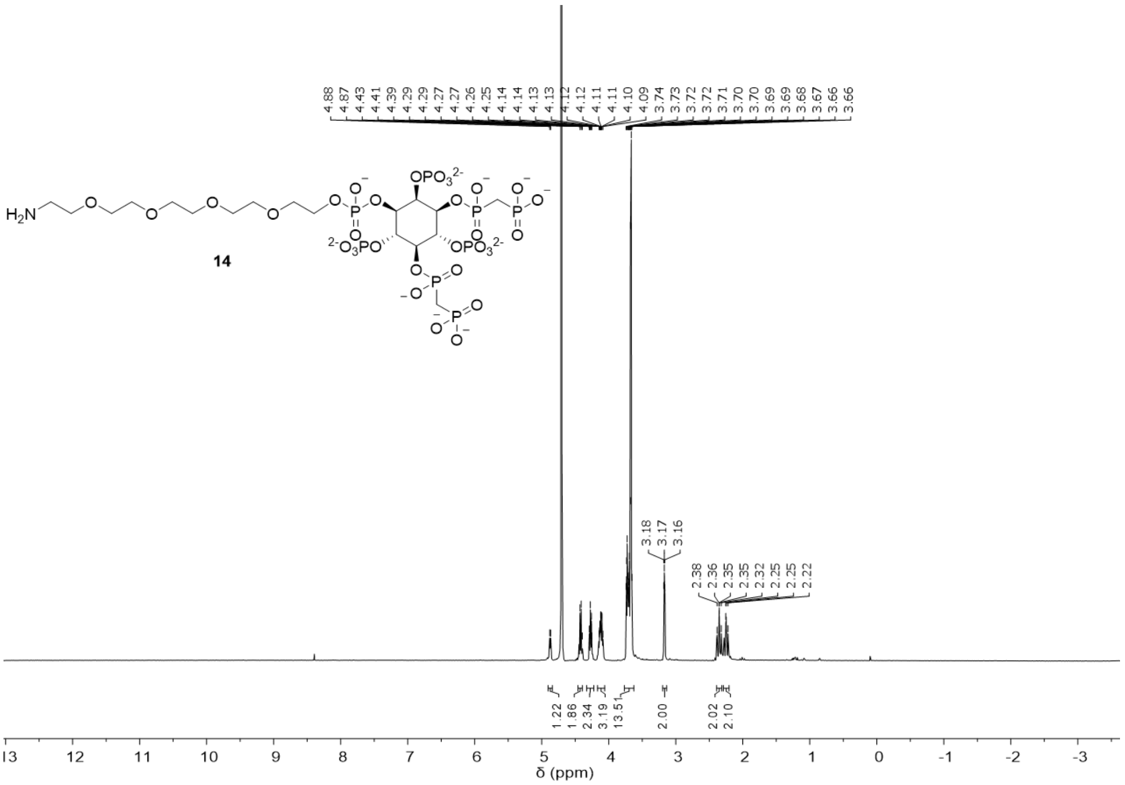

^{1}H spectrum of compound **14**.

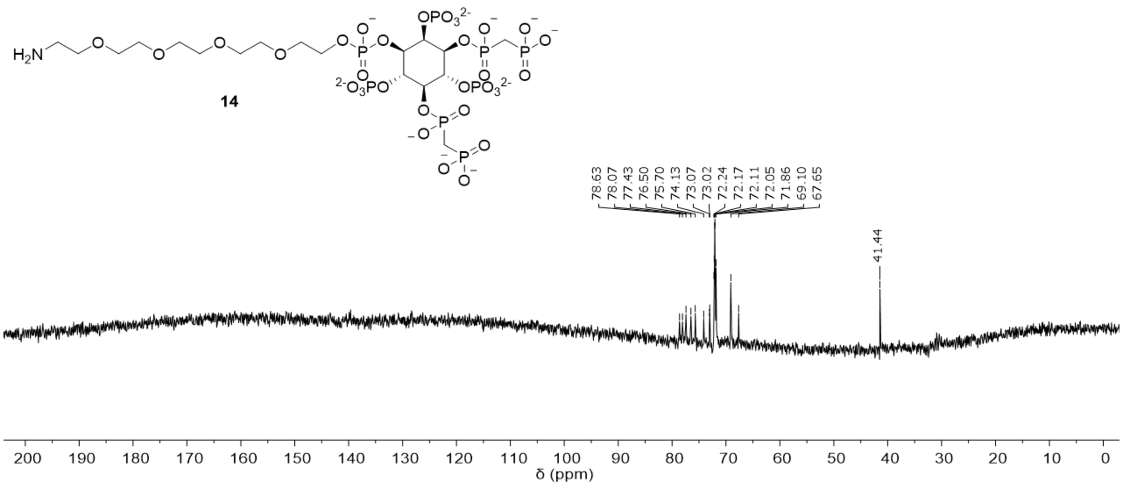

^{13}C spectrum of compound **14**.

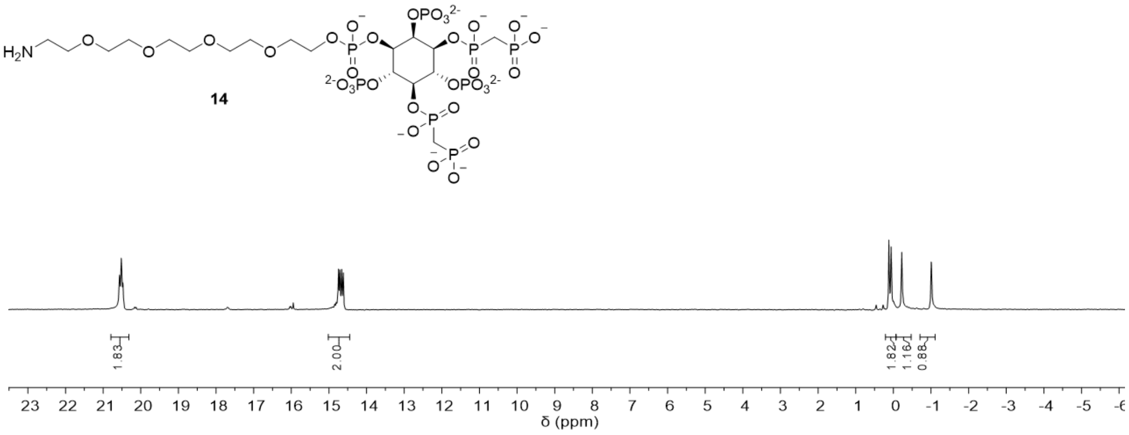

^{31}P spectrum of compound **14**.

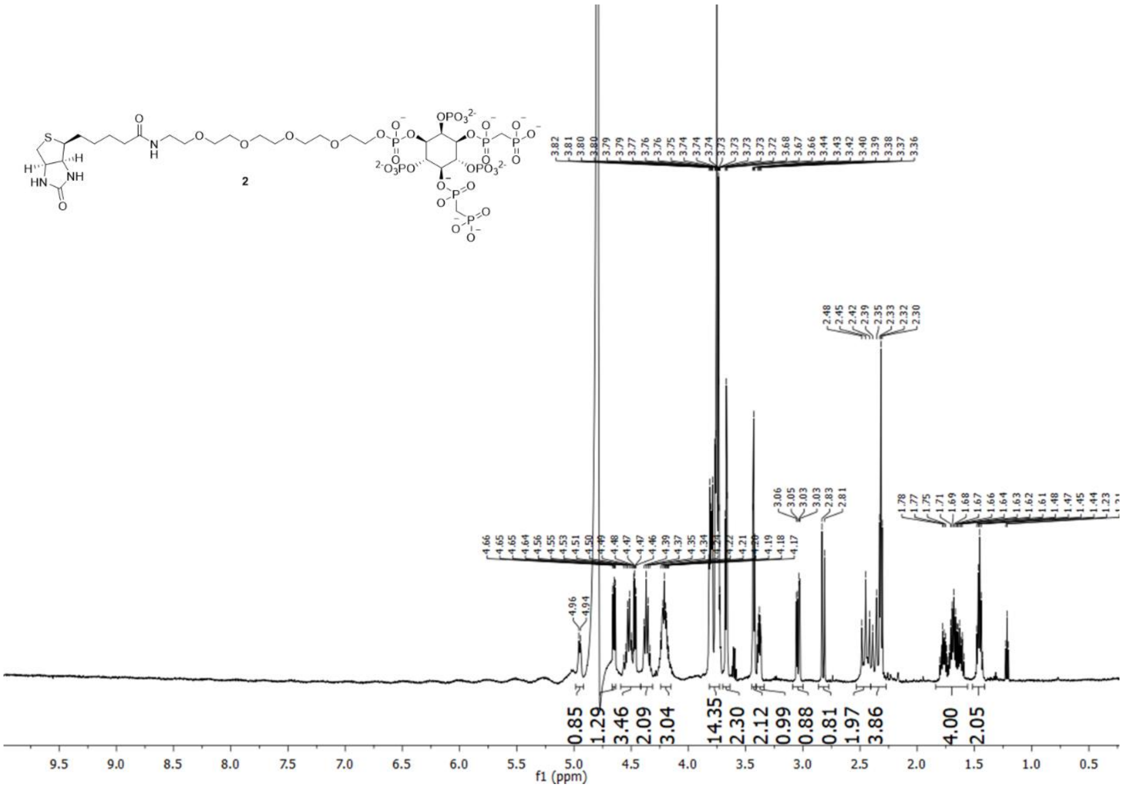

^{1}H spectrum of compound **2**.

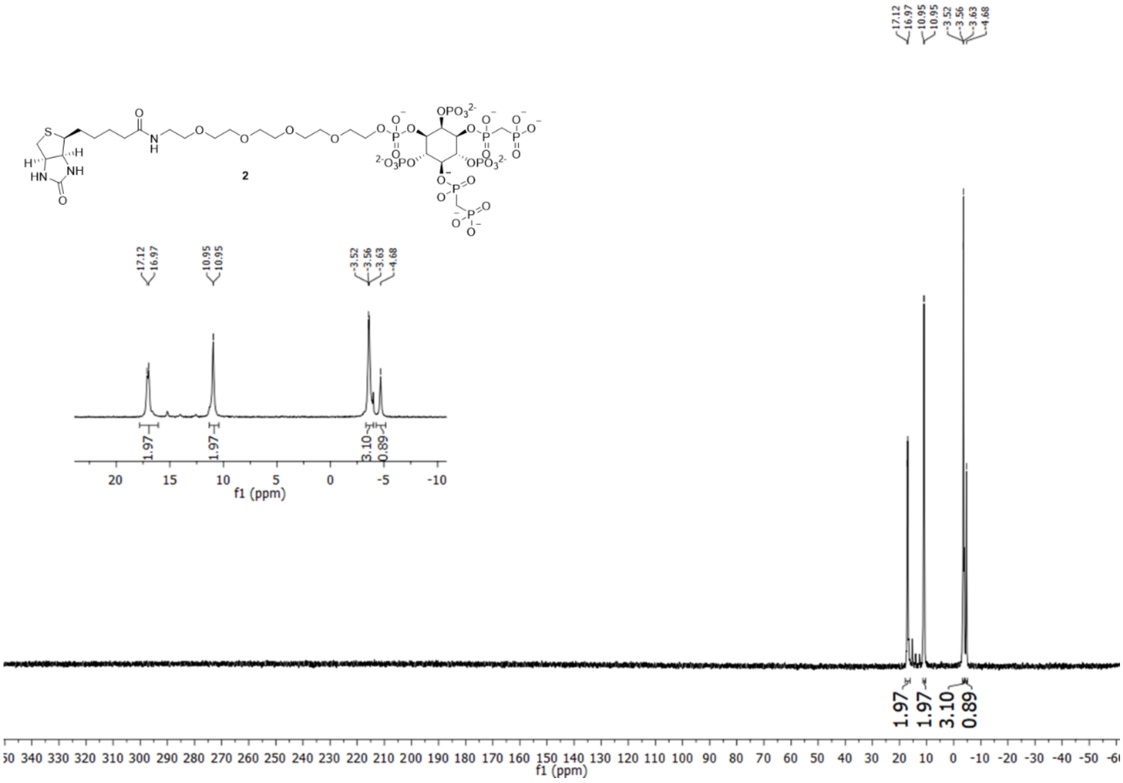

^{31}P spectrum of compound **2**.

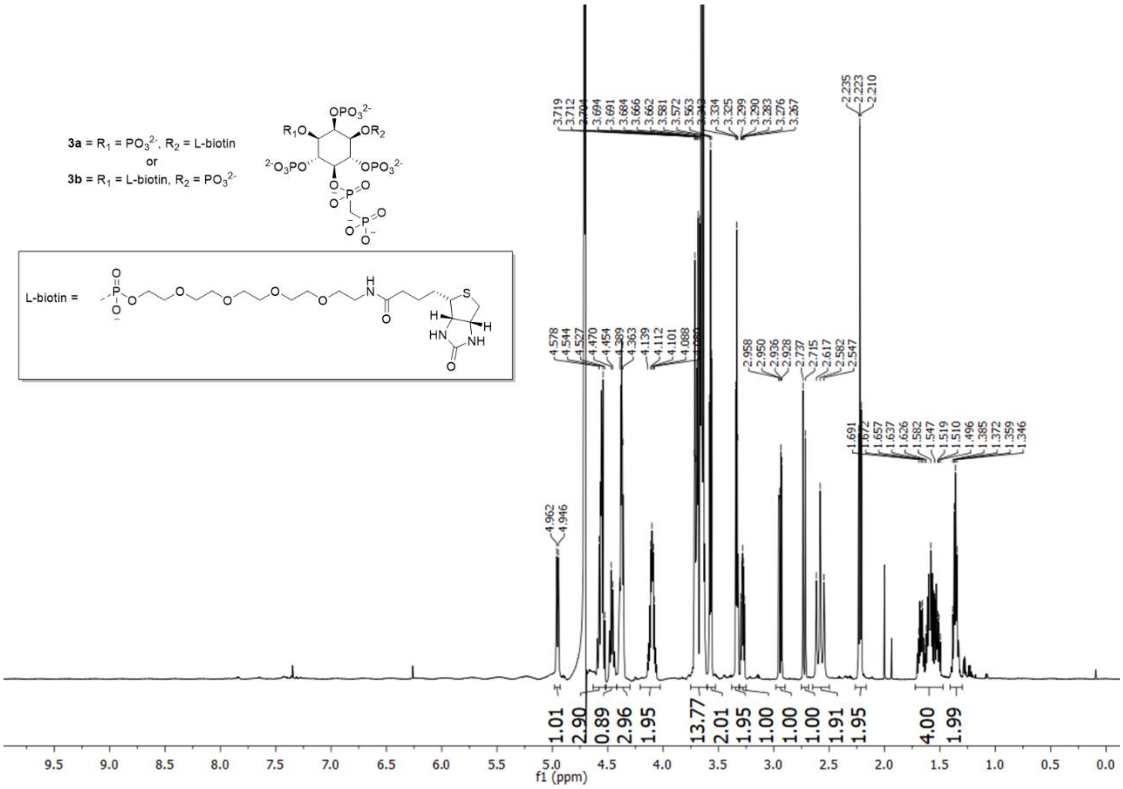

^{1}H spectrum of compound **3**.

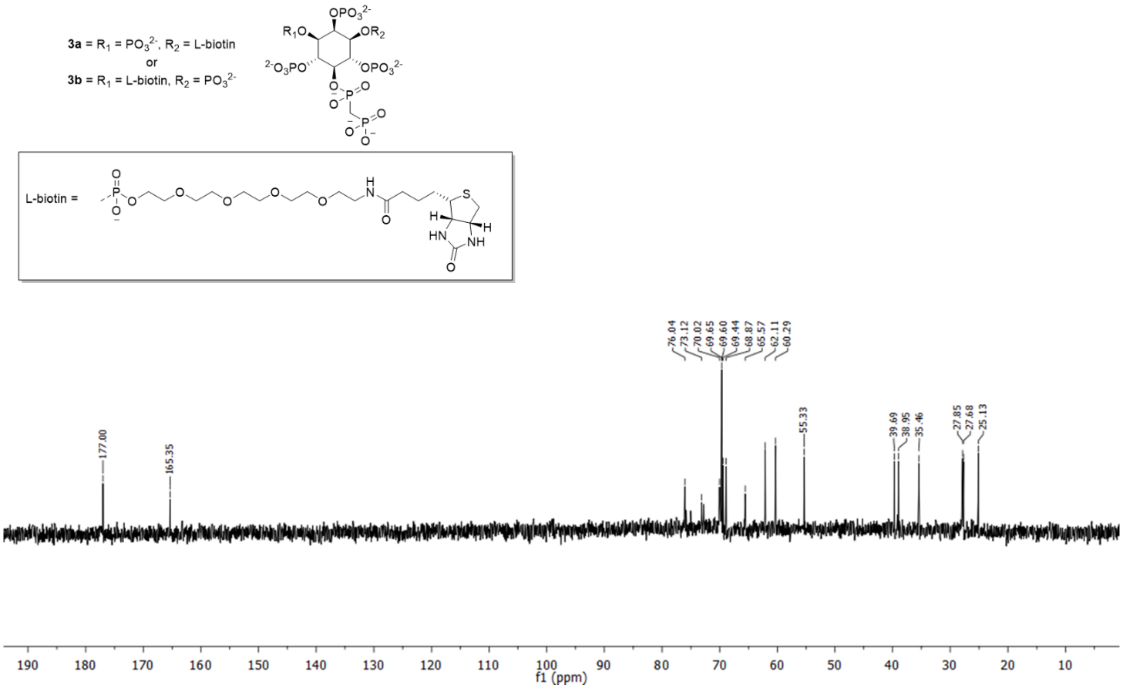

^{13}C spectrum of compound **3**.

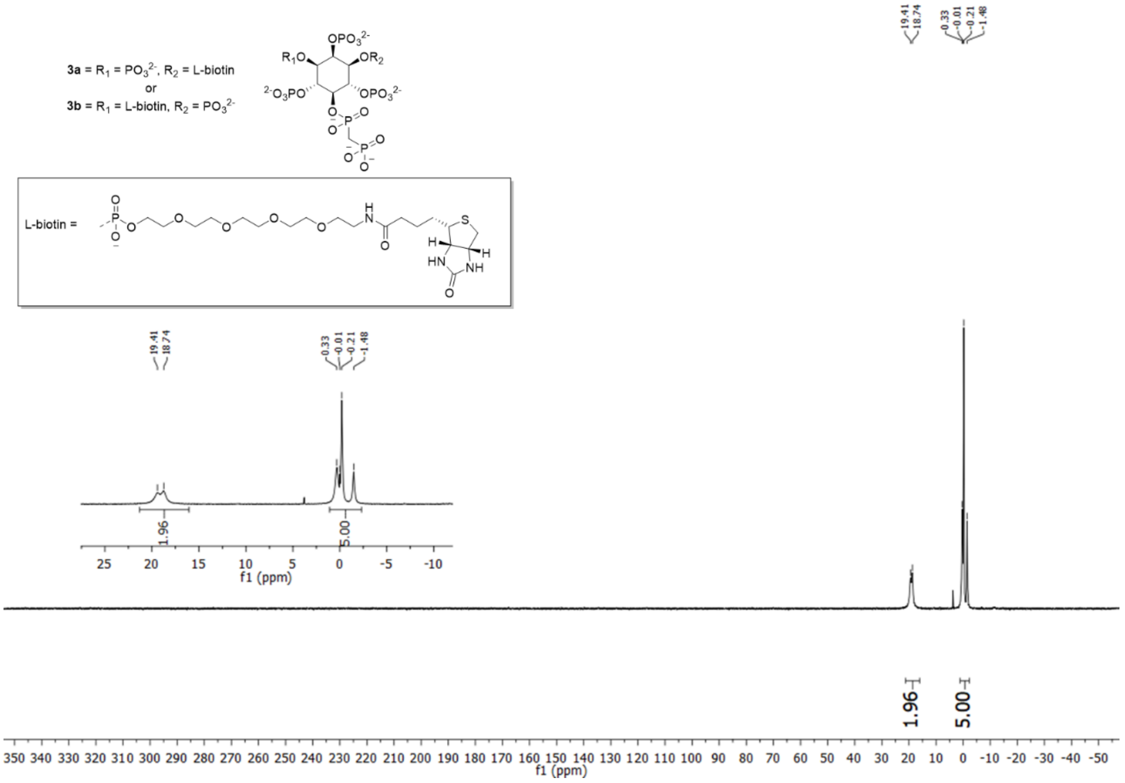

^{31}P spectrum of compound **3**.

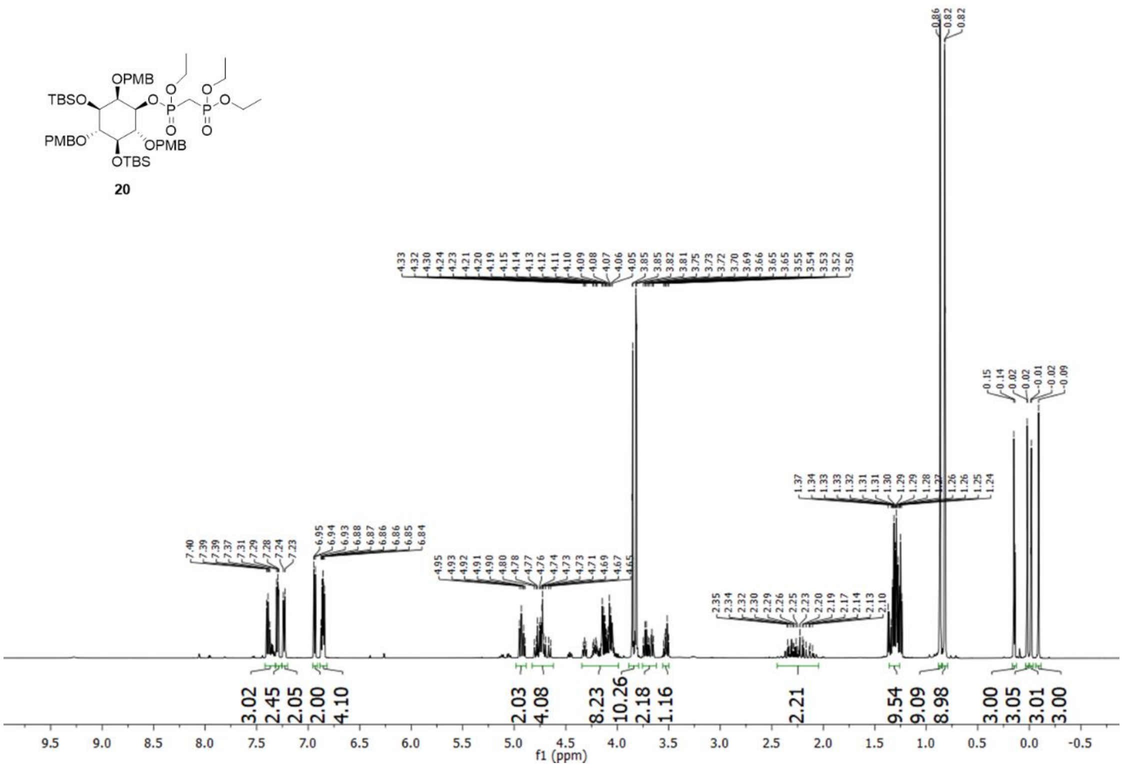

^{1}H spectrum of compound **20**.

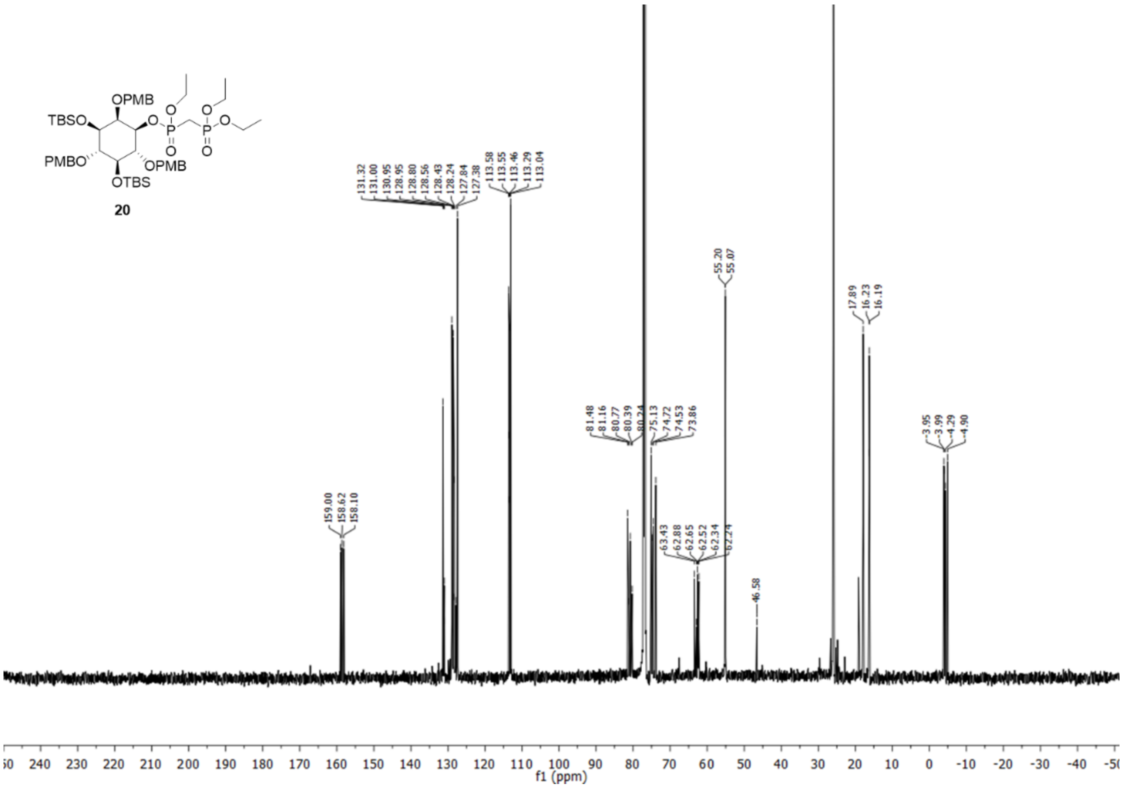

^{13}C spectrum of compound **20**.

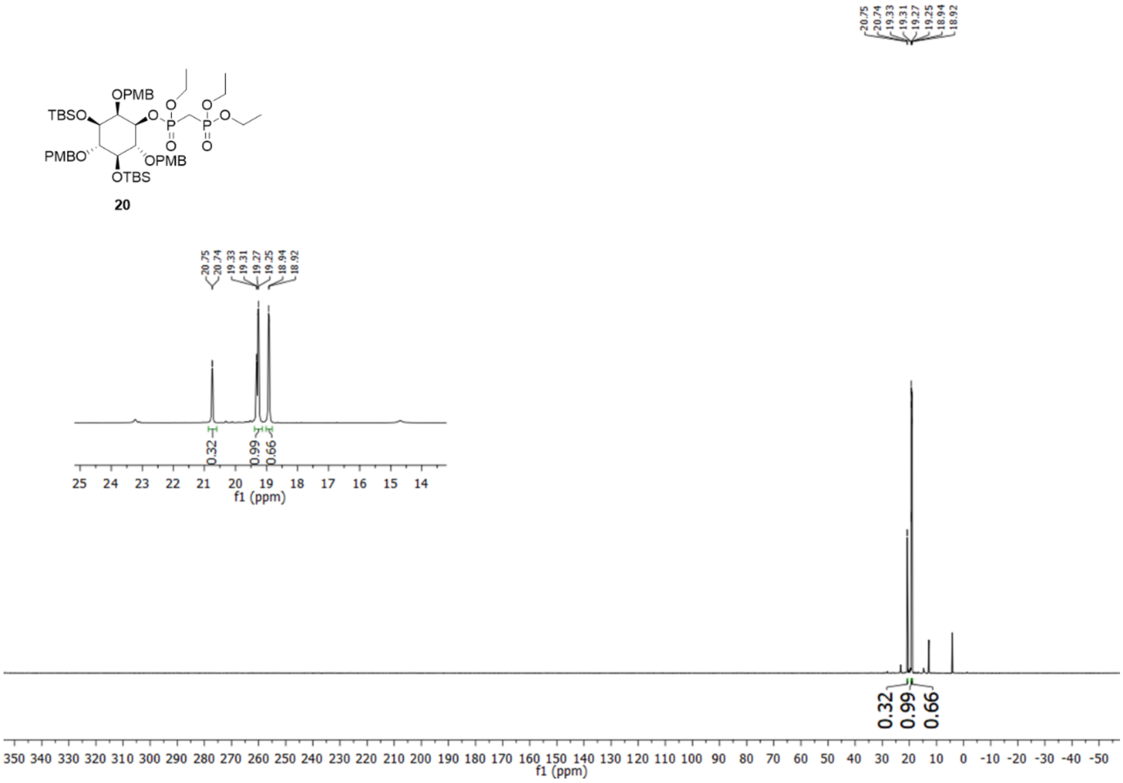

^{31}P spectrum of compound **20**.

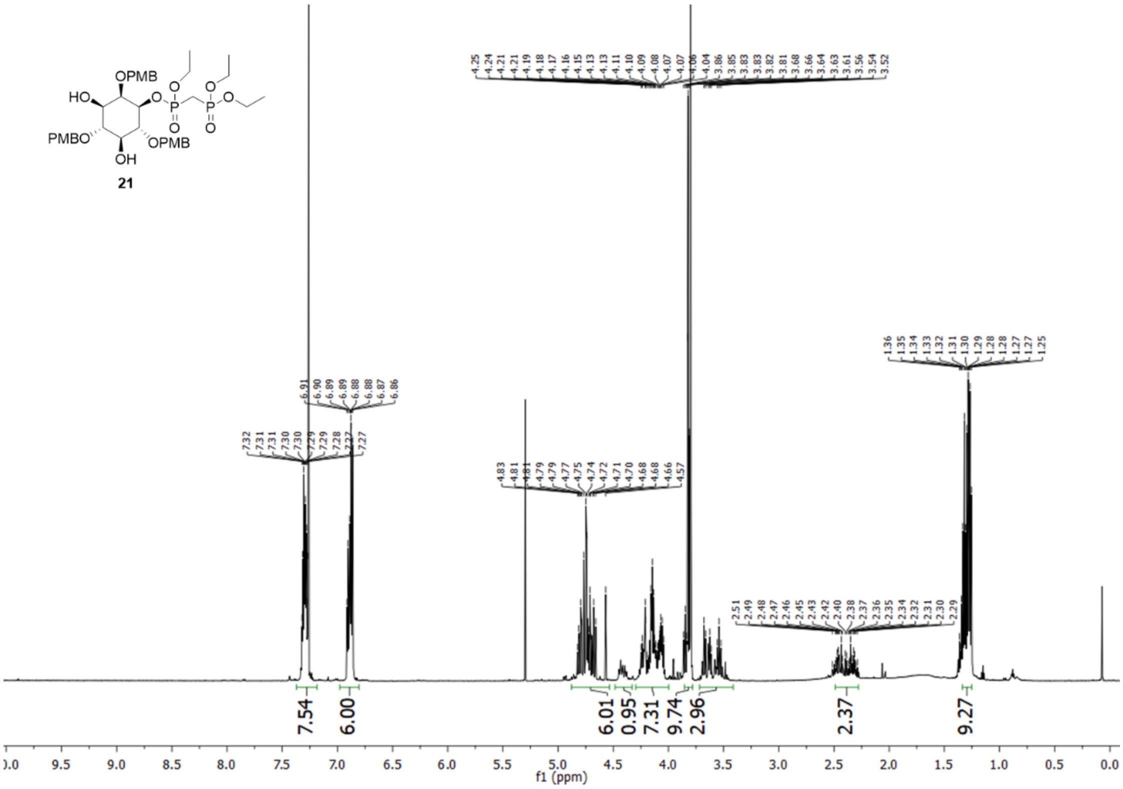

^{1}H spectrum of compound **21**.

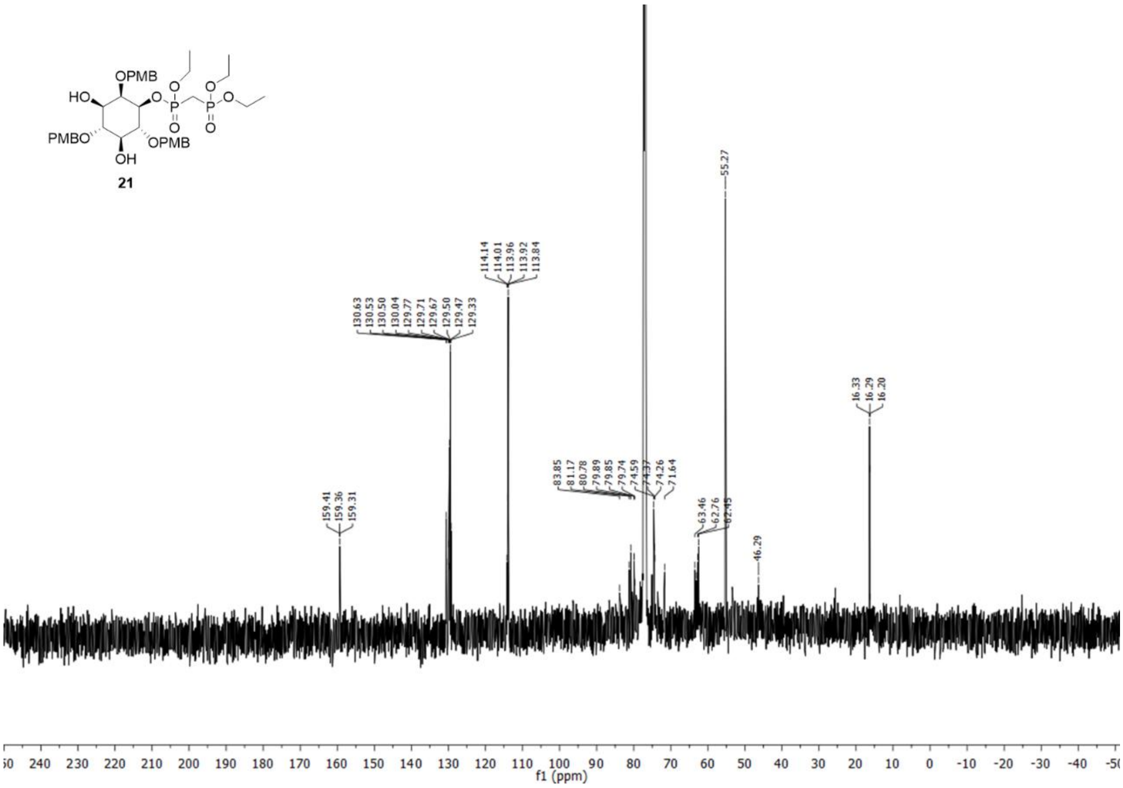

^{13}C spectrum of compound **21**.

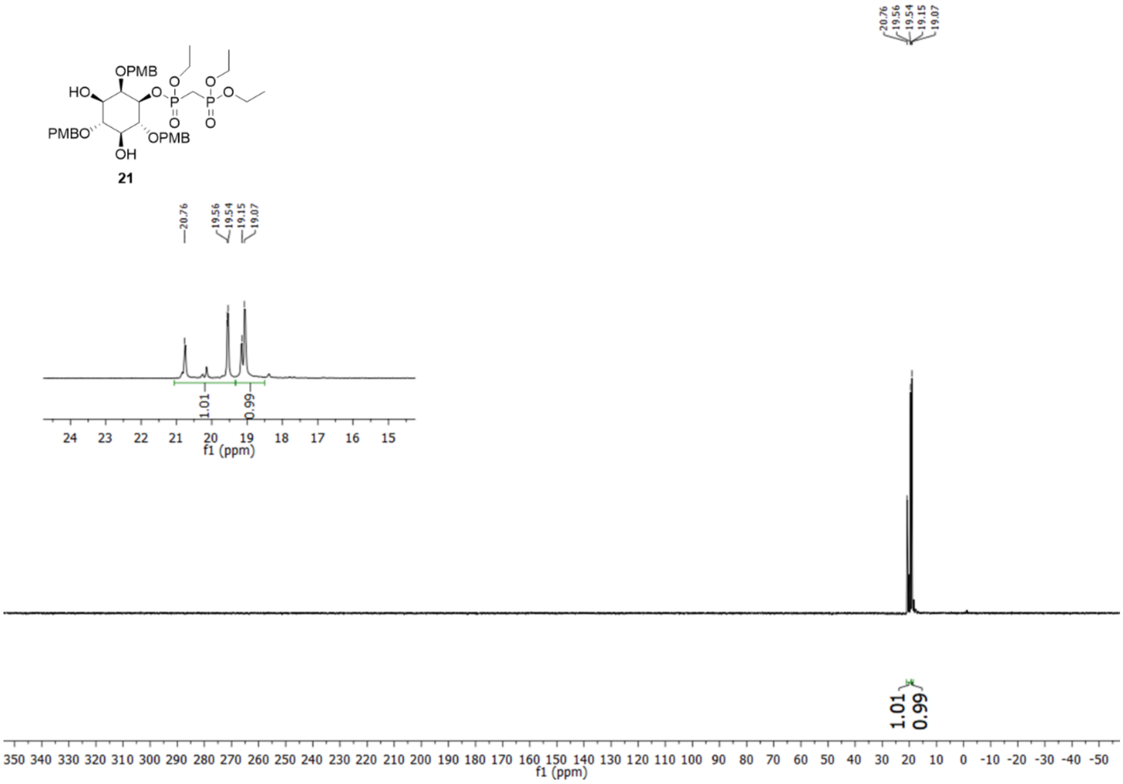

^{31}P spectrum of compound **21**.

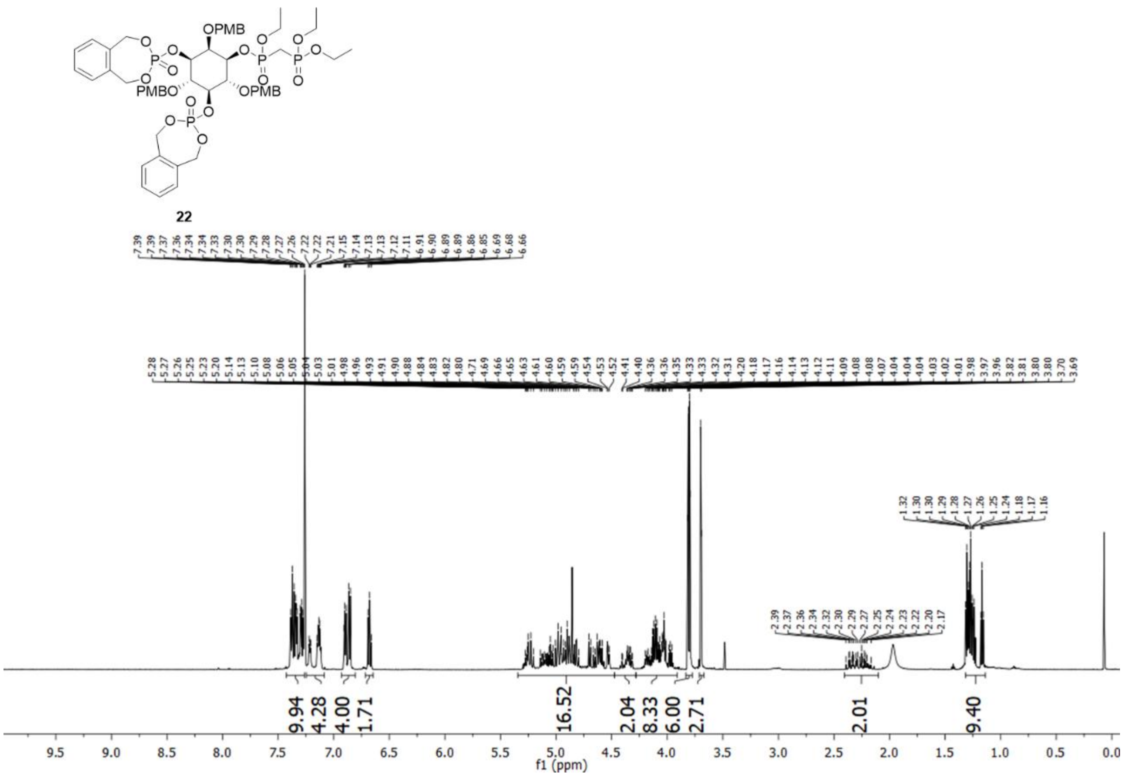

^{1}H spectrum of compound **22**.

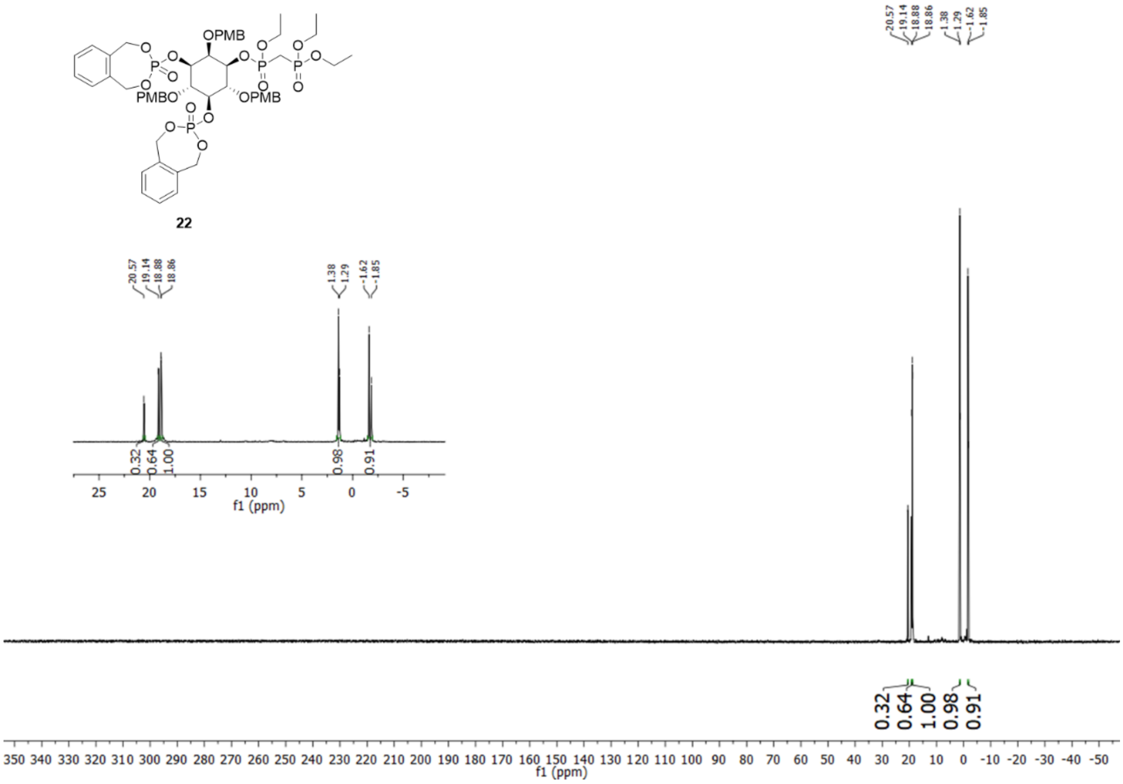

^{31}P spectrum of compound **22**.

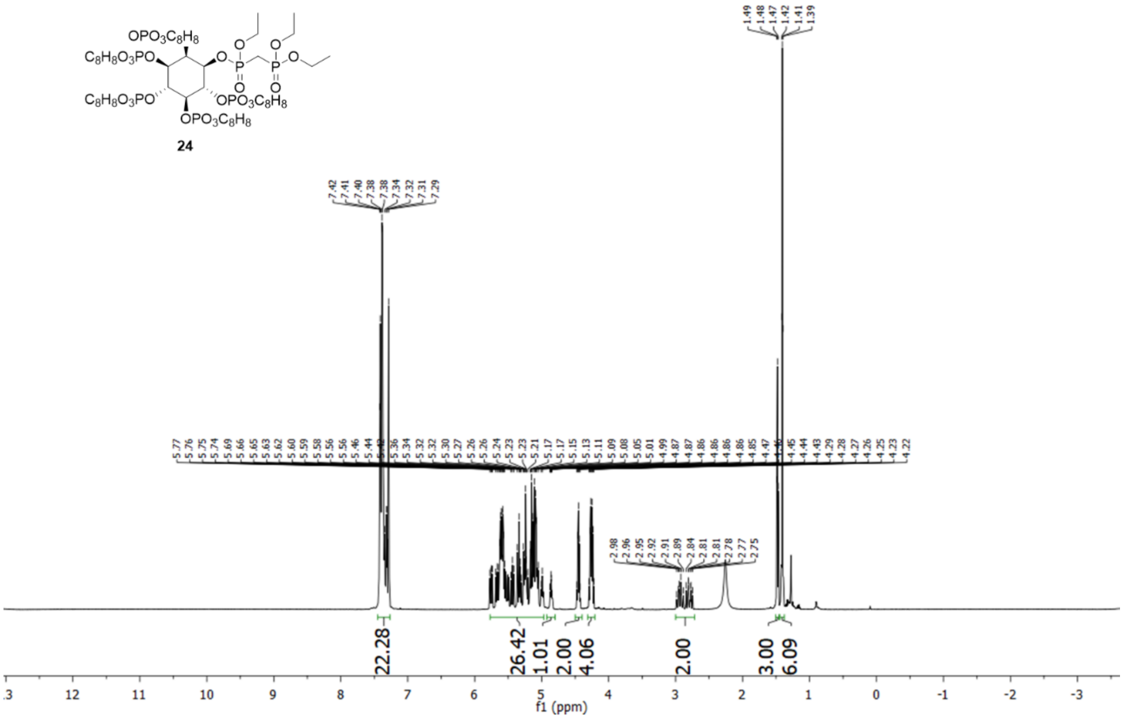

^{1}H spectrum of compound **24**.

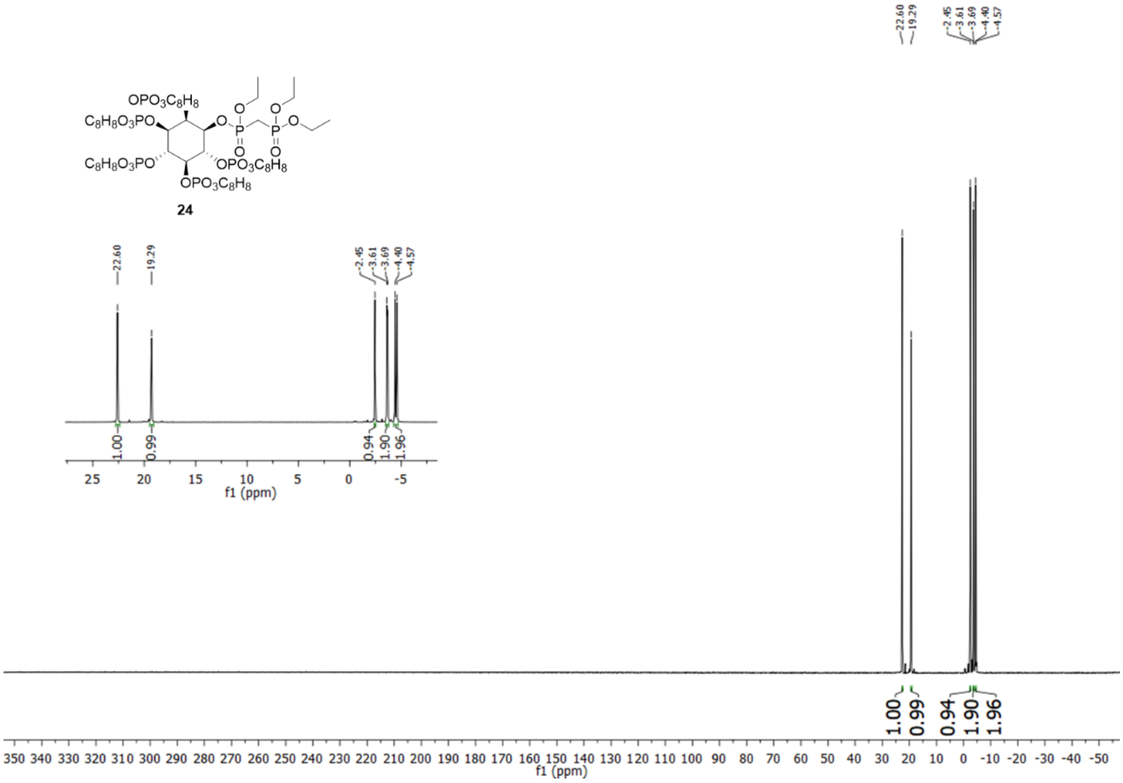

^{31}P spectrum of compound **24**.

References

1. Furkert, D., Hostachy, S., Nadler-Holly, M. & Fiedler, D. Triplexed Affinity Reagents to Sample the Mammalian Inositol Pyrophosphate Interactome. *Cell Chemical Biology* **27**, 1097-1108.e4 (2020).
2. Hostachy, S. *et al.* Dissecting the activation of insulin degrading enzyme by inositol pyrophosphates and their bisphosphonate analogs. *Chem. Sci.* **12**, 10696–10702 (2021).
3. Hulsen, T. DeepVenn -- a web application for the creation of area-proportional Venn diagrams using the deep learning framework Tensorflow.js. Preprint at https://doi.org/10.48550/arXiv.2210.04597 (2022).
4. Puschmann, R., Harmel, R. K. & Fiedler, D. Scalable Chemoenzymatic Synthesis of Inositol Pyrophosphates. *Biochemistry* **58**, 3927–3932 (2019).
5. Wu, M. *et al.* Elucidating Diphosphoinositol Polyphosphate Function with Nonhydrolyzable Analogues. *Angewandte Chemie International Edition* **53**, 7192–7197 (2014).
6. Hostachy, S. *et al.* Dissecting the activation of insulin degrading enzyme by inositol pyrophosphates and their bisphosphonate analogs. *Chem. Sci.* **12**, 10696–10702 (2021).
7. Wu, M., Chong, L. S., Perlman, D. H., Resnick, A. C. & Fiedler, D. Inositol polyphosphates intersect with signaling and metabolic networks via two distinct mechanisms. *Proceedings of the National Academy of Sciences* **113**, E6757–E6765 (2016).
8. Li, X. *et al.* Control of XPR1-dependent cellular phosphate efflux by InsP8 is an exemplar for functionally-exclusive inositol pyrophosphate signaling. *Proc Natl Acad Sci U S A* **117**, 3568–3574 (2020).
9. Wang, J. *et al.* Calcium sensitive ring-like oligomers formed by synaptotagmin. *Proc Natl Acad Sci U S A* **111**, 13966–13971 (2014).
10. Capolicchio, S., Thakor, D. T., Linden, A. & Jessen, H. J. Synthesis of unsymmetric diphospho-inositol polyphosphates. *Angew Chem Int Ed Engl* **52**, 6912–6916 (2013).
11. Hager, A. *et al.* Cellular Cations Control Conformational Switching of Inositol Pyrophosphate Analogues. *Chemistry – A European Journal* **22**, 12406–12414 (2016).
12. Couto, D. *et al.* Using Biotinylated myo-Inositol Hexakisphosphate to Investigate Inositol Pyrophosphate–Protein Interactions with Surface-Based Biosensors. *Biochemistry* **60**, 2739–2748 (2021).
13. Sauer, K., Huang, Y. H., Lin, H., Sandberg, M. & Mayr, G. W. Phosphoinositide and Inositol Phosphate Analysis in Lymphocyte Activation. *Current Protocols in Immunology* **87**, 11.1.1-11.1.46 (2009).
