# Supplementary material for: Proteome-wide quantification of inositol pyrophosphate-protein interactions
Source: Nat Commun. 2026 Jun 4;17:4967. doi: 10.1038/s41467-026-73804-8 (PMC13237142; doi:10.1038/s41467-026-73804-8)
Supplement: Supplementary file 2 — Description of Additional Supplementary Files [file 41467_2026_73804_MOESM2_ESM.pdf]

Description of Additional Supplementary Files

File Name: Supplementary Data 1 - Proteomics results all conditions

Description: Complete affinity determination results for all quantified proteins prior to filtering. Protein abundances were exported from Proteome Discoverer, normalized to the maximum value per replicate, and averaged across at least two quantified replicates. Dose-response curves were fitted to a five-parameter log-logistic model (LL.5, drc R package) to derive apparent dissociation constants (K_D^{app}). Model significance was assessed by a one-sided F-test (LL.5 vs. null model), with p-values adjusted using the Benjamini-Hochberg FDR method. No filters were applied.

File Name: Supplementary Data 2 - Relative quantification - All conditions probe vs probe

Description: Probe vs probe relative quantification. Protein abundances from affinity enrichment of b-$InsP_6$, b-1PCP-$InsP_5$, b-5PCP-$InsP_5$ and b-1,5$(PCP)_2$-$InsP_4$ (33.3 µM) in HEK293T cytosolic and nuclear fractions under Mg^{2+}/EDTA conditions were processed in R (v4.5.0). Proteins quantified in at least 2 of 3 replicates were retained, zero intensities set to NA, and values $\log_2$-transformed. Median normalization was applied to reduce sample-level bias and TMT-batch effects (limma package). Differential abundance between baits was assessed using two-sided moderated t-tests with empirical Bayes variance moderation, with p-values adjusted using the Benjamini-Hochberg FDR method. Significance was defined as $|\log_2(\text{fold change})| > 1$ and FDR < 0.05. No filters were applied.

File Name: Supplementary Data 3 - GO terms

Description: Complete gene ontology (GO) analysis for the proteins enriched by b-5PCP-$InsP_5$ and b-1,5$(PCP)_2$-$InsP_4$ (K_D^{app} < 5 µM, Mg^{2+}_{nucl}). The enrichment analyses were performed using Enrichr, applying a one-sided Fisher's exact test with Benjamini–Hochberg FDR correction for multiple testing.

File Name: Supplementary Data 4 - README - Code and Software
