# Supplementary material for: Proteome-wide quantification of inositol pyrophosphate-protein interactions
Source: Nat Commun. 2026 Jun 4;17:4967. doi: 10.1038/s41467-026-73804-8 (PMC13237142; doi:10.1038/s41467-026-73804-8)
Supplement: Supplementary file 7 — Reporting Summary [file 41467_2026_73804_MOESM7_ESM.pdf]

nature portfolio

Corresponding author(s): David Furkert
Dorothea Fiedler

Last updated by author(s): Apr 29, 2026

Reporting Summary

Nature Portfolio wishes to improve the reproducibility of the work that we publish. This form provides structure for consistency and transparency in reporting. For further information on Nature Portfolio policies, see our Editorial Policies and the Editorial Policy Checklist.

Statistics

For all statistical analyses, confirm that the following items are present in the figure legend, table legend, main text, or Methods section.

n/a	Confirmed	
☐	☒	The exact sample size (*n*) for each experimental group/condition, given as a discrete number and unit of measurement
☐	☒	A statement on whether measurements were taken from distinct samples or whether the same sample was measured repeatedly
☐	☒	The statistical test(s) used AND whether they are one- or two-sided<br>*Only common tests should be described solely by name; describe more complex techniques in the Methods section.*
☒	☐	A description of all covariates tested
☒	☐	A description of any assumptions or corrections, such as tests of normality and adjustment for multiple comparisons
☐	☒	A full description of the statistical parameters including central tendency (e.g. means) or other basic estimates (e.g. regression coefficient) AND variation (e.g. standard deviation) or associated estimates of uncertainty (e.g. confidence intervals)
☐	☒	For null hypothesis testing, the test statistic (e.g. *F*, *t*, *r*) with confidence intervals, effect sizes, degrees of freedom and *P* value noted<br>*Give P values as exact values whenever suitable.*
☒	☐	For Bayesian analysis, information on the choice of priors and Markov chain Monte Carlo settings
☒	☐	For hierarchical and complex designs, identification of the appropriate level for tests and full reporting of outcomes
☐	☒	Estimates of effect sizes (e.g. Cohen's *d*, Pearson's *r*), indicating how they were calculated

Our web collection on statistics for biologists contains articles on many of the points above.

Software and code

Policy information about availability of computer code

Data collection	Mass spectrometry data were measured using the Orbitrap Fusion Tribrid mass spectrometer device (Thermo Fisher Scientific) and Instrument Control Software version 4.0;<br>NMR spectra were recorded on a Bruker AV-600 (600 MHz) instrument;<br>Images/Western Blots were recorded using BioRad universal hood 3<br>Codes were deposited in Zenodo: https://zenodo.org/records/18375920
Data analysis	The R scripts used for the analysis of quantitative proteomic data are available via Zenodo at https://zenodo.org/records/18375920 -<br>R 4.5.2;<br>R studio 2025.09.2;<br>Proteome Discoverer 3.0 (Thermo Fisher Scientific);<br>MestReNova v.10.0.2; Image Lab 6.1.0<br>Scansite4.0; IUPred3; Prism 10.5.0

For manuscripts utilizing custom algorithms or software that are central to the research but not yet described in published literature, software must be made available to editors and reviewers. We strongly encourage code deposition in a community repository (e.g. GitHub). See the Nature Portfolio guidelines for submitting code & software for further information.

Data

Policy information about availability of data

All manuscripts must include a data availability statement. This statement should provide the following information, where applicable:

- Accession codes, unique identifiers, or web links for publicly available datasets
- A description of any restrictions on data availability
- For clinical datasets or third party data, please ensure that the statement adheres to our policy

The mass spectrometry raw data and ProteomeDiscoverer outputs were deposited with the ProteomeXchange consortium partner repository jPOSTrep66 under the accession code JPST003145 and PXD052682.
Reviewer access: https://repository.jpostdb.org/preview/2101742205665782f5915de
Source Data are provided with this paper.

Research involving human participants, their data, or biological material

Policy information about studies with human participants or human data. See also policy information about sex, gender (identity/presentation), and sexual orientation and race, ethnicity and racism.

Reporting on sex and gender	not applicable
Reporting on race, ethnicity, or other socially relevant groupings	not applicable
Population characteristics	not applicable
Recruitment	not applicable
Ethics oversight	not applicable

Note that full information on the approval of the study protocol must also be provided in the manuscript.

Field-specific reporting

Please select the one below that is the best fit for your research. If you are not sure, read the appropriate sections before making your selection.

☒ Life sciences ☐ Behavioural & social sciences ☐ Ecological, evolutionary & environmental sciences

For a reference copy of the document with all sections, see nature.com/documents/nr-reporting-summary-flat.pdf

Life sciences study design

All studies must disclose on these points even when the disclosure is negative.

Sample size	Sample sizes were chosen based on our prior experience on the assays/experiments performed in this study (Furkert et. al., Cell Chemical Biology, 2020; Kliza et al., Molecular Cell, 2021; Morgan et al., Nature Chemical Biology, 2024)
Data exclusions	No data was excluded from the analysis.
Replication	As per standard practices in Cell biology and Biochemistry, experiments were conducted in independent biological replicates. The number of replicates in each dataset is indicated in the respective figure legend. All attempts at replication were succesfull.
Randomization	Randomization is not applicable.
Blinding	Blinding is not applicable.

Reporting for specific materials, systems and methods

We require information from authors about some types of materials, experimental systems and methods used in many studies. Here, indicate whether each material, system or method listed is relevant to your study. If you are not sure if a list item applies to your research, read the appropriate section before selecting a response.

Materials & experimental systems

n/a	Involved in the study
☐	☒ Antibodies
☐	☒ Eukaryotic cell lines
☒	☐ Palaeontology and archaeology
☒	☐ Animals and other organisms
☒	☐ Clinical data
☒	☐ Dual use research of concern
☒	☐ Plants

Methods

n/a	Involved in the study
☒	☐ ChIP-seq
☒	☐ Flow cytometry
☒	☐ MRI-based neuroimaging

Antibodies

Antibodies used

INPP5K (Proteintech, Cat# 15098-1-AP, Dil: 1:500);
COPS5/JAB1 (Proteintech, Cat# 27511-1-AP, Dil: 1:500);
SYT1 (Proteintech, Cat# CL488-68043, Dil: 1:1000);
PRPS1 (Proteintech, Cat# 15549-1-AP, Dil: 1:1000);
NUDT3(DIPP1) (Proteintech, Cat# 20542-1-AP, Dil: 1:1000);
NAMPT/PBEF (Proteintech, Cat# 66385-1-IG, Dil: 1:5000),
HRP-conjugated 6*His (Proteintech, HRP-66005, Dil: 1:10000)
STK24(MST3) (Thermo Fisher Scientific, Cat# PA5-120097, Dil: 1:1000);
FAK (PTK2) (Thermo Fisher Scientific, Cat# PA5-17591, 1:150);
PMVK (Absea, Cat# P02302PA, Dil: 1:1000);
Polyclonal rabbit HRP (Cell Signalling Technology, Cat# 7074; RRID: AB_2099233, Dil: 1:1000);
Polyclonal mouse HRP (Cell Signalling Technology, Cat#7076; RRID: AB_330924, Dil: 1:1000);
FUS (Santa Cruz, Cat# sc-47711, Dil: 1:1000);
b-actin (Santa Cruz, Cat# sc-47778, Dil: 1:1000);
a-tubulin (Santa Cruz, Cat# sc-8035, Dil: 1:1000)

Validation

Antibody information can be found in the respective manufacturer's web sites.

Eukaryotic cell lines

Policy information about cell lines and Sex and Gender in Research

Cell line source(s)	HEK293T ATCC CRL-3216
Authentication	HEK293T cells were authenticated using STR profiling.
Mycoplasma contamination	All cell-lines tested negative by PCR.
Commonly misidentified lines (See ICLAC register)	None of the cell lines used in this study are listed as commonly misidentified lines.

Plants

Seed stocks	not applicable
Novel plant genotypes	not applicable
Authentication	not applicable
